# Compendium of RNA modifications for bacterial stress adaptation

**DOI:** 10.1128/mmbr.00374-25

**Published:** 2026-07-28

**Authors:** Siobhan A. Cusack, Sebastián Riquelme-Barrios, Luis Rivera-Montero, Leonardo Vásquez-Camus, Dania Devassy, Kirsten Jung

**Affiliations:** 1 Faculty of Biology, Microbiology, Ludwig-Maximilians-Universität München 9183, Martinsried, Germany; Collège de France, Paris, France

**Keywords:** rRNA, mRNA, tRNA, epitranscriptome, environmental stress

## Abstract

RNA modifications in the tRNA, rRNA, and mRNA constitute a widespread layer of post-transcriptional gene regulation, commonly known as the epitranscriptome. In bacteria, tRNA and rRNA modifications are well documented and play essential roles in tRNA folding, rRNA maturation, translation, and peptidyl transfer. Although mRNA modifications have been extensively characterized in eukaryotes, their functional implications for mRNA fate in prokaryotes remain uncertain, and their identities and locations are the subject of an emerging field of research. Importantly, RNA modification is a dynamic process, and variation in the presence and abundance of these modifications has significant consequences for bacterial physiology. Here, we discuss current knowledge of modified RNA nucleotides in bacteria, with a particular focus on their roles in bacterial adaptation to environmental stress.

## INTRODUCTION

**Fig 1:**
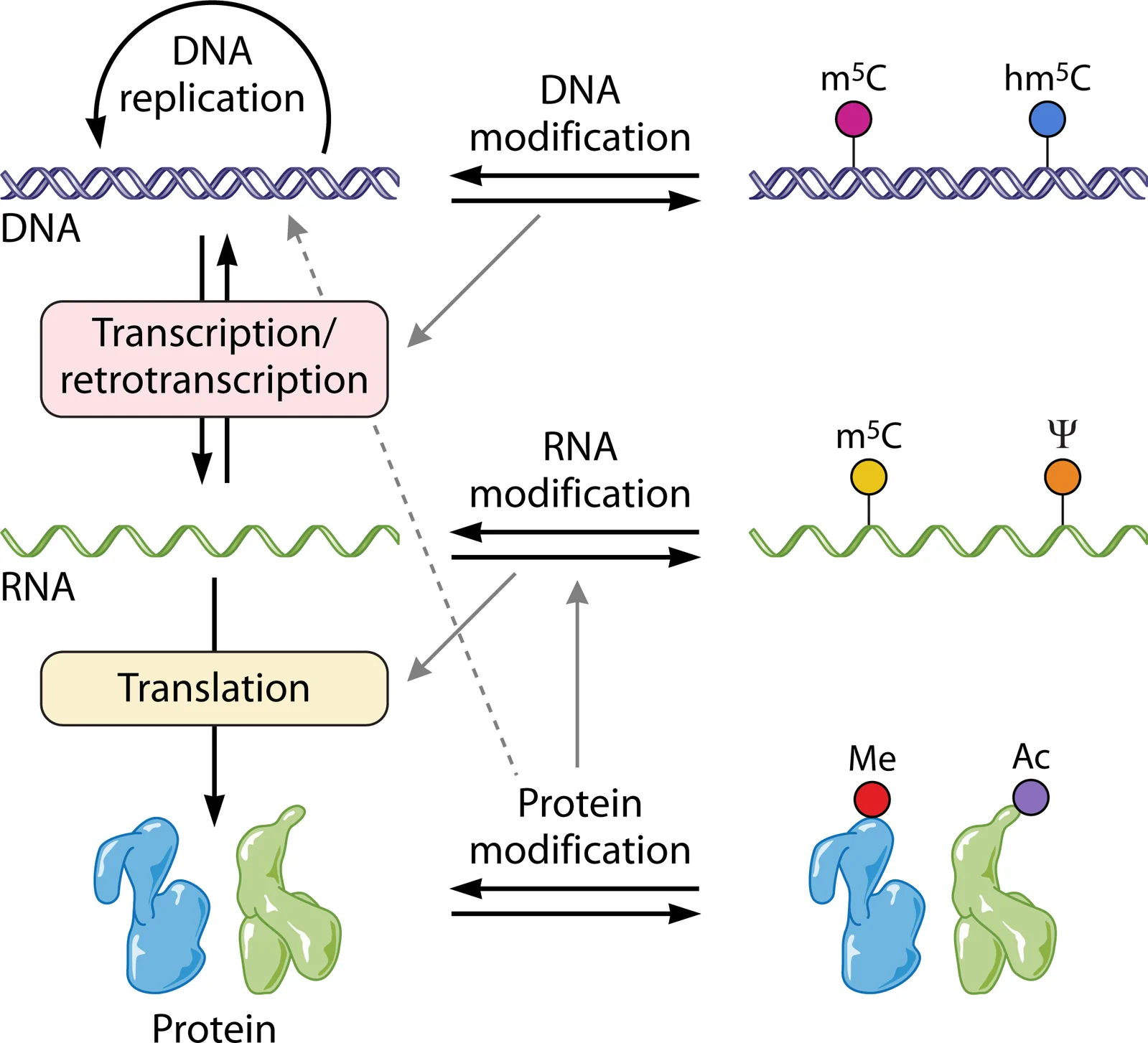
The expanded central dogma of molecular biology. Rather than a consistent flow of information from DNA to RNA and from RNA to protein, there is a bidirectional flow of information due to the presence of modifications in the DNA, RNA, and proteins. The dashed arrow represents the bacterial epigenome (5).

The first RNA modification described in the literature, pseudouridine (Ψ), was characterized in the mid-20th century (1) and was initially thought to be a fifth nucleotide. Since then, more than 170 RNA modifications have been identified, expanding our knowledge of the functions and physical properties associated with RNA (2). Although the presence of these modifications is well documented, their functionalities have long been underestimated; nucleotides are commonly seen as merely building blocks and intermediates in the flow of genetic information. This view has been reinforced over the last few decades by the widespread focus on omics approaches, which consider RNAs primarily as carriers of information from DNA, often overlooking RNA modifications entirely. It was not until the past decade that the idea of modifications as versatile regulators of RNA fate gained consensus within the community, with concepts such as RNA modification dynamics and epitranscriptomics coming to the fore (3, 4) (Fig. 1).

The field of epitranscriptomics was greatly influenced by studies of *N*^6^-methyladenosine (m^6^A), the most prevalent internal messenger RNA (mRNA) modification in eukaryotes (6). Three distinct types of m^6^A enzymes have been discovered: the so-called writers, readers, and erasers. First discovered were the erasers, such as FTO (7) and ALKBH5 (8), which are responsible for m^6^A removal. Next were the writers (including the METTL3-METTL14 methyltransferase complex [9]), which add the methyl group to form m^6^A. Last were the readers, such as YTHDF1, YTHDF2, and YTHDF3 (10–12), which recognize the presence of m^6^A in RNA. Together, these discoveries revealed a complex, dynamic system of m^6^A deposition in the mRNA, greatly expanding our view of modifications as a regulatory feature in RNA.

Although lagging behind knowledge in eukaryotes, the existence of RNA modifications in bacteria has long been known (13). However, the roles of these modifications and the complex mechanisms underlying their regulation remain poorly understood. Generally, bacterial RNA modifications are thought to be added enzymatically in a co-transcriptional or post-transcriptional manner, in contrast to modifications that are produced directly by stress without enzymatic intervention (e.g., formation of 8-hydroxyguanosine due to oxidative stress [14]). Studies of prokaryotic RNA modifications, primarily in the model bacterium *Escherichia coli*, have yielded a thorough understanding of modification types and locations in transfer RNAs (tRNAs) and ribosomal RNAs (rRNAs), and even some links to specific functions in RNA assembly, structure, maturation, codon recognition, peptidyl transferase activity, and translation fidelity (15–21). With respect to mRNAs, there are indications that bacterial mRNA modifications are associated with reading accuracy (22–24). However, studies in this area are still in their infancy and are broadly limited by technical difficulties, including inefficient ribosome depletion methods (25) and a lack of methods for orthogonal validation (26).

The field of bacterial epitranscriptomics has recently benefited from the development of direct RNA-sequencing (DRS) techniques, which enable the evaluation of native-form RNAs. DRS approaches have been used to examine the changes in several *E. coli* RNA subtypes in response to various stressors. For example, previous studies have assessed *E. coli* rRNA after exposure to metabolic stress, cold shock (20°C) (27), or aminoglycoside treatment (28); the effects of T4 phage infection on tRNA have also been determined (29). Recent analyses of epitranscriptomic changes in the tRNA, rRNA, and mRNA of *E. coli* under heat (45°C), acid (pH 4.4), and oxidative (2 mM H_2_O_2_) stress (30, 31) have revealed new insights into changes occurring at multiple levels, enhancing our understanding of the regulatory complexity associated with modifications. Moreover, a recent analysis demonstrated that the tRNA maturation process is strongly affected by oxidative and acid stress conditions, whereas modification levels in mature tRNAs remain largely unchanged (31).

Although the levels of some RNA modifications apparently fluctuate in response to stress conditions (31), and increases can be easily explained by the activity of RNA modification writer enzymes, the mechanisms underlying decreases are largely unclear. Due to the rarity of RNA modification eraser enzymes identified in bacteria, the processes leading to apparent decreases are likely more circuitous than direct enzyme activity. For example, lowered modification abundance in the tRNA is most likely due to alterations in tRNA processing; an increase in the levels of the immature, unmodified form of a pre-tRNA will reduce the relative abundance of modifications that are found only in mature tRNAs (31). Decreases in the presence or activity of modification writer enzymes will also result in decreased tRNA modification levels over time (32, 33). Thus, even in the absence of RNA modification eraser enzymes, RNAs may show apparent decreases in modification abundance. Future biochemical studies in bacteria may also identify previously uncharacterized modification eraser enzymes (34).

A major bottleneck in the field of bacterial epitranscriptomics is the widespread use of a single model organism. Most of what is currently known about bacterial RNA modifications, particularly their dynamics and roles in stress responses, comes from studies in *E. coli*. This focus has been invaluable for developing tools and conceptual frameworks, but it also constrains our view of how RNA modifications operate across the domain *Bacteria*. Increasing evidence from thermophiles, gram-positive pathogens, and environmental isolates shows that many RNA modifications are conserved, but modulated in distinct ways that reflect unique ecological and physiological pressures. However, in most of these organisms, RNA modifications have been cataloged without the same level of mechanistic or functional resolution that has been achieved in *E. coli*. As a result, we cannot yet distinguish between universal principles of bacterial epitranscriptomic regulation and mechanisms that are tailored to specific lifestyles, stress niches, or evolutionary histories.

Recent reviews have summarized current knowledge of bacterial modifications with respect to stress responses, host adaptations, and virulence (18, 35). However, each of these reviews focuses on a specific RNA species or bacterial type. Here, we first provide an overview of methods by which RNA modifications can be detected in bacteria, with more detailed descriptions of recent advances. We then summarize current understanding of RNA modifications across bacterial phyla and RNA subtypes, with a focus on rRNA, tRNA, and mRNA, highlighting the roles of modifications in stress adaptation. Our current understanding of RNA modifications as a dynamic component of bacterial physiology is the conceptual axis of this review. Recent results demonstrate the role of the epitranscriptome as a novel regulatory layer, highlighting the necessity of integrating information about all bacterial modifications in multiple RNA subtypes and across species to fully understand their impacts on survival and stress responses.

## METHODS FOR RNA MODIFICATION DETECTION

Bacterial RNA modifications are detectable using a variety of methods, which can be broadly categorized by their technological basis: chemical/enzymatic, mass spectrometry (MS), immunoprecipitation, PCR, and DRS. Each family of methods presents distinct advantages and disadvantages. Although many detection methods have been reviewed previously (36, 37), the challenges specific to studies of prokaryotes, such as difficulties with RNA purification and ribosome depletion compared to eukaryotes, are often overlooked. Furthermore, several important advances have been made recently in the areas of PCR-based and DRS-based detection that warrant particular emphasis. With a focus on bacteria, this section provides a brief overview of popular methods used for the detection of RNA modifications and more detailed explanations of recent advances. A summary of the methods discussed here can be found in Table 1.

**TABLE 1:** Summary of modification detection methods discussed in the present work

Method^*a*^	Target modification(s)	Methodological basis	Reference(s)
Bisulfite sequencing	m^5^C	Chemical/enzymatic	Frommer et al.; Schaefer et al.; Johnson et al. (38–40)
GLORI	m^6^A	Chemical/enzymatic	Liu et al.; Sun et al.; Hardy et al. (41–43)
eTAM-seq	m^6^A	Chemical/enzymatic	Xiao et al.; Szydlo et al. (44, 45)
MAZTER-seq	m^6^A	Chemical/enzymatic	Garcia-Campos et al. (46)
AlkAniline-Seq	m^7^G, m^3^C	Chemical/enzymatic	Marchand et al. (47)
HydraPsiSeq	Ψ	Chemical/enzymatic	Marchand et al. (48)
BID-seq	Ψ	Chemical/enzymatic	Dai et al. (49)
CMC labeling	Ψ	Chemical/enzymatic	Bakin and Ofengand (50)
N/A	m^6^A, m^6^Am	Mass spectrometry	Sun et al. (51)
Mass ladder sequencing	Any modification type	Mass spectrometry	Bahr et al.; Zhang et al.; Yuan et al. (52–54)
meRIP-seq	m^6^A, m^7^G, ac^4^C	Antibody	Meyer et al.; Lin et al.; Arango et al. (55–57)
m^5^C RNA immunoprecipitation	m^5^C	Antibody	Edelheit et al. (58)
m^6^A-CLIP	m^6^A	Antibody	Ke et al. (59)
miCLIP	m^6^A, m^6^Am	Antibody	Linder et al. (60)
meCLIP	m^6^A	Antibody	Roberts et al. (61)
m6ACE-seq	m^6^A	Antibody	Koh et al. (62)
SELECT	m^6^A	PCR	Xiao et al. (63)
m^6^A-Rol-LAMP	m^6^A	PCR	Li et al. (64)
m^5^C-Rol-LAMP	m^5^C	PCR	Vásquez-Camus et al. (65)
RhoRT-PCR	D	PCR	Toubdji et al. (66)
N/A	Nm	PCR	Elliott and Holley (67)
Templated-ligation qPCR	m^1^A	PCR	Zhang et al. (68)
BIHIND	Ψ	PCR	Zhao et al. (69)
pseU-TRACE	Ψ	PCR	Fang et al. (70)
Nanocompore	Any modification type	DRS	Leger et al. (71)
CHEUI	m^6^A, m^5^C	DRS	Acera Mateos et al. (72)
DiffErr	m^6^A	DRS	https://github.com/bartongroup/differr_nanopore_DRS
ELIGOS	Any modification type	DRS	Jenjaroenpun et al. (73)
DRUMMER	Any modification type	DRS	Abebe et al. (74)
Dorado basecaller models	m^5^C, m^6^A, I, Ψ, Cm, Am, Gm, Um	DRS	https://software-docs.nanoporetech.com/dorado/latest/basecaller/mods/
nanoSundial	Any modification type	DRS	Guo et al. (75)
N/A	Any modification type	DRS	White et al. (29)

^*a*^ Methods marked N/A were not given a name in the corresponding publication.

### Chemical and enzymatic methods

A great deal of research attention has focused on the detection of 5-methylcytosine (m^5^C) due to the prevalence of this modification in eukaryotic systems. The classic approach for m^5^C detection is bisulfite sequencing (BS-seq), as originally described in 1992 (38). Nucleic acids are treated with bisulfite, which converts unmodified cytosine to uracil while leaving m^5^C unchanged. Treated samples are then prepared and sequenced (typically on an Illumina platform in the present day); the presence of cytosine in the resulting data indicates the presence of m^5^C in the original sample. Due to the high pH of the bisulfite treatment and the relative sensitivity of RNA compared to DNA, this approach was long considered unsuitable for the analysis of RNA modifications. However, Schaefer et al. (39) developed a modified method in 2009 that allowed for reliable bisulfite treatment of RNAs. Later optimization efforts (40) improved the accuracy of BS-seq approaches, enabling large-scale implementation of such methods for m^5^C detection in RNA.

Several protocols for m^6^A detection have also been developed, reflecting the importance of this extremely abundant modification in eukaryotes. Examples include glyoxal- and nitrite-mediated deamination of unmethylated adenosines (GLORI) (41), a method that is functionally comparable to BS-seq. Here, chemical treatment causes canonical A sites to undergo deamination to inosine (I), whereas m^6^A sites are protected from conversion. Using Illumina-type sequencing, the resulting I sites are read as Gs, and A nucleotides therefore correspond to m^6^A in the original sample. Later improvements to the method resulted in decreased RNA degradation and increased sensitivity (42). Importantly, GLORI has been validated in bacteria such as *Vibrio cholerae* (43). A conceptually related approach for m^6^A detection (using enzymatic instead of chemical deamination) is referred to as evolved TadA-assisted N6-methyladenosine sequencing (eTAM-seq) (44). eTAM-seq has also successfully been applied in bacteria (45), although there is some evidence of lower A-to-I deamination rates compared to GLORI (42). MAZTER-seq (46) is an m^6^A detection technique employing the bacterial RNase MazF, which cleaves RNA directly upstream of unmethylated ACA sequences while leaving the region upstream of m^6^ACA intact. Sequencing of the resulting RNA allows the inference of m^6^A sites based on the abundance of RNA fragments ending at the expected cleavage site. This method enables the detection of m^6^A in the context of a motif that is relatively common in methylated eukaryotic RNA (6) but is not expected to have the same performance in bacterial samples due to differences in methylation sequence contexts.

Additional chemical and enzymatic methods have been developed to detect a handful of other modification types. For example, AlkAniline-Seq (47) uses a series of alkaline hydrolysis, dephosphorylation, and aniline cleavage steps prior to RNA library preparation, allowing the detection of both N7-methylguanosine (m^7^G) and 3-methylcytidine (m^3^C) in prokaryotes and eukaryotes. A similar method developed by the same group, HydraPsiSeq (48), employs hydrazine, dephosphorylation, and aniline treatment prior to sequencing to infer the presence of Ψ in RNA at single-base resolution. A more recently optimized addition to this family of techniques is BID-seq (49), which uses bisulfite to detect Ψ rather than m^5^C. This method relies on the fact that bisulfite forms an adduct with Ψ that prevents reverse transcription (RT) of Ψ–bisulfite sites. This causes single-site deletions in the resulting cDNA, which can be detected with sequencing. A related but less refined method for Ψ detection was developed by Bakin and Ofengand over three decades ago (50). Using this approach, samples are treated with N-cyclohexyl-N′-β-(4-methylmorpholinium)ethylcarbodiimide p-tosylate (CMC), which reacts with G and U nucleotides and their derivatives. Subsequent exposure to mild alkaline conditions reverses the CMC addition to G and U, but not to Ψ; the presence of the modified base can be inferred from RT product size due to RT blockage by the Ψ–CMC adduct. These techniques offer a range of options for Ψ detection that vary in complexity and cost.

### Mass spectrometric methods

MS approaches have been considered the gold standard for RNA modification detection. These methods rely on the fact that most RNA modifications produce a detectable shift in molecular mass compared to the corresponding canonical base. Even Ψ can be indirectly detected with the application of a molecular tag that produces a distinct fragmentation pattern (eg., as described by Patteson et al. [76]). Relevant methods for the detection of RNA modifications via MS have previously been reviewed (77), but the past several years have seen important methodological improvements.

Many MS-based approaches are applied to bulk RNA, which produces difficulties in precisely localizing modifications and in quantifying modification abundance at particular sites. Notably, one group developed a method for the detection of m^6^A and *N*^6^,2′-*O*-dimethyladenosine (m^6^Am) with single-base specificity (51). This method relies on RNA fragmentation followed by antibody- and DNA probe-based enrichment. Although it allows for distinction between two closely related modifications with single-base resolution, the need for DNA probes specific to individual sequences limits throughput. Nevertheless, it is an important advance in available MS-based modification detection methods.

Mass ladder sequencing has emerged as a method of analyzing both RNA sequences and modifications simultaneously (52, 53). In this approach, an RNA region of interest is cleaved to produce every possible fragment at intervals of one nucleotide, yielding a “ladder” of RNA products. The data collected from liquid chromatography (LC)-MS of these fragments can then be combined to infer the order of bases and the presence of modifications. This approach thus gives information about modifications with single-base resolution. However, biases in steps such as RNA hydrolysis or MS can prevent the attainment of a true base-by-base ladder (53), complicating analysis efforts. MS ladder complementation (MLC)-seq (54) combines next-generation sequencing with mass ladder sequencing, enabling robust analyses even in the absence of a perfect mass ladder. Approaches such as this still face challenges, such as requirements for a large amount of RNA and biases against lowly abundant modifications, but represent a significant step toward accurate single-base RNA modification analysis.

Overall, MS is the ground-truth standard for validation and quantification of RNA modifications in many contexts. The restriction of most methods to the analysis of bulk RNA remains a significant hurdle, highly limiting resolution. Furthermore, it may be difficult to distinguish between modifications with highly similar properties using MS-based methods. Ongoing research is expected to build on recent advances, enabling site-specific detection of specific modification types, extending the utility of MS-based approaches.

### Antibody-based methods

Antibody-based RNA modification detection methods are typified by methylated RNA immunoprecipitation sequencing (meRIP-seq), which was developed by Meyer et al. (55). This protocol involves RNA fragmentation, immunoprecipitation with m^6^A-specific antibodies, and then sequencing of the resulting m^6^A-containing fragments. Aligned fragments show a peak in read depth around the modified sites, thus indicating the presence of a modification within a region of ~100–200 nt. A comparable method has also been used for the detection of m^5^C in bacterial RNA (58), and numerous RNA modifications have been detected in eukaryotic RNA via immunoprecipitation-based methods (e.g., m^7^G (56) and *N*^4^-acetylcytidine [ac^4^C] [57]).

Despite the prevalence of basic antibody-based modification detection methods, there are several issues with early iterations of these approaches, including low resolution and a lack of information about modification stoichiometry. To increase resolution, several methods employing ultraviolet (UV) cross-linking prior to immunoprecipitation (CLIP) were developed (e.g., m^6^A-CLIP [59] miCLIP [60]. Combining several such advances, meCLIP (61) employs UV cross-linking of m^6^A antibodies to fragmented RNA, followed by protein purification, proteinase digestion, RT, and sequencing; C-to-T mutations caused by the presence of an anti-m^6^A antibody can be used to identify m^6^A sites with single-nucleotide specificity. A related method, m6ACE-seq (62), also employs anti-m^6^A antibodies, relying on the protection of antibody-bound RNA fragments from exoribonuclease activity. This approach offers some improvements over the past methods for m^6^A detection. Despite the increased resolution yielded by such protocols, antibody-based detection methods overall remain less reliable than other approaches due to high false-positive rates and cross-reactivity with related modifications (60). Nevertheless, they may serve as valuable starting points for epitranscriptome-wide identification of sites to prioritize for orthogonal validation.

### PCR-based methods

In contrast to standard MS-based and older antibody-based methods of RNA modification detection, PCR-based methods enable detection with single-base resolution. These approaches rely on differences in the physical properties of modified compared to canonical bases during replication or transcription *in vitro*. Standard PCR or quantitative PCR (qPCR) can then be used to detect the presence and, with some methods, relative abundance of a modification.

One of the earliest PCR-based RNA modification detection methods developed was the single-base elongation- and ligation-based PCR amplification method (SELECT) (63), which enables the detection of m^6^A. This approach yields information about the presence of the modification because m^6^A, but not canonical A, inhibits DNA elongation and nick ligation. This, in turn, leads to lower amplification of regions that contain m^6^A compared to unmodified A during qPCR, which translates to a higher threshold cycle (Ct) value. Importantly, SELECT can be used not only to detect the presence or absence of a modification but also to estimate the fraction of modified RNAs in a sample when appropriate unmodified and modified controls are included.

In 2023, Li et al. (64) described another PCR-based method of detecting m^6^A, known as m^6^A rolling circle extension-assisted (Rol) loop-mediated isothermal amplification (LAMP). Comparable to SELECT, m^6^A-Rol-LAMP relies on steric hindrance of nick ligation by m^6^A. However, instead of using a pair of primers, m^6^A-Rol-LAMP uses a DNA “padlock probe” that is elongated and ligated into a single circular DNA molecule in the absence of m^6^A. This is followed by amplification of the circular DNA to form long single-stranded DNA, which is then further amplified via LAMP. Quantification via qPCR enables detection based on Ct values, with low- and high-Ct reactions corresponding to the presence and absence of a modification, respectively. Modification stoichiometry can also be determined based on the Ct values in a sample of interest compared to those produced by fully modified and fully unmodified samples. Furthermore, the presence of a modification can be determined by eye if the PCR product is mixed with a nucleic acid dye (e.g., SYBR Green or GoldView). Thus, advantages of m^6^A-Rol-LAMP include high sensitivity, the capacity to quantify the abundance of a modification of interest, and low cost.

Building on the Rol-LAMP approach, a recent study (65) describes a similar methodology that enables the detection of m^5^C, m^5^C-Rol-LAMP. In contrast to m^6^A-Rol-LAMP, this method integrates a bisulfite conversion step to discriminate between methylated and unmethylated cytidines, followed by separate reactions with different nucleotide mixtures (dATP only, dGTP only, or a combination of dATP and dGTP) during amplification. This design enables selective amplification depending on the methylation status of the target site and allows accurate relative quantification of m^5^C levels at single-nucleotide resolution (65). m^5^C-Rol-LAMP therefore retains the core advantages of the Rol-LAMP framework while extending its applicability to cytidine methylation and enhancing its quantitative capabilities.

Beyond m^5^C and m^6^A, a method for site-specific detection of dihydrouridine (D) at position 2449 of the 23S rRNA, RhoRT-PCR, was recently described (66). In this protocol, samples are treated with rhodamine 110 (rho), which binds to D. The resulting rho-labeled D causes RT blockage. To detect this blockage, PCR is carried out with two sets of primers: one pair in which both primers bind downstream of the site of interest, and one pair that includes the site of interest between the two primers. Samples containing unmodified U, which will not produce RT blockage, yield PCR products of two different sizes. In contrast, the presence of rho-labeled D blocks RT, leading to the production of a single PCR product generated by the downstream-only primer pair.

Less common methods have been developed to detect other types of mRNA modifications, with the molecular basis of each technique differing with the biochemical properties of the modification type in question. For example, 2′-*O*-methylation (Nm) can be detected due to Nm-induced inhibition of RT at low concentrations of dNTPs (67). N^1^-methyladenosine (m^1^A) can be detected with templated-ligation qPCR, which operates in a manner similar to SELECT (68). Two PCR-based techniques have been developed to detect Ψ using bisulfite treatment, BIHIND (69) and pseU-TRACE (70). There are important methodological differences between these two approaches: BIHIND relies on the prevention of single-base elongation and nick ligation by the bulky BS-Ψ adduct, which can be detected via qPCR. pseU-TRACE instead includes an RT step, which leads to a deletion mutation at the BS-Ψ adduct site. Specially designed primers are then employed that lead to amplification only if there has been a single-base deletion at the site of interest, that is, if the corresponding RNA contains BS-Ψ at that site.

PCR-based approaches offer several valuable advantages over other methods of RNA modification detection. They do not produce the high levels of technical artifacts and the associated false positives that are common among enzymatic and antibody-based methods. They offer high (single-base) resolution and do not require specialized equipment, radioactive components, or particularly expensive reagents. However, methods development can be extremely time-consuming; furthermore, individual methodologies are generally valid for just one modification type, and primers or probes will work only for a single site. Thus, *a priori* knowledge of both the modification type and location is required, limiting the capacity for novel site discovery. These methods are therefore most suitable for the orthogonal validation of modifications detected with other methods or for the relative quantification of modification levels.

### DRS-based methods

Nanopore platforms can be used for the direct sequencing of native RNA molecules. The raw electrical signals of modified bases differ from those produced by canonical bases, enabling the detection of modifications (78). However, this is complicated by the fact that the signal associated with each base also depends on the surrounding sequence context; as each base in an RNA strand passes through the nanopore, there are a total of nine nucleotides within the pore affecting the electrical signal (79). Thus, considering the four canonical RNA nucleotides, there are 4^9^ possible combinations of canonical bases that could be present in the nanopore (influencing the resulting signal) at any given time. This sheer number of possibilities means that the distinct signal associated with every combination of bases has yet to be thoroughly characterized, complicating data interpretation.

In nanopore sequencing data analysis, the identities of individual nucleotides are inferred from the raw electrical signals in a process called basecalling. Programs designed to identify RNA modifications from nanopore data have been released directly by Oxford Nanopore Technologies (ONT), and numerous contributions have also been made by the broader scientific community. Unfortunately for bacterial researchers, many of these programs (e.g., m6Anet, Nanom6A, Epinano, MINES, and Penguin [80–84]) are designed to identify modifications within eukaryotic consensus motifs that are not conserved in prokaryotes.

Broadly, modification detection programs fall into two primary categories: those that directly assess the raw electrical signals produced during sequencing, and those that calculate the frequency of errors made during basecalling (referred to as the basecalling error or BCError) compared to a reference sequence. Examples of the former are Nanocompore (71) and CHEUI (72), and those that rely on the latter include DiffErr (https://github.com/bartongroup/differr_nanopore_DRS), ELIGOS (73), and DRUMMER (74). Notably, BCError can be used as a proxy for modification abundance (27), allowing the calculation of between-sample differences in the proportion of RNAs with a modification at a particular site. Fleming et al. (27) demonstrated the capacity to detect 16 distinct modification types in *E. coli* rRNAs using a combination of detection programs and an integrated set of features such as the current intensity, dwell time, and BCError, demonstrating the value of combining programs with different underlying methodologies.

Another key difference in modification detection programs is related to the inclusion of a sequencing control. Programs such as ELIGOS, Nanocompore, and DRUMMER require a control sample, such as *in vitro*-transcribed (IVT) RNA (71, 73, 74). Subtraction of the signal (for current-based methods) or error (for BCError-based methods) produced by an unmodified base from that of the same position in a control sample yields only the sequencing error caused by a modification. In general, inclusion of an IVT control is a highly effective method of controlling for the inherent noise associated with nanopore sequencing due to variations in sequence context as described above (85).

ONT released an updated version of RNA flow cells and sequencing chemistry, RNA004, in early 2024. Although the lower sequencing error of this platform is widely considered a benefit, it significantly altered the BCError for many RNA modifications compared to data produced with the older RNA002 flow cells (29). The differences in raw electrical signals and BCError between versions have rendered many established workflows for the detection of RNA modifications unusable with RNA004 data (86). Filling this methodological gap, ONT released updated models for their basecalling software, Dorado, to directly integrate the detection of some modification types into the basecalling step. At least two studies using human cell lines have reported extremely high accuracy rates for the Dorado m^6^A model (86, 87), and one of these publications reported similarly high accuracy for the Ψ model. However, the Dorado modification models do not appear to have such high accuracy when applied to *E. coli* RNA (75), potentially indicating a lack of suitability for use with prokaryotic RNA at present.

RNA004-compatible modification detection tools are still being developed, and many are likely to be released in the next several years. Currently available tools include nanoSundial (75), a current-based program for modification detection. Following the published nanoSundial workflow, which includes merging of adjacent modification-containing regions, this program may produce results of relatively long regions likely to contain a modification (from around nine nucleotides to hundreds of nucleotides in length). The merging step can be omitted to yield results at single-base resolution. White et al. (29) released a script for the calculation of per-site BCError that is compatible with RNA004 data. In this case, the user must define a BCError threshold for the classification of sites as modified or unmodified. Using this approach, modification detection accuracy in *E. coli* rRNA was found by Riquelme-Barrios et al. (31) to be much higher when sites were only considered if they overlapped with regions classified as modified by nanoSundial. Together with the findings of Fleming et al. (27), these results highlight the importance of combining modification detection tools with distinct methodologies and validate the newly developed methods for bacterial RNA modification detection in RNA004 data sets.

In a comprehensive evaluation of 86 modification detection tools, Luo et al. (87) demonstrated that some tools originally designed for use with RNA002 data (e.g., m6Anet) could be accurately applied to RNA004 data after retraining with an appropriate RNA004 data set. Although this study was conducted using RNA from human cell lines, it provides a strong proof of concept that some existing tools can successfully be applied to RNA004 data sets after adjustments are made. Future work may well demonstrate the same principle for bacterial RNA, expanding the pool of available tools for the detection of modifications in data produced with RNA004 technology.

## rRNA MODIFICATIONS

**Fig 2:**
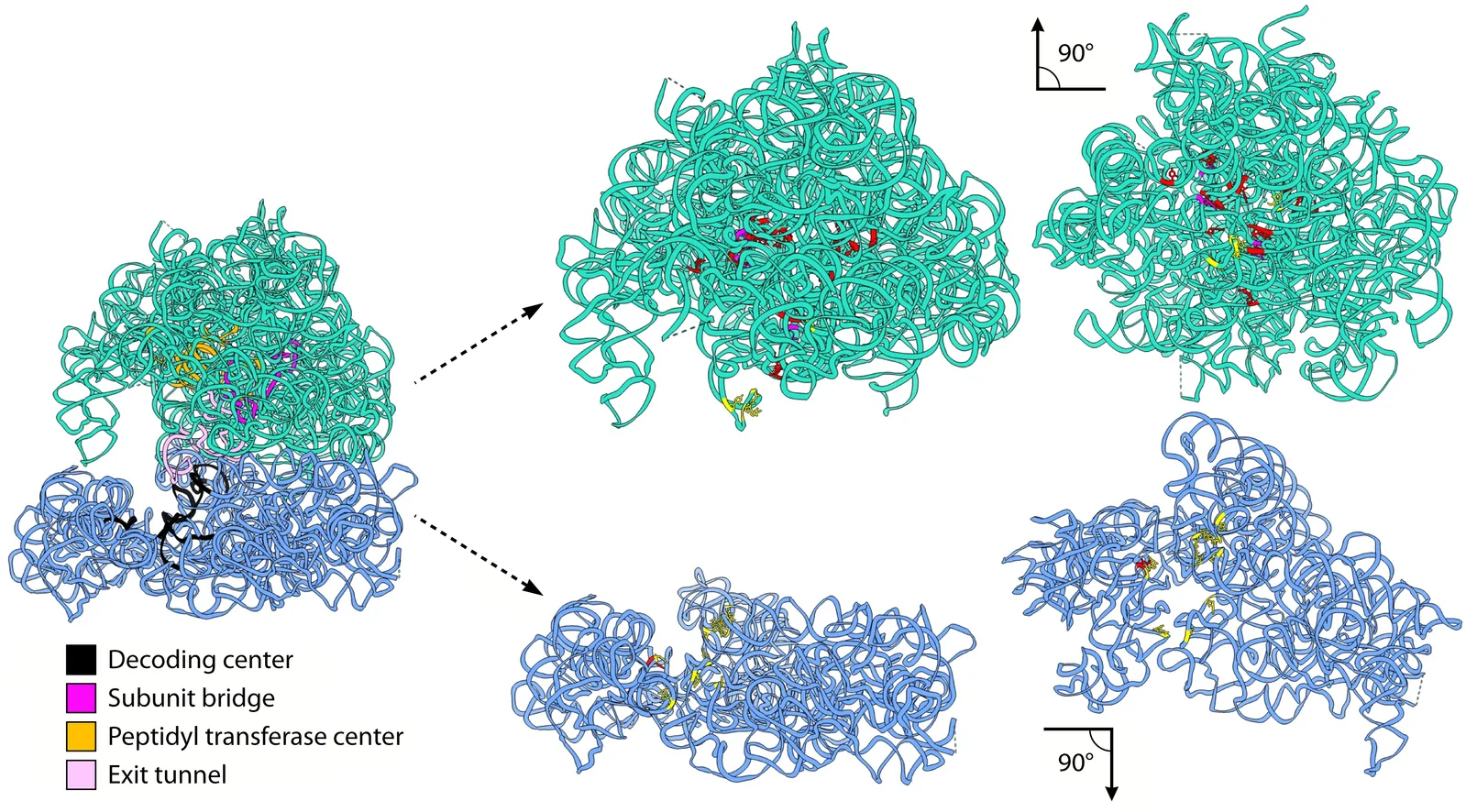
The structure of the *E. coli* rRNA. The 23S rRNA (upper) and 16S rRNA (lower) are shown in two orientations. RNA modification color corresponds to the stage at which it is added: early modifications (red) are added to the naked rRNA, late modifications (yellow) are added to folded rRNA, and intermediate modifications (purple) are either added between early and late modifications or can be added at any stage (see text for details). The structure depicted is based on the Protein Data Bank record pdb_00007k00 (89).

Ribosomes are responsible for decoding the information present in the mRNA to form functional proteins. These structures serve as the platform for key protein synthesis steps: tRNA binding, decoding, and peptidyl transfer. Ribosomes, which are composed of ribosomal RNA (rRNA) and proteins, are one of the most complex and highly conserved machineries in the bacterial cell. In prokaryotes, the ribosome is composed of one large (50S) and one small (30S) subunit. The 50S comprises two sets of rRNAs, the 23S and the 5S rRNAs, together with 34 proteins; the small subunit consists of the 16S rRNA and 21 proteins. The rRNA components of the ribosome are modified post-transcriptionally (88). In *E. coli*, the rRNA contains 36 modification sites, 11 of which are present in the 16S and 25 of which are located in the 23S (2) (Fig. 2). These modifications are required for functional ribosome activity.

rRNA modifications are known to be present from the early stages of ribosome biogenesis, with some modifications aiding in correct rRNA folding (90). Most of the rRNA modifications are clustered around key sites: the decoding center, peptidyl transferase center (PTC), the peptide exit tunnel, and the highly conserved interface between the two subunits, known as the subunit bridge (91–93) (Fig. 2). Although the presence of rRNA modifications has been known for more than 40 years, the exact roles of many remain unclear. However, numerous studies have indicated that specific modifications are essential for ribosome structure and functional regulation.

Under stress, bacteria may use ribosomal heterogeneity (specifically, differences in modification abundance) and functionally specialized ribosomes to fine-tune the translation process (88). An *in vitro* study of *E. coli* showed that a synthetic ribosome lacking 10 different modifications has impaired assembly and reduced functional activity (16). Structurally, rRNA modifications are required to establish three different types of ribosomal contact. The first type is contact with the ribosomal ligands, namely the tRNA and mRNA. The second is contact between the 16S and 23S ribosomal subunits, which is mediated by modifications at the subunit bridge. The third type is intra-subunit interactions. These modifications are located within the interior of the rRNA and generally serve to stabilize the rRNA structure via extension of hydrophobic interactions (94).

**Fig 3:**
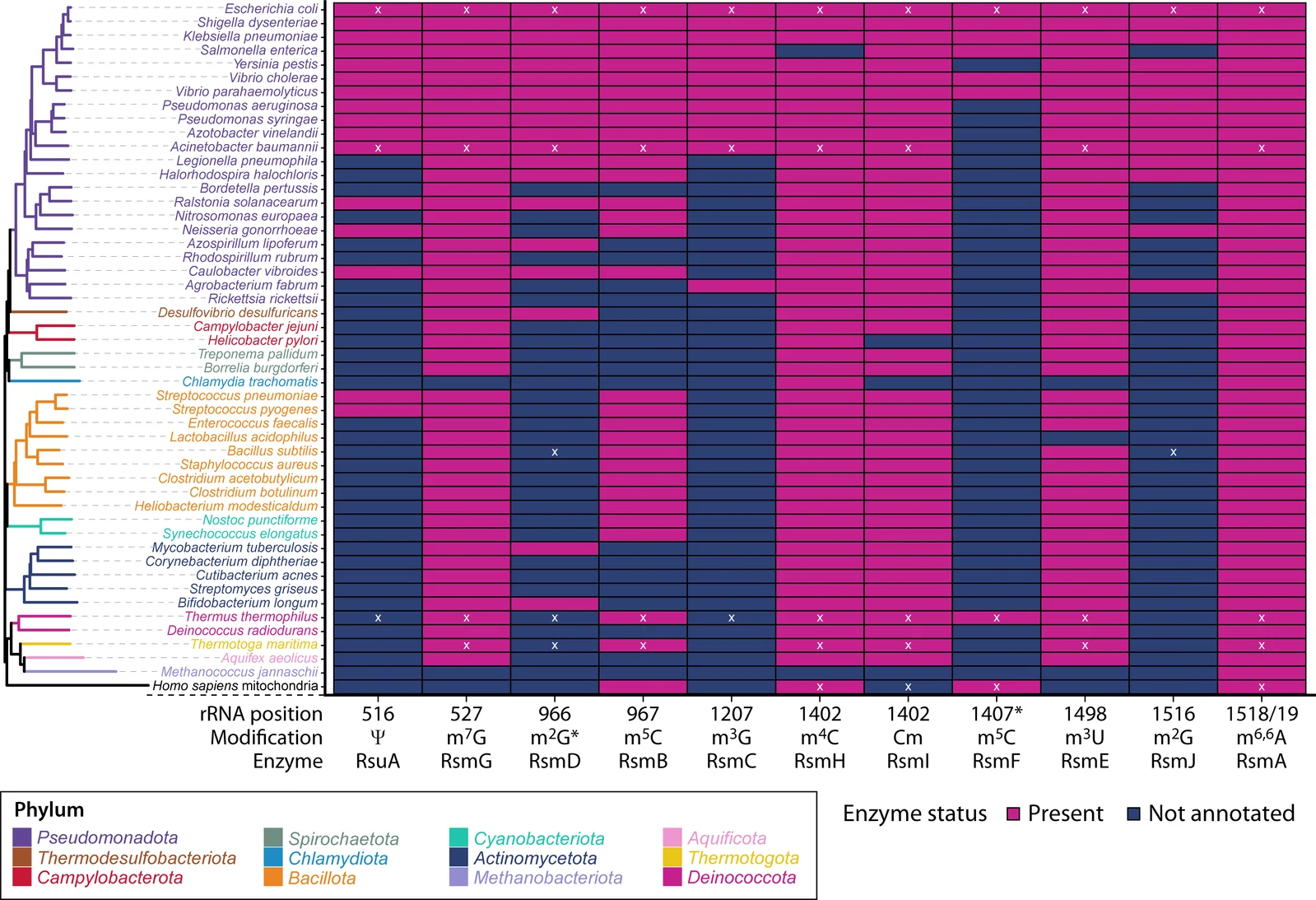
Conservation of modifications and modification enzymes of the *E. coli* 16S rRNA. Enzymes catalyzing each modification type are classified as present or not annotated. X indicates that the presence of a modification at this site has been experimentally confirmed. For comparison, data are shown for one archaeon and human mitochondria. m^2^G*: m^2^G or m^2,2^G. 1407*: 1400, 1404, or 1407.

**Fig 4:**
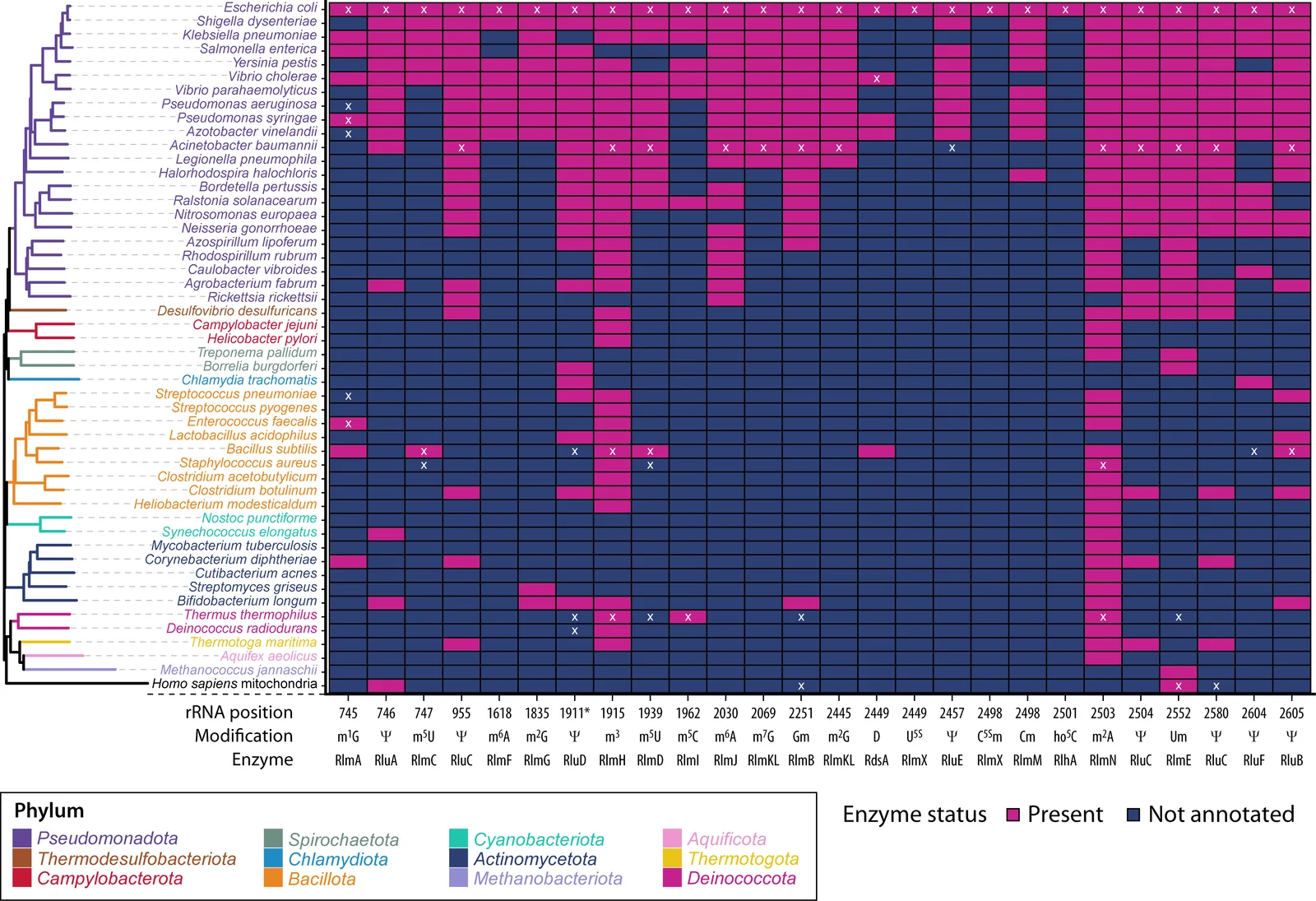
Conservation of modifications and modification enzymes of the *E. coli* 23S rRNA. Enzymes catalyzing each modification type are classified as present or not annotated. X indicates that the presence of a modification at this site has been experimentally confirmed. 1911*: 1911, 1915, and 1917.

In this section, we review knowledge of rRNA modifications, with a focus on *E. coli* as a particularly well-characterized system (Fig. 3 and 4). However, taxon-specific modifications found outside of *E. coli* are also discussed, as are the enzymes responsible for these modifications (both genome- and plasmid-encoded) and the timing of modification addition during ribosome assembly (Table 2). For ease of reference, modifications are divided by rRNA subunit (16S or 23S) and organized by relative position within the rRNA strand from the 5′ to the 3′ end; relative positions refer to those in *E. coli* unless otherwise stated.

**TABLE 2:** Timing of rRNA modification additions^*a*^

Modification	Subunit	Timing	Citation
Ψ516	16S	Intermediate stage	Wrzesinski and Bakin et al. (95)
m^2^G/m^2,2^G966	16S	Late stage	Lesnyak et al.; Burakovsky et al. (96, 97)
m⁵C967	16S	Early stage	Weitzmann et al. (98)
m^2^G1207	16S	Late stage	Tscherne et al. (99)
m^5^C1400	16S	Early stage	Demirci et al. (100)
m^4^Cm1402	16S	Late stage	Kimura and Suzuki (101)
m^5^C1404	16S	Early stage	Demirci et al. (100)
m^5^C1407	16S	Late stage	Andersen and Douthwaite (102)
m^1^A1408	16S	Late stage	Wachino et al. (103)
Cm1409	16S	Prior to full ribosome assembly	Monshupanee et al. (104)
m²G1516	16S	Late stage	Basturea et al. (105)
m^1^G745	23S	Early stage	Siibak and Remme; Gustafsson and Persson (90, 106)
Ψ746	23S	Early stage	Wrzesinski and Nurse et al. (107)
Ψ955, Ψ2504, and Ψ2580	23S	Early stage	Siibak and Remme (90)
Am1067	23S	Prior to complete 50S assembly	Cundliffe and Thompson; Cundliffe (108, 109)
Cm1920	23S	Prior to full ribosome assembly	Monshupanee et al. (104)
m^2^G1835	23S	Early stage	Siibak and Remme (90)
Ψ1911, Ψ1915, Ψ1917	23S	Late stage	Siibak and Remme (90)
m^5^C1942	23S	Early stage	Larsen et al. (110)
m^5^C1962	23S	Early stage	Purta et al.; Sunita et al. (111, 112)
m^6^A2030	23S	Early stage	Golovina et al. (113)
m^7^G2069	23S	Early stage	Siibak and Remme (90)
Ψ2457	23S	Early stage	Siibak and Remme (90)
Cm2498	23S	Intermediate stage	Siibak and Remme (90)
ho^5^C2501	23S	Early stage	Kimura et al. (114)
m^2^A2503	23S	Prior to full large subunit assembly	Stojkovic et al. (115)
Um2552	23S	Late stage	Siibak and Remme; Caldas et al. (90, 116)
Gm2553	23S	Early stage	Roovers et al. (117)
m^7^G2574	23S	Early stage	Wolff et al. (118)
Ψ2604, Ψ2605	23S	Early/intermediate stage	Berk et al; Lin et al. (119, 120)

^*a*^ Modifications are divided by rRNA subunit (16S or 23S) and organized by relative position within the rRNA strand from the 5′ to the 3′ end. Relative positions refer to those in *E. coli*.

### 16S rRNA modifications

#### Pseudouridine (Ψ) at position 516 in 16S rRNA

Position 516 contains the only Ψ identified in the *E. coli* 16S rRNA (121) and is positioned within a region that is fundamentally involved in the fidelity of codon recognition at the ribosomal A-site (121). Its biosynthesis is catalyzed by the synthase RsuA (95, 122). The loss of this modification does not significantly impact bacterial growth rates under standard laboratory conditions, but its presence is hypothesized to fine-tune the structural and functional properties of the decoding center (122).

#### *N*^7^-methylguanosine (m^7^G) at position 527 in 16S rRNA

m^7^G527 is synthesized by the S-adenosyl-methionine (SAM)-dependent methyltransferase RsmG and is a critical component for maintenance of ribosomal decoding accuracy (123). Because the region in which it is located is a primary target for streptomycin binding, loss of this modification due to inactivation of the RsmG active site confers low-level streptomycin resistance in diverse bacterial species (124–127). Specifically, together with residues Pro44 and β-methylthio-Asp88 of the small ribosomal subunit protein uS12, the methyl group of m⁷G527 forms a hydrophobic pocket that directly contacts streptomycin via van der Waals interactions. Loss of the m⁷G527 methyl group weakens these hydrophobic contacts, perturbing the local conformation of the streptomycin-binding site. This reduces drug affinity and contributes to streptomycin resistance (128).

#### *N*^1^-methyladenosine (m^1^A) at position 964 in 16S rRNA of *Streptomyces pactum*

Methylation at site A964 was reported in *S. pactum*. This species produces the antibiotic pactamycin, which binds to a specific portion of the 30S subunit. Examination of a pactamycin-resistant strain revealed that resistance was conferred by the presence of m^1^A964; the methyl group blocks pactamycin binding to the ribosome. m^1^A964 was associated with a novel gene termed *pct* in this particular strain, but the corresponding enzyme was not characterized (129). Thus, the mechanism of m^1^A964 formation is unknown.

#### *N*^2^-methylguanosine (m^2^G)/*N*^2^*N*^2^-dimethylguanosine (m^2,2^G) at position 966 in 16S rRNA

m^2^G966 is introduced by the methyltransferase RsmD, an enzyme that modifies the 2-amino group of guanine 966 (96). In addition, some species bear the hypermodified m^2,2^G966 (130, 131). Situated at the ribosomal P-site, the methyl group of m^2^G966 is essential for stabilizing the early stages of translation initiation (132, 133). Specifically, the methyl group increases the residence time of fMet–tRNA^fMet^ within the 30S pre-initiation complex before recognition of the start codon (97). Structurally, m^2^G966 provides a hydrophobic stacking surface that interacts directly with the anticodon of the P-site tRNA, contacting the ribose of nucleotide 34 (134). Beyond its structural role, m^2^G966 acts as a kinetic checkpoint that regulates mRNA selection and shapes the bacterial proteome (135).

Although m^2^G966 is not essential for core viability under standard laboratory growth conditions, a lack of m^2^G966 has negative impacts. For instance, its absence leads to a delay in translation that results in misregulation of *trp* operon attenuation (135); in this process, ribosome pausing at Trp codons in the *trpL* leader peptide determines whether transcription terminates prematurely or proceeds into the operon. These findings indicate that m^2^G966 can impact translation speed and subsequent expression of downstream genes (136). *E. coli* strains lacking the modification also have decreased fitness under environmental stresses compared to the wild type, demonstrating impaired growth during exposure to low or high temperatures or nutrient limitation (97).

#### N^5^-methylcytidine (m^5^C) at position 967 in 16S rRNA

Deposition of m⁵C in bacterial rRNAs is catalyzed by site-specific SAM-dependent methyltransferases that act at defined stages of ribosome biogenesis, linking modification placement to structural maturation of the ribosome. In *E. coli*, the enzyme RsmB methylates position C967, which is located within the decoding center and participates in translation initiation and codon recognition (99, 102, 137). RsmB methylates unassembled rRNA transcripts early during assembly (98). This indicates that m⁵C installation is coordinated with ribosome assembly checkpoints.

Experimental evidence has linked m⁵C modifications in the 16S rRNA to bacterial fitness and translational performance under stress. For example, the loss of m⁵C967 impairs translation initiation by destabilizing initiator tRNA binding to the 30S pre-initiation complex, resulting in reduced growth efficiency under both rich and minimal growth conditions (97). These findings indicate that m⁵C967 contributes to robust initiation, a process that is particularly sensitive to environmental stress.

#### 2'-*O*-methylcytidine (Cm) at position 1195 in 16S rRNA of *Clostridium acetobutylicum*

Modification of the C at position 1195 to Cm has been documented in *C. acetobutylicum* (130). Position 1195 is located within helix 34 of the 16S rRNA, near the decoding center. Although the surrounding sequences are similar in other bacterial species such as *E. coli*, comparable modifications in other taxa have not been identified (130). Furthermore, the enzyme responsible for this modification has not been described.

#### *N*^2^-methylguanosine (m^2^G) at position 1207 in 16S rRNA

m^2^G1207 is situated within a critical region of the decoding center that is associated with A-site tRNA binding and translocation (138). m^2^G1207 formation is catalyzed by the methyltransferase RsmC (139). The enzyme functions as an RNA chaperone and strand annealer to ensure rapid and correct folding during ribosome biogenesis (140). The functional importance of this site is highlighted by the fact that transversion mutations (G-to-C or -U) at position 1207 severely impact cell growth and ribosome efficiency, resulting in a dominant lethal phenotype (141).

#### *N*^4^,2'-*O*-dimethylcytidine (m^4^Cm) at position 1402 in 16S rRNA

The hypermodified nucleoside m^4^Cm1402 is located within the decoding center, which is a critical component of the bacterial translation machinery due to its contacts with both mRNAs and tRNAs during translation (142, 143). m^4^Cm is regarded as a conserved structural feature of the prokaryotic decoding center; its position places it in close proximity to codon-anticodon interactions at the P-site (101, 144). Biochemical and genetic studies revealed that m^4^Cm formation is catalyzed by two distinct methyltransferases (RsmH and RsmI) and that installation of this modification occurs during the late stages of small subunit maturation (101).

Accumulating evidence indicates that m^4^Cm is not a static structural mark, but rather that it displays sensitivity to environmental conditions. In *E. coli*, cold shock and metabolic stress both lead to selective loss of the *N*^4^-methyl moiety of m^4^Cm1402, whereas the ribose remains methylated (27). This shows that the two methyl groups comprising m^4^Cm can respond differently to stress, yielding ribosomes with altered chemical features in the decoding center. Additionally, m^4^Cm levels increase in *E. coli* in response to heat stress (30). Phenotypic analyses of deletion mutants further support this selective remodeling: under oxidative and acid stress, stress-associated phenotypes are observed primarily in *rsmH* mutants (which lack m^4^C), whereas *rsmI* mutants (lacking Cm) show no detectable abnormal phenotype (31).

m^4^Cm1402 has also been linked to virulence. During silkworm infection, deletion of either *rsmH* or *rsmI* in *Staphylococcus aureus* increases its sensitivity to reactive oxygen species (ROS) and reduces virulence (145). Treatment with antioxidant compounds partially rescues these defects, indicating that m^4^Cm1402 contributes to the protection of the translational machinery under the oxidative stress conditions encountered during host infection.

#### *N*^7^-methylguanosine (m^7^G) at position 1405 in 16S rRNA of *Actinomycetes*

Position 1405 is located within the decoding center of the ribosome. The enzyme responsible for m^7^G1405 catalysis varies between species. In *Micromonospora* spp., methylation is catalyzed by the protein encoded by *grm* (146–148). In *Streptomyces tenebrarius*, m^7^G1405 is formed by the product of *kgmB* (149). The modification can also be catalyzed by ArmA, which was first reported to be present on a plasmid from *Citrobacter freundii* (150) and shows specificity for the 30S subunit (151). Some gram-negative bacteria, including *Pseudomonas aeruginosa* and *Klebsiella pneumoniae*, encode proteins such as RtmA and RtmH that share sequence similarities with ArmA (152–154), but the presence of m^7^G1405 has not been confirmed in these species. Functionally, methylation at position 1405 inhibits interactions between the 30S subunit and aminoglycosides (151), suggesting that m^7^G1405 may have a role in antibiotic stress tolerance.

#### *N*^5^-methylcytidine (m^5^C) at positions 1400 to 1409 in 16S rRNA

The region between 1400 and 1409 is highly modified across numerous bacterial species. Most 16S rRNAs have between two and five m^5^C modifications throughout these positions. Due to the location of these sites within the decoding center, they have direct contact with both tRNAs and mRNAs.

One of the most thoroughly characterized modifications in this region is m^5^C1407, which is catalyzed by RsmF. Much like m^5^C967, m^5^C1407 is located within the decoding center and thus has roles in codon recognition and translation initiation (99, 102, 137). In contrast to RsmB (which catalyzes m⁵C967), RsmF recognizes the structured 30S subunit at later stages of rRNA maturation (102). Structural and kinetic studies have indicated that methylation at this position stabilizes decoding-center conformations that support accurate codon recognition (155).

Several studies have given direct evidence for stress-dependent regulation of m⁵C1407. In *E. coli*, quantitative analyses have demonstrated that m^5^C1407 levels increase during heat, acid, and oxidative stress conditions, partially returning to baseline levels during recovery (65). This dynamic modulation suggests that m⁵C1407 abundance is not constitutively fixed, instead responding to environmental conditions. Together with knowledge of the structural roles of m^5^C1407, these data provide a potential mechanism by which increased m⁵C1407 levels may help preserve translational fidelity under stress (65, 155).

*Enterococcus faecium* bears m^5^C1404, which confers resistance by altering the local chemical environment of the aminoglycoside-binding pocket within the decoding center (156). The modification is added to the 30S ribosomal subunit (but not naked rRNA or the 70S ribosome) by the methyltransferase EfmM (156). Structural analyses show that C1404 lies in immediate proximity to the antibiotic-binding cleft; addition of m^5^C interferes with the hydration network that is required for stable binding of certain aminoglycosides (e.g., kanamycin and tobramycin) (156).

In the thermophile *Thermus thermophilus*, RsmF introduces m⁵C at positions C1400, C1404, and C1407 (100). Although sites C1400 and C1404 can be modified in the naked 16S rRNA, position C1407 can only be modified as part of the complete 30S subunit (100). Deletion of *rsmF* does not abolish growth under optimal conditions, but the mutant displays reduced viability at temperatures below and above the optimum (approximately 60°C and 80°C, compared to the optimal growth temperature of ~70°C) (100). This indicates that multi-site m⁵C modifications contribute to robust ribosomal function during thermal stress.

#### *N*^1^-methyladenosine (m^1^A) at position 1408 in 16S rRNA of *Actinomycetes*

Little is known about the function of m^1^A at position 1408 of the 16S rRNA. However, numerous studies have examined the role and evolution of enzymes catalyzing m^1^A1408. For example, the modification is produced by an enzyme whose origin varies between strains: either a plasmid-encoded Npm enzyme (e.g., NpmA [103], NpmB [157], or NpmC [158]) or a chromosomally encoded enzyme, such as KamA or CmnU. The latter type is uncommon; KamA is found only in *Streptomyces tenjimariensis* (146), and CmnU has been reported only in *Saccharothrix mutabilis* subsp. *capreolus* (159). *npmA* has been identified in both gram-positive and gram-negative species and is likely obtained via horizontal gene transfer. Supporting this hypothesis, the variant *npmA2* has been found only in *Clostridioides difficile* ST11 and some isolates of *E. faecium*, and the gene is associated with transposon Tn*7734* in both cases (160). The presence of m^1^A1408 inhibits interactions between aminoglycosides and the 30S rRNA, conferring aminoglycoside resistance (103).

#### 2′-*O*-methylcytidine (Cm) at position 1409 in 16S rRNA of *Actinomycetes*

In *Mycobacterium tuberculosis*, the positions corresponding to C1409 of the 16S rRNA and C1920 of the 23S rRNA are modified by TlyA (II) (104, 161). Cm1409 is found in the decoding center of the ribosome. The loss of both modifications allows bacteria to resist damage due to capreomycin and viomycin treatment (104, 161). TlyA preferentially modifies the separate ribosomal subunits prior to full ribosome assembly (104). TlyA is also known as a virulence factor; an *M. tuberculosis* mutant lacking the enzyme is more susceptible to autophagy by macrophages (162). BLAST searches indicate that TlyA is also found in other *Actinomycetes* species, such as *Bifidobacterium longum*.

#### *N*^3^-methyluridine (m^3^U) at position 1498 in 16S rRNA

m^3^U1498 is introduced by the methyltransferase RsmE (163). Located at the top of helix 44 of the 16S rRNA within the decoding center and ribosomal P-site, m^3^U1498 is positioned to interact with the mRNA codon region and facilitates inter-subunit communication through interactions with the 23S rRNA (164). Functionally, m^3^U1498 is thought to optimize ribosome activity by altering the local rRNA structure and influencing aminoacyl-tRNA selection and translational fidelity (119, 134).

Although m^3^U1498 is not essential for the growth of *E. coli* in rich or minimal media, the *rsmE* mutant is defective in competition with the wild type (163). Furthermore, a *Mycobacterium smegmatis rsmE* mutant is sensitive to acid stress (165). Notably, the absence of m^3^U1498 is directly associated with low-level resistance to specific aminoglycosides (such as amikacin and kanamycin) that bind in the decoding center proximal to U1498 (165).

#### *N*^2^-methylguanosine (m^2^G) at position 1516 in 16S rRNA

m²G1516 is located in helix 45 of the 16S rRNA near the decoding and mRNA-binding regions. Formation of m^2^G1516 is catalyzed by the methyltransferase RsmJ (105). In *E. coli*, deletion of this enzyme leads to a cold-sensitive phenotype, characterized by impaired growth at lower temperatures (e.g., 16°C) (105). *rsmJ* is overexpressed during adaptive resistance to ampicillin and gentamicin (166).

#### *N*^6^*N*^6^-dimethyladenosine (m^6,6^A) at positions 1518 and 1519 in 16S rRNA

In *E. coli*, m^6,6^A is installed on two adjacent positions in the 16S rRNA by the methyltransferase RsmA (167–169). Functionally, m^6,6^A1518–1519 loss confers resistance to kasugamycin, an antibiotic that targets the decoding region of the 16S rRNA (169). However, *E. coli* cells lacking these modifications are more sensitive to other aminoglycosides (e.g., kanamycin and gentamicin) (170). Recent work in *E. coli* further shows that aminoglycoside treatment promotes the appearance of newly assembled ribosomes lacking m⁶,⁶A, indicating that modification levels can change during antibiotic stress rather than remaining fixed (28). In the same study, transcriptomic analyses revealed that antibiotic exposure induces *de novo* rRNA transcription and ribosome biogenesis pathways, leading to the accumulation of newly synthesized, hypomodified 16S rRNA molecules. DRS experiments confirmed the emergence of a distinct population of low-modification rRNA reads in antibiotic-treated cells (28).

The presence of m^6,6^A1518–1519 is additionally linked with bacterial responses to changes in temperature. In *E. coli*, *rsmA* deletion leads to poor growth and accumulation of immature ribosomal particles at low temperatures (171). This suggests that a lack of m^6,6^A causes defects in late-stage ribosome assembly under cold stress. In contrast, heat stress increases m^6,6^A abundance, suggesting that the decoding center is remodeled to maintain translation at elevated temperatures (30).

The m^6,6^A modifications are also important in pathogens. In *P. aeruginosa*, loss of the *rsmA* homolog *ksgA* reduces resistance to oxidative stress and lowers virulence (170, 172). In *S. aureus*, *ksgA* deletion causes increased oxidant sensitivity, reduced survival in macrophages, and attenuated virulence in a silkworm infection model (173). Transposon disruption of *ksgA* in *Salmonella enterica* serovar Enteritidis reduces intestinal epithelial cell invasion and markedly impairs survival in egg albumen, while simultaneously conferring greater resistance to kasugamycin (174). In *E. coli*, *rsmA* overexpression increases sensitivity to acetate and low pH (175).

#### Pseudouridine (Ψ) at positions 1540 and 1541 in 16S rRNA of *T. thermophilus*

The adjacent positions 1540 and 1541 have been identified as a tandem Ψ site in *T. thermophilus*. These modifications occur at the 3′ tail of the 16S rRNA (176) and are placed within an anti-Shine-Dalgarno mRNA-binding sequence. Although no studies have yet addressed the impacts of Ψ1540–1541 presence, it is hypothesized to stabilize rRNA−mRNA binding in thermophilic environments, similar to the role of tandem Ψ in the tRNA (131).

### 23S rRNA modifications

#### *N*^1^-methylguanosine (m^1^G) at position 745 in 23S rRNA

Formation of m^1^G at position 745 (gram-negative bacteria) or 748 (gram-positive bacteria) is catalyzed by RlmA (90, 106). An *E. coli* mutant lacking this modification shows a reduced growth rate, lower translation efficiency, and decreased abundance of loosely coupled ribosomes (106). The latter result demonstrates the importance of m^1^G745 in ribosome assembly. In gram-positive bacteria, methylation of G748 in conjunction with another modification (m^6^A2058) confers resistance to macrolide antibiotics (177, 178). In contrast, the presence of m^1^G748 in *Streptococcus pneumoniae* enhances interactions between the ribosome and the ketolide antibiotic telithromycin, with these interactions promoting antibiotic susceptibility (179). Impacts of m^1^G745 on antimicrobial sensitivity thus vary between antibiotic classes.

#### Pseudouridine (Ψ) at position 746 in 23S rRNA

Pseudouridylation of U746 is catalyzed by RluA, which is also responsible for modifications in the tRNA and mRNA (107, 180). In competition assays, a Δ*rluA E. coli* mutant shows a slight growth defect compared to the parental strain (181). This may indicate that the presence of Ψ746 (together with the tRNA modifications catalyzed by the same enzyme) is necessary for optimal fitness.

#### *N*^5^-methyluridine (m^5^U) at position 747 in 23S rRNA

m^5^U formation is catalyzed by RlmC in gram-negative bacteria (182); gram-positive bacteria instead encode the dual-functional enzyme RlmCD, which is responsible for methylation at both U747 and U1939 of the 23S rRNA (183, 184). In *S. pneumoniae,* U747 methylation promotes formation of m^1^G at the adjacent site 748, increasing sensitivity to telithromycin. RlmC possesses an FeS cluster, suggesting possible regulation of the enzymatic activity (and thus m^5^U747) by oxidative stress (185).

#### Pseudouridine (Ψ) at positions 955, 2504, and 2580 in 23S rRNA

Ψ955 is located in domain II of the 23S rRNA (186), whereas Ψ2504 and Ψ2580 are located in the PTC in domain V (187). All three modifications are catalyzed by the Ψ synthetase RluC (188). This multi-site enzyme functionality has hindered efforts to determine the effects of each modification individually. For this reason, the three modifications are here discussed together.

Although *rluC* genes are found in various *γ-proteobacteria*, they are not universally conserved (187) (Fig. 4), suggesting that Ψ955, Ψ2504, and Ψ2580 are non-essential for protein synthesis. Furthermore, there is no evidence of a link between RluC activity and ribosomal function under standard or slightly stressful growth conditions; *rluC* deletion reportedly has no notable effect on bacterial growth in either rich or minimal medium or across a range of temperatures (24°C–42°C) (188). Thus, *rluC* knockouts are relatively resilient to nutrient and heat stress.

The primary functional consequence of RluC-mediated rRNA modification appears to be modulation of antibiotic sensitivity. The presence of Ψ2504 acts as an intrinsic resistance mechanism that protects against protein synthesis inhibitors that target the PTC (187). Cells lacking RluC show increased susceptibility to antibiotics that bind to the PTC (such as tiamulin, linezolid, and clindamycin), but not to streptomycin (187).

There is some evidence that Ψ955, Ψ2504, and Ψ2580 are associated with the cold stress response via interactions with the GTPase BipA in *E. coli*. Concurrent *rluC* deletion suppresses many abnormal phenotypes of *bipA* mutants, including cold-sensitive growth at 20°C, decreased capsule synthesis, and defective 50S subunit assembly (189). Wild-type ribosomes require BipA for efficient assembly and translation under cold stress, but ribosomes without the three Ψ modifications are BipA-independent (190). This suppression phenotype is specific to *rluC*; deletion of another Ψ synthase gene (namely *rluA*, *rluB*, *rluE*, or *rluF*) does not alleviate BipA-dependent cold sensitivity, demonstrating the functional importance of Ψ955, Ψ2504, and/or Ψ2580 (189).

#### 2'-*O*-methyladenosine (Am) at position 1067 in 23S rRNA of *Actinomycetes*

*Streptomyces azureus* bears an Am at position 1067, and this modification is formed by the enzyme thiostrepton-resistance methyltransferase (Tsr) prior to complete assembly of the 50S subunit (108, 109). As the name of the enzyme suggests, methylation of site A1067 by Tsr prevents thiostrepton activity, conferring resistance on strains bearing the modification (191). Thiostrepton binds to a 58-nucleotide domain of the 23S rRNA that typically interacts with the ribosomal protein L11, meaning that loss or mutation of L11 can also confer thiostrepton resistance through a mechanism similar to that of Am1067 addition (192, 193). A BLAST search reveals that Tsr is also found in other *Actinomycetes* species, such as *M. tuberculosis* and *Corynebacterium diphtheriae*.

#### *N*^6^-methyladenosine (m^6^A) at position 1618 in 23S rRNA

The most thoroughly characterized RNA modification in eukaryotes is m^6^A, and this base of knowledge has translated to a great deal of research interest in prokaryotic m^6^A. In bacterial rRNAs, m^6^A serves as a molecular “glue”, stabilizing long-range tertiary interactions and mediating ribosomal active site interactions with the nascent peptide (194). Site A1618 is methylated by the methyltransferase RlmF (195). Knocking out this enzyme causes moderate growth defects (195), demonstrating the importance of m^6^A1618 in translation.

In *E. coli*, m^6^A depletion at both positions 1618 and 2030 reduces the infection potential of T5 bacteriophage (196). A Δ*rlmF* Δ*rlmJ* double mutant (which lacks m^6^A1618 and m^6^A2030) is less susceptible to T5 infection than the parental strain, suggesting that host rRNA methylation is exploited by certain phages for optimal replication (196). This defensive advantage is phage-specific but demonstrates the fact that m^6^A in the rRNA can influence the success of lytic infection.

#### *N*^2^-methylguanosine (m^2^G) at position 1835 in 23S rRNA

The G residue at position 1835 is modified to m^2^G by the methyltransferase RlmG (197) in members of the phyla *Pseudomonadota* and *Actinomycetota* (Fig. 4). m^2^G1835 is one of the first modifications to appear during ribosome assembly (90) (Fig. 2), suggesting that it is important for proper rRNA folding or that the enzyme cannot access site 1835 in mature rRNAs. Δ*rlmG* mutants show reduced fitness in competition assays with the parental strain; this effect is more pronounced at high temperatures and in minimal media (197), indicating a possible role of m^2^G1835 in stress responses.

#### Pseudouridine (Ψ) at positions 1911 and 1917 and *N*^3^-methylpseudouridine (m^3^Ψ) at position 1915 in 23S rRNA

Three Ψ modifications at the neighboring positions 1911, 1915, and 1917 are catalyzed by RluD in *E. coli*. Ψ1915 additionally undergoes methylation by RlmH at the *N*^3^ position, forming m^3^Ψ (198, 199). In contrast to these well-defined modifications, position 1915 is known to be methylated in *T. thermophilus*, but the identity of the base as Ψ or U cannot be distinguished (200).

In *E. coli*, an absence of RlmH leads to reduced fitness in competition assays with the wild-type (198), demonstrating the importance of the methyl group at position 1915 specifically. Furthermore, *rluD* mutants have severe growth defects (201) or are not viable (202), likely due to the importance of Ψ1911, m^3^Ψ1915, and Ψ1917 in ribosome assembly (203). The abnormal phenotype may also be related to the function of Ψ in translation termination; a lack of either *rluD* or *prfB* (the latter of which encodes the translation termination factor RF2) produces stop codon misreading (204). In *E. coli*, the defective growth of Δ*rluD* mutants can be mitigated by an additional mutation in *prfB* leading to a Glu172→Lys replacement (204). Conversely, a lack of *rluD* in *Salmonella* spp. causes growth suppression only in strains with an Ala246Thr mutation in RF2, whereas Δ*rluD* strains having Ala246 (e.g., *S. enterica*) show adverse growth effects (205, 206).

#### 2′-*O*-methylcytidine (Cm) at position 1920 in 23S rRNA of *Actinomycetes* and *Campylobacteria*

As noted in the 16S rRNA section “Cm1409” above, formation of Cm at both the 16S rRNA position 1409 and the 23S rRNA position 1920 is catalyzed by TlyA (II) in species such as *M. tuberculosis*. However, other species (including *Campylobacter jejuni*, *Helicobacter pylori*, and *Brachyspira hyodysenteriae*) encode the ortholog TlyA (I), which is specific for position 1920 (207). In addition to its methyltransferase activity, purified TlyA (I) can have hemolytic activity *in vitro* (e.g., TlyA from *Brachyspira hampsonii*), which appears to be dependent on disulfide bridge bonds (208). Despite this additional TlyA functionality, abnormal phenotypes in *C. jejuni tlyA* mutants are due specifically to the methylation activity; phenotypes related to motility, biofilm formation, and adhesion to and invasion of human epithelial cells are consistent between *tlyA* knockouts and those with point mutations affecting only the methyltransferase domain (207, 208). Complementation with the native TlyA (I) restores virulence, whereas complementation with the mycobacterial TlyA (II) gene enhances virulence (207).

#### *N*^5^-methyluridine (m^5^U) at position 1939 in 23S rRNA

m^5^U at position 1939 is catalyzed by RlmD (182, 209), as demonstrated *in vitro* (209). RlmD contains a [4Fe-4S] cluster, which is atypical for a methyltransferase. Fe-S cluster proteins are generally sensitive to oxidative stress, suggesting a possible mechanism of oxidative-stress regulation for m^5^U1939 (209). However, studies showing a direct effect of oxidative stress or any other stress condition in relation to m^5^U1939 have not been reported. The role of m^5^U1939 is therefore unknown at this time.

#### *N*^5^-methylcytidine (m^5^C) at position 1942 in 23S rRNA of *Deinococci*

Position 1942 bears an m^5^C modification in *T. thermophilus* and *D. radiodurans* (110). m^5^C formation is catalyzed by RlmO in the naked 23S rRNA, but not assembled 70S ribosomes (110), and the modification must therefore be added early during the ribosome maturation process. RlmO homologs have been found in members of the genus *Deinococcus* and the class *Fusobacteriota*, although the presence of the modification itself has not been confirmed outside of *T. thermophilus* and *D. radiodurans* (110). In contrast to m^5^C modifications formed in the 16S rRNA of *T. thermophilus* by RsmF (at positions C1400, C1404, and C1407), RlmO activity does not appear to be related to thermal adaptation (110).

#### *N*^5^-methylcytidine (m^5^C) at position 1962 in 23S rRNA

Formation of m^5^C1962 is catalyzed by the methyltransferase RlmI, a homodimer that recognizes its target sequence in the naked 23S rRNA prior to 50S subunit assembly (111, 112). Although the absence of m^5^C1962 causes only a marginal growth reduction under optimal growth conditions, the modification is known to contribute to bacterial fitness and stress responses in *E. coli*. For example, RlmI-deficient strains show reduced biofilm formation and decreased fitness under sub-inhibitory concentrations of aminoglycoside and fluoroquinolone antibiotics (111, 210). Furthermore, RlmI activity is dynamically regulated by the second messenger molecule cyclic di-guanosine monophosphate (c-di-GMP), binding of which inhibits RlmI. This inhibition modulates ribosome assembly, promoting tolerance of otherwise lethal concentrations of kanamycin and ampicillin (211).

#### *N*^6^-methyladenosine (m^6^A) at position 2030 in 23S rRNA

Formation of m^6^A at position 2030 is catalyzed by RlmJ (113) early during ribosome biogenesis (Fig. 2). RlmJ is conserved throughout the phylum *Pseudomonadota* (Fig. 4) (212), but evidence of the modification itself is scarce. Structurally, position 2030 is found within the exit tunnel. In contrast to Δ*rlmF* mutants (which lack m^6^A at position 1618), Δ*rlmJ* mutants have a slight growth advantage under anaerobic conditions (113). These data suggest that m^6^A2030 is not as functionally critical under stress conditions as other rRNA modifications are. However, DRS data have shown that cold shock and metabolic stress lead to a significant decrease in m^6^A2030 (27). The loss of this modification helps to reduce translational efficiency, enabling resource redirection toward stress tolerance mechanisms. Additionally, loss of this modification together with the loss of m^6^A1618 in an *E. coli* Δ*rlmF* Δ*rlmJ* double mutant reduces the infectious potential of T5 bacteriophage (196).

#### *N*^6^-methyladenosine (m^6^A)/*N*^6^*N*^6^-dimethyladenosine (m^6,6^A) at position 2058 in 23S rRNA

Position 2058 can be modified to either m^6^A or m^6,6^A by members of the Erm rRNA methyltransferase superfamily (213, 214). *erm*-family genes are widely distributed among hospital-acquired (nosocomial) strains of both gram-positive and gram-negative bacteria (215) (Fig. 4), and modification at this site confers resistance to macrolide, lincosamide, and streptogramin B (collectively referred to as MLS) antibiotics (216, 217). In *S. aureus*, the presence of m^6^A2058 alters interactions between nascent peptides and the ribosome tunnel, reducing translation efficiency and fidelity (218). This occurs via downregulation of major virulence genes. Modifications at this position also promote bacterial evasion of the innate immune response by affecting toll-like receptor recognition (219).

#### *N*^7^-methylguanosine (m^7^G) at position 2069 in 23S rRNA

In *E. coli*, m^7^G formation at position 2069 is catalyzed by the dual-functional methyltransferase RlmKL (220). This modification has also been identified in *Acinetobacter baumannii* (2), but is absent in gram-positive bacteria (221). Δ*rlmKL* mutants, which are unmethylated at both G2445 and G2069, have a delayed growth phenotype under standard culture conditions and show increased susceptibility to ROS damage after UVA exposure (222). This may be due to a lack of interactions with stabilizing ribosomal proteins.

#### 2′-*O*-methylguanosine (Gm) at position 2251 in 23S rRNA

Gm2251 formation is catalyzed by RlmB (223). Structurally, this modification is in contact with the 3′ end of tRNAs within the P-site (200). Similar to Δ*rlmKL* mutants (which lack m^7^G2069 and m^2^G2445), a Δ*rlmB E. coli* mutant was shown to experience high levels of ROS damage after UVA exposure in comparison to the parental strain (222). However, another study showed that the Δ*rlmB* mutant has no abnormalities in ribosome assembly and no reduction in fitness, even after 120 generations (223). This indicates that Gm2251 is not an essential modification under standard growth conditions, but rather may function in stress responses.

#### *N*^2^-methylguanosine (m^2^G) at position 2445 in 23S rRNA

Like m^7^G2069, formation of m^2^G at position 2445 is catalyzed by RlmKL in *E. coli*, a process that is reliant on SAM (220). This modification is located in a helix next to the PTC (221), suggesting a potential role in ribosomal structure. Based on predicted binding structures of ribosomes with modified and unmodified nucleotides (224), it has been suggested that the modification optimizes ribosome conformation; this is also based on the observation that the *rlmKL* knockout mutant has delayed but not halted growth (224). As noted above, the Δ*rlmKL* mutant exhibits increased susceptibility to ROS damage as a result of UVA exposure (222), indicating the importance of m^2^G2445 and/or m^7^G2069 in the bacterial stress response.

#### Dihydrouridine (D)/5′(S)-methyl dihydrouridine (D^5S^m) at position 2449 in 23S rRNA

In *E. coli*, position U2449 is modified to D by RdsA (66), forming a modification that may be broadly conserved among bacteria (186). The resulting base can be further modified at the 5′(S) carbon of the ribose moiety to generate D^5S^m, which is catalyzed by the recently discovered cobalamin-dependent radical SAM methyltransferase RlmX (225). Like many 23S modifications, D/D^5S^m2449 is located within the PTC.

Mutants lacking D2449 show normal growth under standard culture conditions and do not exhibit increased antibiotic sensitivity compared to the parental strain (226). However, D^5S^m2449 levels increase during anaerobic growth (225), likely because the [4Fe-4S] cluster in RlmX is more stable in the absence of oxygen. It is hypothesized that D^5S^m2449 increases PTC and P-site stability. In contrast, iron starvation results in increased D2449 levels compared to D^5S^m2449 abundance (225). These findings demonstrate strong stress responsiveness of the modifications at site 2449.

#### Pseudouridine (Ψ) at position 2457 in 23S rRNA

Pseudouridylation at position 2457 was first discovered in *E. coli* (50) but has also been confirmed in *A. baumannii* (2). The modification may be broadly conserved among bacteria (186). Ψ2457 is installed in the early stages of ribosome assembly (90) (Fig. 2) by RluE (227), which functions only at position 2457. The specificity of RluE for this site is due to recognition of rRNA helix 89 and sequence-specific interactions between the rRNA and loops in the enzyme (228). Deletion mutants show normal growth under standard growth conditions and under high or low temperatures (227), indicating that this modification is not essential under these conditions.

#### *N*^1^-methylguanosine (m^1^G) at position 2470 in 23S rRNA of *Bacilli*

*E. faecium* is methylated at position G2470 by evernimicin methyltransferase (EmtA). The enzyme is plasmid-encoded (229), suggesting that other species may also bear this modification, although this has yet to be confirmed. m^1^G2470 inhibits antibiotic interactions with the ribosome, conferring high-level antibiotic resistance.

#### 2′-*O*-methyluridine (Um) at position 2479 in 23S rRNA of *Actinomycetes*

*Streptomyces viridochromogenes* has been found to have a Um modification at position 2479. Formation of Um2479 is catalyzed by the methyltransferase AviRb (230). *S. viridochromogenes* produces the antibiotic avilamycin (231, 232), which binds to helix 89 (near the A-site) of the large ribosomal subunit in gram-positive bacteria (233). However, the presence of Um2479 prevents binding, conferring avilamycin resistance to strains with this modification (234).

#### 2′-*O*-methylcytidine (Cm) at position 2498 in 23S rRNA

Methylation at position C2498 of the 23S rRNA is catalyzed by RlmM, which is located in the PTC (235). Although Cm2498 has been identified as a basal modification in *E. coli*, recent evidence demonstrates that it functions as a scaffold for additional stress-induced backbone modifications (225). For example, under anaerobic conditions, position 2498 becomes hypermethylated, forming 5′(S)−2′-*O*-dimethylcytidine (Cm^5S^m) in a reaction catalyzed by RlmX (225). Levels of both Cm and Cm^5S^m at position 2498 increase significantly under anaerobic growth conditions, indicating that these modifications are some of the most important in bacterial stress responses.

*E. coli* cells lacking Cm2498 have slightly lower fitness than the wild type (235). This may be due to the significant decrease in peptide transfer rates and total protein synthesis observed in mutants lacking Cm2498 (225). Furthermore, strains without functional RlmX have decreased sensitivity to erythromycin and clindamycin, indicating that hypermethylation at position 2498 impacts access to the antibiotic binding pocket in the PTC (225).

#### 5-hydroxycytidine (ho^5^C) at position 2501 in 23S rRNA

ho^5^C2501 is a unique modification in the PTC; it is the only hydroxylation-based modification in the rRNA. The modification is installed by RlhA, a FeS cluster-containing protein that requires the hydroxyl group donor prephenate for catalytic activity (114). The enzyme recognizes the early-stage assembly intermediate of the 50S ribosomal subunit, hydroxylating it prior to ribosomal maturation.

The location of this modification plays an important role in its functionality. Its position within the PTC indicates that it may influence translation via recognition of P-site tRNAs through interactions with tRNA position A76 specifically (114). Furthermore, hydroxylation increases interactions of C2501 with neighboring residues and promotes stacking between G2446 and G2447, fixing C2501 in a defined position (236). Hydroxylation also promotes interaction of C2501 with the inner core PTC residues (namely C2063 and A2451) (114). These data suggest that ho^5^C2501 is integral to both proper ribosome structure and correct tRNA recognition.

ho^5^C2501 levels are dependent on growth phase; 10%–30% of rRNAs bear this modification during exponential growth, a proportion that can increase to 80% in stationary phase (236). In *E. coli*, loss of ho^5^C as a result of knocking out *rlhA* causes only minor growth defects under optimal culture conditions. However, Δ*rlmX* Δ*rlhA* double knockout mutants (lacking not only ho^5^C2501 but also D^5S^m2449 and Cm^5S^m2498) show significantly slower growth rates than the wild type under anaerobic conditions (225).

ho^5^C2501 abundance can also be altered by stress exposure. For example, anaerobic conditions increase levels of ho^5^C2501, which are hypothesized to affect translation by contributing to PTC stabilization, restricting flexibility (225). RlhA requires iron to function, meaning that ho^5^C2501 is also metabolically modulated by environmental iron availability: iron depletion using chelators (e.g., 2,2′-dipyridyl) causes a marked reduction in hydroxylation frequency at position 2501 (114). High levels of ho^5^C2501 are beneficial for surviving strong oxidative stress. Increased abundance of the modification is associated with upregulation of Dps, a protein induced by starvation and ROS that protects bacteria from various stresses. Δ*rlhA* knockouts fail to produce Dps, leading to irreparable DNA damage during ROS exposure (236). These findings highlight the importance of ho^5^C2501 in the bacterial stress response.

#### 2-methyladenosine (m^2^A), 8-methyladenosine (m^8^A), and 2,8-dimethyladenosine (m^2,8^A) at position 2503 in 23S rRNA

m^2^A2503 is a highly conserved post-transcriptional modification present in the 23S rRNA of both gram-positive and gram-negative bacteria (Fig. 4). It is catalyzed by the methyltransferase RlmN, a radical SAM enzyme with dual substrate specificity that functions on the tRNA in addition to rRNA (237, 238). m^2^A is incorporated at position 2503 during the narrow timeframe before the large subunit is fully assembled, when free 23S rRNA is available (115). The functional significance of this modification arises from its unique placement within the PTC: position 2503 is located at the entrance of the nascent peptide exit tunnel (239) and contacts the newly made peptide during the process of translation (240). It therefore functions as a translation quality checkpoint.

Generally, mutants without m^2^A2503 exhibit reduced translational and proofreading accuracy (238). Such mutants additionally experience translation stalling due to a lack of interactions between A2503 and A2062, the nascent peptide sensor in the ribosome exit tunnel (240). In *E. coli*, *rlmN* knockouts show a two-fold increase in tiamulin and sparsomycin susceptibility (237). The opposite effect is observed in *S. aureus*, in which *rlmN* knockouts (but not the wild type) survive under the selective pressure of linezolid treatment (237). This is consistent with clinical studies demonstrating that Δ*rlmN* cells dominate *S. aureus* populations during prolonged linezolid therapy (241). The function of m^2^A2503, therefore, appears to differ between species.

In *Enterococcus faecalis*, m^2^A2503 is an environmentally responsive modification that varies in abundance with conditions such as ROS exposure (242). For example, there are significant decreases in m^2^A2503 levels after exposure to the superoxide generator menadione or to the ROS-inducing bacteriostatic antibiotics erythromycin and chloramphenicol. Furthermore, the proteome of the *rlmN* knockout mutant mimics that of cells grown under sustained oxidative stress; this includes upregulation of superoxide dismutase (SodA) and downregulation of virulence factors like the pilus subunit EbpA (242). The ROS sensitivity of RlmN is hypothesized to be the link between ROS exposure and decreased m^2^A2503. Indeed, although ROS treatment does not impact RlmN abundance, it directly inactivates the Fe-S cluster required for enzyme function (242).

Position A2503 is also the target site for another methyltransferase, Cfr, which is encoded on *Staphylococcus* multidrug resistance plasmids (243–245). Cfr modifies A2503 to m^8^A2503 (or m^2,8^A2503 in the absence of *rlmN*), with the 8-methyl moiety of the modification contributing to antibiotic resistance (246). Specifically, the 8-methyl moiety interferes with associations between antibiotic molecules and the PTC (247), rendering bacteria resistant to phenicols, lincosamides, oxazolidinones, pleuromutilins, and streptogramin A antibiotics (247). Heterologous *cfr* expression confers resistance in both gram-positive and gram-negative bacteria (247). Thus, modifications at position A2503 have important functions in translation quality control, bacterial stress responses, and antibiotic resistance.

Notably, m^2^A modifications are specific to the domain *Bacteria* and are found not only in model laboratory strains such as *E. coli* str. K-12 substr. MG1655 but also in clinically relevant pathogenic species, including *M. tuberculosis* (248). Under standard growth conditions, bacterial cells do not release modified nucleotides; however, under lytic stress due to antibiotic exposure or immune cell phagocytosis, m^2^A can be detected in the extracellular fluid. For example, one clinical study found elevated levels of m^2^A in the urine of patients with bacterial infections (249), demonstrating that m^2^A can be used as a bacterial infection biomarker.

#### *N*^1^-methylguanosine (m^1^G) at position 2535 in 23S rRNA of *Actinomycetes*

*S. viridochromogenes* bears a methyl group at position G2535 of the 23S rRNA, forming m^1^G2535 (230, 250). This modification is catalyzed by the methyltransferase AviRa (230). The presence of m^1^G2535 confers resistance to avilamycin, which is produced by *S. viridiochromogenes* itself (232). This is similar to the mechanism of avilamycin resistance conferred by AviRb (234) as discussed above (Um2479).

#### 2′-*O*-methyluridine (Um) at position 2552 in 23S rRNA

Um2552 is installed by the SAM-dependent methyltransferase RlmE (also known as RrmJ). This enzyme has activity on the assembled 50S subunits and the 70S ribosome, but not on protein-free 23S rRNA, suggesting that it requires the pre-formed ribonucleoprotein architecture to function (116, 251). Um2552 thus serves as a late-stage checkpoint during large subunit maturation (90, 116) (Fig. 2).

In *E. coli*, *rlmE* mutants exhibit a 2-fold decrease in growth rate compared to the parental strain at temperatures ranging from 10°C to 42°C (116). A lack of Um2552 has been found to increase translational accuracy (252). However, the absence of the modification also decreases 70S stability, causing dissociation into separate subunits (116).

Um2552 is associated with bacterial stress responses, particularly the heat shock response (253). RlmE is a heat-induced protein, and *rlmE* is the first cistron of the *rrmJ-hflB (ftsH*) heat shock operon. An *E. coli* Δ*rlmE* mutant displays a cold-sensitive phenotype and accumulation of 45S ribosomal assembly precursors (254). This indicates that RlmE plays an important role in proper folding and assembly of the 50S when environmental conditions are not favorable (e.g., during low temperature exposure, when ribosome assembly is energetically difficult) (254).

#### 2′-*O*-methylguanosine (Gm) at position 2553 in 23S rRNA of *Bacillus*

In *B. subtilis*, position 2553 (in stem-loop h-92 of the 23S) is O-methylated to form Gm (255, 256). This modification is catalyzed by the enzyme RlmP; *in vitro* data indicate that the process occurs early during ribosome assembly (117). A mutant lacking the gene encoding RlmP (Δ*ysgA*) shows no changes in growth compared to the wild type in either rich or minimal media, and no differences have been observed in the ribosome profile at 37°C or 22°C (117).

#### *N*^7^-methylguanosine (m^7^G) at position 2574 in 23S rRNA of *B. subtilis*

Like Gm2553, m^7^G2574 is located within the stem-loop h90 region of the *B. subtilis* 23S rRNA (255, 256). RlmQ, encoded by *ywbD*, most efficiently installs the modification on the naked RNA (118). Mutants without RlmQ have no documented abnormal phenotypes with respect to growth, ribosome profiles, or antibiotic sensitivity compared to the wild type (118).

#### Pseudouridine (Ψ) at positions 2604 and 2605 of 23S rRNA

Ψ2604 and Ψ2605 are post-transcriptional modifications present in the PTC (227, 257). Ψ2604 formation is catalyzed by RluF, a member of the RsuA Ψ synthase superfamily (258). RluF is a dual-specificity enzyme that also modifies position 35 in the anticodon of tRNA^Tyr^ (259). Notably, several bacterial species encode RluF homologs but lack the Ψ2604 modification (259). Ψ2605 is catalyzed by another member of the RsuA Ψ synthase superfamily, RluB (257). The addition of these modifications early during ribosome assembly ensures that the PTC is functionally primed.

The presence of Ψ2604 and Ψ2605 in the PTC suggests that these modifications could play significant roles in translation mechanics, but this hypothesis has not been functionally validated. As positions located in the heart of the translation machinery, Ψ2604 and Ψ2605 may further serve as stabilizers to fine-tune ribosome integrity under stress conditions. However, a direct link between Ψ2604–Ψ2605 and the bacterial stress response has yet to be established. For example, *rluF* deletion has no effect on the exponential growth rate of *E. coli* at temperatures ranging from 25°C to 42°C (227).

### rRNA modification conservation

rRNA modifications have been studied comprehensively in only a few bacterial species. For example, as mentioned above, it is known that *E. coli* has 36 rRNA modification sites, with 11 in the 16S and 25 in the 23S. The gram-positive model organism *B. subtilis* has fewer known rRNA modification sites at 25 in total, nine of which are in the 16S rRNA and 15 in the 23S rRNA (256). *T. thermophilus* has even fewer, with 12 modifications in the 16S and 11 in the 23S (94, 131, 200).

To assess the prevalence of rRNA modifications and the associated enzymes throughout the domain *Bacteria* more broadly (Fig. 3 and 4), we used modification data from MODOMICS (2), proteome annotations from UniProt (260), and deep literature searches. Analyses were conducted for a representative selection of 48 bacterial species, in addition to one archaeon and the human mitochondria for comparison. Species were selected for this analysis to represent a broad swathe of the bacterial tree of life, with preference given to those with biological importance (either for human health, the environment, or scientific research) and those for which modification-related data are available (Table 3). Many species have not yet been analyzed for the presence of rRNA modifications; furthermore, there are well-documented limitations of protein functional annotation approaches that are based primarily on sequence similarity, which often overlook relevant enzymes (261). The absence of a modification or the associated enzyme in this analysis may therefore not represent a definitive lack biologically, but rather the absence of available data. Nevertheless, this is a comprehensive snapshot of current knowledge regarding the evolutionary distribution of rRNA modifications.

**TABLE 3:** Taxa included in modification conservation analyses^*a*^

Taxon	Rationale	UniProt proteome no.
*Acinetobacter baumannii* str. ATCC 19606	rRNA modification data available	UP000498640
*Agrobacterium fabrum* str. C58	Causes crown gall tumor in plants; used for plant transformations	UP000000813
*Aquifex aeolicus* str. VF5	Thermophile found in deep-sea vents	UP000000798
*Azospirillum lipoferum* str. 59b	Plant growth promoter	UP000324927
*Azotobacter vinelandii* DJ	Free-living, nitrogen-fixing bacterium found in soil	UP000002424
*Bacillus subtilis* subsp. subtilis str. 168	Common soil bacterium	UP000001570
*Bifidobacterium longum* subsp. infantis	Common gut bacterium in humans	UP000043107
*Bordetella pertussis* Tohama I	Causative agent of whooping cough in humans	UP000002676
*Borrelia burgdorferi* B31	Causative agent of Lyme disease in humans	UP000001807
*Campylobacter jejuni* subsp. jejuni str. NCTC 11168	Causative agent of gastroenteritis in humans	UP000000799
*Caulobacter vibroides* NA1000	Common in freshwater lakes and streams	UP000001364
*Chlamydia trachomatis* D/UW-3/CX	Causative agent of chlamydia in humans	UP000000431
*Clostridium acetobutylicum* DSM 1731	Biotechnologically relevant solventogenic *Clostridium*	UP000000814
*Clostridium botulinum* ATCC 3502	Causative agent of botulism in humans	UP000001986
*Corynebacterium diphtheriae* NCTC 13129	Causative agent of diphtheria in humans	UP000002198
*Cutibacterium acnes* subsp. acnes str. NBRC 107605	Human skin bacterium	UP000226191
*Deinococcus radiodurans* ATCC 13939	Radiation-resistant bacterium	UP000002524
*Desulfovibrio desulfuricans* IC1	Sulfur reducer found in soil and gut	UP000297065
*Enterococcus faecalis* V583	Opportunistic pathogenic human gut bacterium	UP000001415
*Escherichia coli* str. K-12 substr. MG1655	Model bacterium in research	UP000000625
*Halorhodospira halochloris* DSM 1059	Thermophilic alkaliphilic halophilic phototrophic purple sulfur bacterium	UP000218890
*Helicobacter pylori* G27	Causative agent of gastric ulcers	UP000001735
*Heliobacterium modesticaldum* Ice1	Thermophilic anaerobic clostridium that obtained photosynthetic machinery via horizontal gene transfer	UP000008550
Mitochondrial sequences from *Homo sapiens* hg38	Evolutionarily distant relative of bacteria for comparison	UP000005640
*Klebsiella pneumoniae* subsp. pneumoniae HS11286	Causative agent of human pneumonia and urinary tract infections	UP000007841
*Lactobacillus acidophilus* ATCC 700396	Common gut and yogurt bacterium	UP000006381
*Legionella pneumophila* Philadelphia-1	Causative agent of Legionnaire’s disease in humans	UP000000609
*Methanococcus jannaschii* DSM 2661	Archaeon for comparison	UP000000805
*Mycobacterium tuberculosis* H37Rv	Causative agent of tuberculosis in humans	UP000001584
*Neisseria gonorrhoeae* FA 1090	Causative agent of gonorrhea in humans	UP000000535
*Nitrosomonas europaea* ATCC 19718	Nitrifying soil bacterium	UP000001416
*Nostoc punctiforme* PCC 73102	Nitrogen-fixing multicellular photosynthesizer	UP000001191
*Pseudomonas aeruginosa* PAO1	Common opportunistic pathogen in humans	UP000002438
*Pseudomonas syringae* pv. *tomato* str. DC3000	Model plant pathogen	UP000002515
*Ralstonia solanacearum* RS638	Plant pathogen	UP001164049
*Rhodospirillum rubrum* ATCC 11170	Free-living purple non-sulfur bacterium, nitrogen fixer	UP000001929
*Rickettsia rickettsii* str. Iowa	Causative agent of Rocky Mountain spotted fever in humans	UP000000796
*Salmonella enterica* subsp. enterica serovar Poona	Causative agent of Salmonella food poisoning in humans	UP000298196
*Shigella dysenteriae* serotype one str. Sd197	Causative agent of dysentery in humans	UP000002716
*Staphylococcus aureus* subsp. aureus NCTC 8325	Opportunistic skin pathogen in humans	UP000008816
*Streptococcus pneumoniae* str. NCTC 7466	Causative agent of bacterial pneumonia in humans	UP000001452
*Streptococcus pyogenes* SF370	Causative agent of strep throat in humans	UP000000750
*Streptomyces griseus* subsp. griseus NBRC 13350	Produces streptomycin and other antibiotics	UP000001685
*Synechococcus elongatus* PCC 6301	Model cyanobacterium	UP000001175
*Thermotoga maritima* MSB8	Extremophile with rRNA modification data available	UP000008183
*Thermus thermophilus* HB8	Extremophile with rRNA modification data available	UP000000532
*Treponema pallidum* subsp. pallidum str. Nichols	Causative agent of syphilis in humans	UP000000811
*Vibrio cholerae* serotype O1 (strain ATCC 39315/El Tor Inaba N16961)	Causative agent of cholera in humans	UP000000584
*Vibrio parahaemolyticus* substr. RIMD 2210633	Causative agent of gastroenteritis from undercooked seafood in humans	UP000002493
*Yersinia pestis* CO92	Causative agent of plague in humans	UP000000815

^*a*^ Bacterial species were selected as a broad representation of the domain *Bacteria*, with preference given to species of biological importance (either environmentally, medically, or in scientific research), and those with experimentally validated modification data available. One archaeon and human mitochondria were included for comparison.

Overall, conservation of rRNA modifications and the associated enzymes across bacteria is highly variable between rRNA positions (Fig. 3 and 4). Some modifications are classified as universal due to their presence in nearly all characterized strains. One such example is m^6,6^A1518/1519 in the 16S rRNA. RsmA, the enzyme responsible for this modification, is found across members of the bacterial phyla *Pseudomonadota, Thermodesulfobacteriota, Campylobacterota, Spirochaetota, Chlamydiota, Bacillota, Cyanobacteriota, Actinomycetota, Methanobacteriota, Aquificota, Thermotagota, Deinococcota*, the archaeon *Methanococcus jannaschii*, and human mitochondria, and the modification has been confirmed in many of these taxa. Other highly conserved 16S rRNA modification enzymes are those catalyzing the 16S rRNA modifications m^4^Cm1402 (RsmH/RsmI) and m^7^G527 (RsmG). Both m^6,6^A1518/1519 and m^4^Cm1402 are located in the decoding center of the ribosome, highlighting the importance of these modifications for proper ribosome functioning.

In the 23S rRNA, the most highly conserved modification is m^2^A2503 (catalyzed by RlmN), which is found in *Pseudomonadota, Thermodesulfobacteriota, Campylobacterota, Spirochaetota, Bacillota, Cyanobacteriota, Actinomycetota, Methanobacteriota, Aquificota, Thermotagota,* and *Deinococcota*. Other broadly retained modifications include Um2552 (catalyzed by RlmE), m^3^Ψ1915 (catalyzed by RluD and RlmH), and Ψ1911/1917 (catalyzed by RluD). Um2552 is present within the PTC, a strongly conserved region that is integral to protein production. Positions 1911, 1915, and 1917 of the 23S rRNA are within the subunit bridge, meaning that the modifications in these positions are key to correct ribosome assembly. Thus, the most conserved modifications in the 23S rRNA are associated with basal ribosome function, explaining their broad retention across the bacterial tree of life.

**Fig 5:**
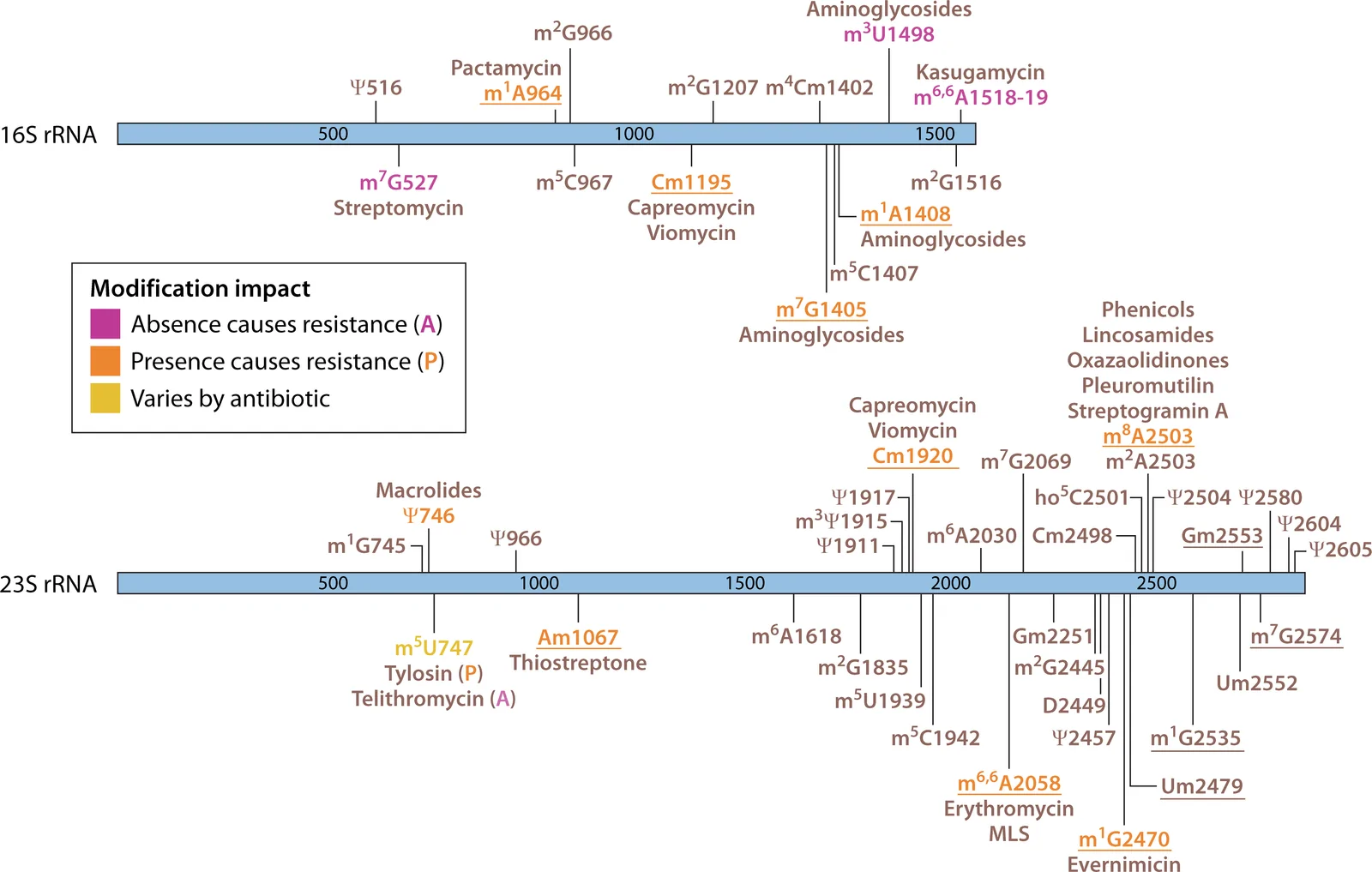
Linear overview of rRNA modifications. For modifications associated with antibiotic resistance, the relevant antibiotic is shown next to the modification. Underlined modifications are not found in *E. coli* MG1655. For m^5^U747, tylosin resistance is caused by modification presence (P), whereas telithromycin resistance is caused by modification absence (A).

In contrast to such universally conserved modifications, others associated with stress-specific responses are taxonomically restricted. For example, m²G1516 in the 16S rRNA, which is associated with temperature adaptations, has been reported only in the phyla *Pseudomonadota* and *Bacillota*. Similarly, only members of the phylum *Actinomycetota* have been determined to contain Cm1409 in the 16S rRNA, a modification that has roles in antibiotic tolerance and virulence (Fig. 5). These evolutionarily unique modifications represent specific environmental adaptations that presumably developed relatively recently in comparison to the universally conserved modifications required for ribosome assembly and protein synthesis. Indeed, the gene enabling formation of m^7^G1405 in the 16S rRNA (*armA*) is commonly found on highly transmissible plasmids associated with antibiotic resistance (262–264), implicating horizontal gene transfer in bacterial acquisition of some rRNA modifications.

Unique rRNA modifications are particularly abundant among members of the genus *Streptomyces*. This is because *Streptomyces* species are prolific producers of antibiotic compounds, many of which inhibit bacterial growth through interference with ribosome function. Production of these compounds entails exposure to them, necessitating self-protective mechanisms. Thus, various *Streptomyces* species have evolved the ability to modify their own rRNA to inhibit interactions with the antibiotics they produce. Examples include the 16S rRNA modification m^1^A964 and the 23S rRNA modifications Am1067 and Um2479 (Fig. 5). Such modifications are often identified through analysis of antibiotic-resistant environmental isolates, a strategy that could be used to discover many more unique rRNA modifications in the future.

### rRNA modifications as stress-responsive regulators

Taken together, studies of many rRNA modifications (e.g., m⁵C, m^4^Cm, and m⁶^,^⁶A) show that these modifications are not simply static structural features; many are located in the decoding center or the PTC (Fig. 2), where they influence critical processes such as tRNA binding, codon recognition, or peptide bond formation. Importantly, several modifications exhibit stress-responsive changes, with quantifiable increases or decreases under stress conditions such as high temperature, ROS treatment, nutrient limitation, or antibiotic exposure. These findings suggest that bacteria can produce heterogeneous ribosome populations with varied modification states rather than a single uniform pool (28, 31). The available data thus support the idea that bacteria undergo ribosomal remodeling during stress. The mechanisms controlling this remodeling and their effects on proteins remain important open questions.

## tRNA MODIFICATIONS

Since the discovery of Ψ in yeast RNA by Davis and Allen in 1957 (265), more than 148 nucleotide modifications have been reported in the tRNA. The available literature regarding tRNA modifications has increased exponentially since 2000, with more than 80% of all articles on this topic having been published in the past 25 years. This explosion in knowledge of known tRNA modifications and their roles is largely due to key methodological advances, such as sequencing-based modification detection methods.

**Fig 6:**
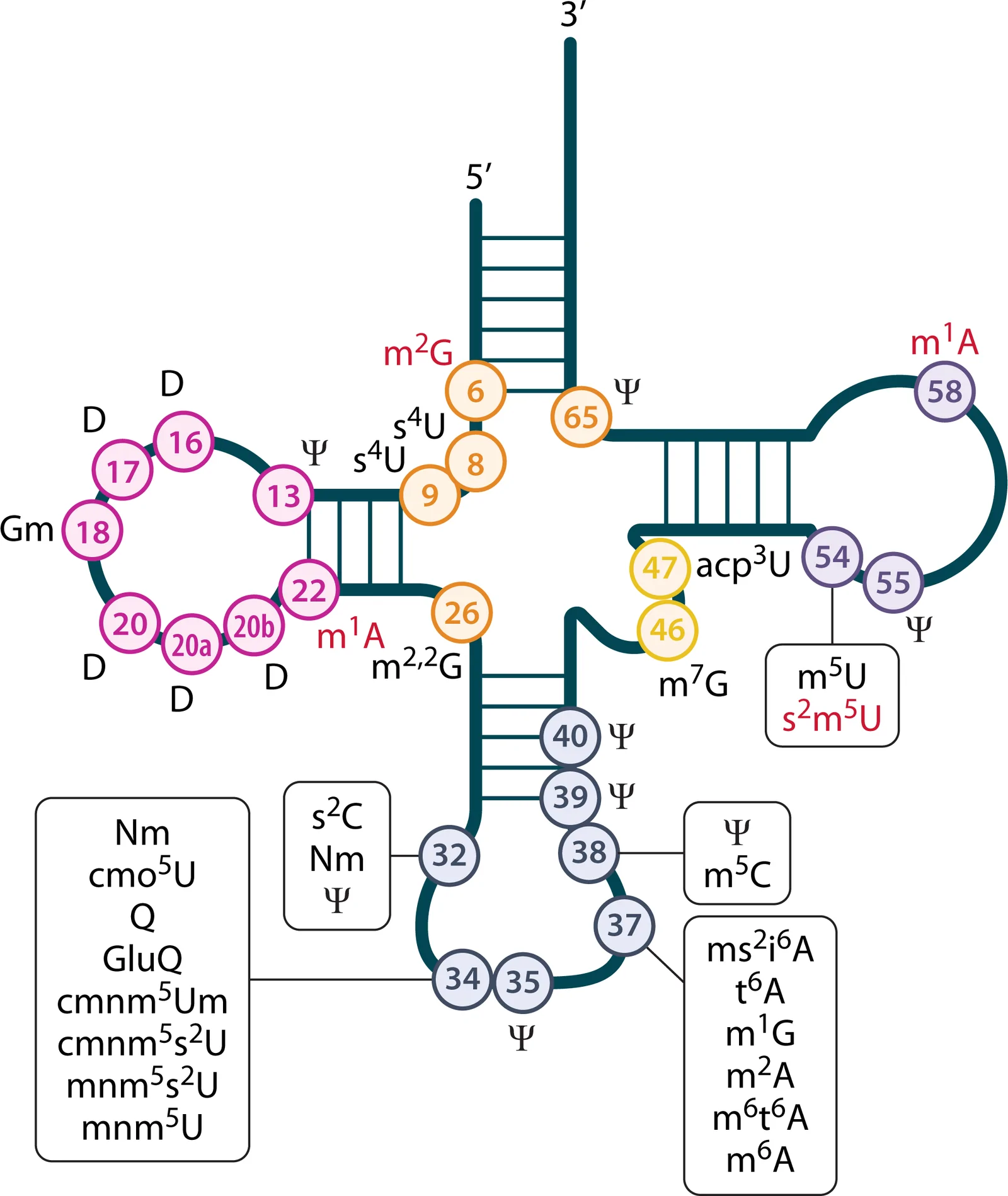
Model tRNA with clover structure. Modifications named in black are found in *E. coli*; those in red are found only in other species. Position color corresponds to structural tRNA feature: D-loop (pink), anticodon loop (blue), variable loop (yellow), T-loop (purple), or other region (orange).

Each tRNA molecule contains an average of ~10 modifications. These are present throughout the RNA, from relative position 6 (near the beginning of the D-arm) to position 65 (at the end of the T-arm) (Fig. 6). tRNA modifications cover a vast range of chemical reactions, including isomerization, reduction, thiolation, hydroxylation, acylation, cyclization, deamination, and alkylation. This chemical diversity allows for adjustments to the degree of structural rigidity or flexibility of tRNA and for the modulation of interactions with the ribosome and mRNA.

tRNA modifications in the D- and T-loops are typically linked to folding and structural integrity, whereas those in the anticodon loop are generally related to translation fidelity (e.g., preventing frameshift events or allowing degenerate decoding). However, these location-based functions are not as strict as initially thought; recent studies have demonstrated that some structural modifications also impact translational events and broader biological responses (43). Furthermore, these molecular mechanisms have recently been related to essential regulatory pathways, stress adaptations, and virulence, including antibiotic susceptibility. Research on modification dynamics during stress adaptation processes has uncovered a previously hidden bacterial regulatory layer. The following sections focus on individual modifications throughout each region of the tRNA, with discussion of specific mechanisms and related phenotypes. Position numbers are consistent with standard tRNA numbering (266).

### D-loop

#### Pseudouridine (Ψ) at position 13

Position 13 is located at the end of a stem loop before the D-loop, where it makes structural contact with position 22. Due to its location and the specific conformation favored when Ψ is present, this modification is suggested to have a role in the structural stabilization of tRNA^Glu(UUC)^ (267, 268). Pseudouridylation at this site in tRNA^Glu(UUC)^ is catalyzed by TruD (43, 269). *V. cholerae* Δ*truD* mutants were recently shown to have decreased motility compared to the wild-type strain (136), suggesting that the modification is important for correct translation of motility-related genes. Furthermore, *truD* deletion increases stop codon readthrough during tobramycin exposure (43). This indicates that Ψ is necessary for maintaining the correct structure of some tRNAs; structural changes due to the presence of mutations can impact decoding processes, inducing translation errors and phenotypic changes such as decreased motility.

#### Dihydrouridine (D) at positions 16, 17, and 20

**Fig 7:**
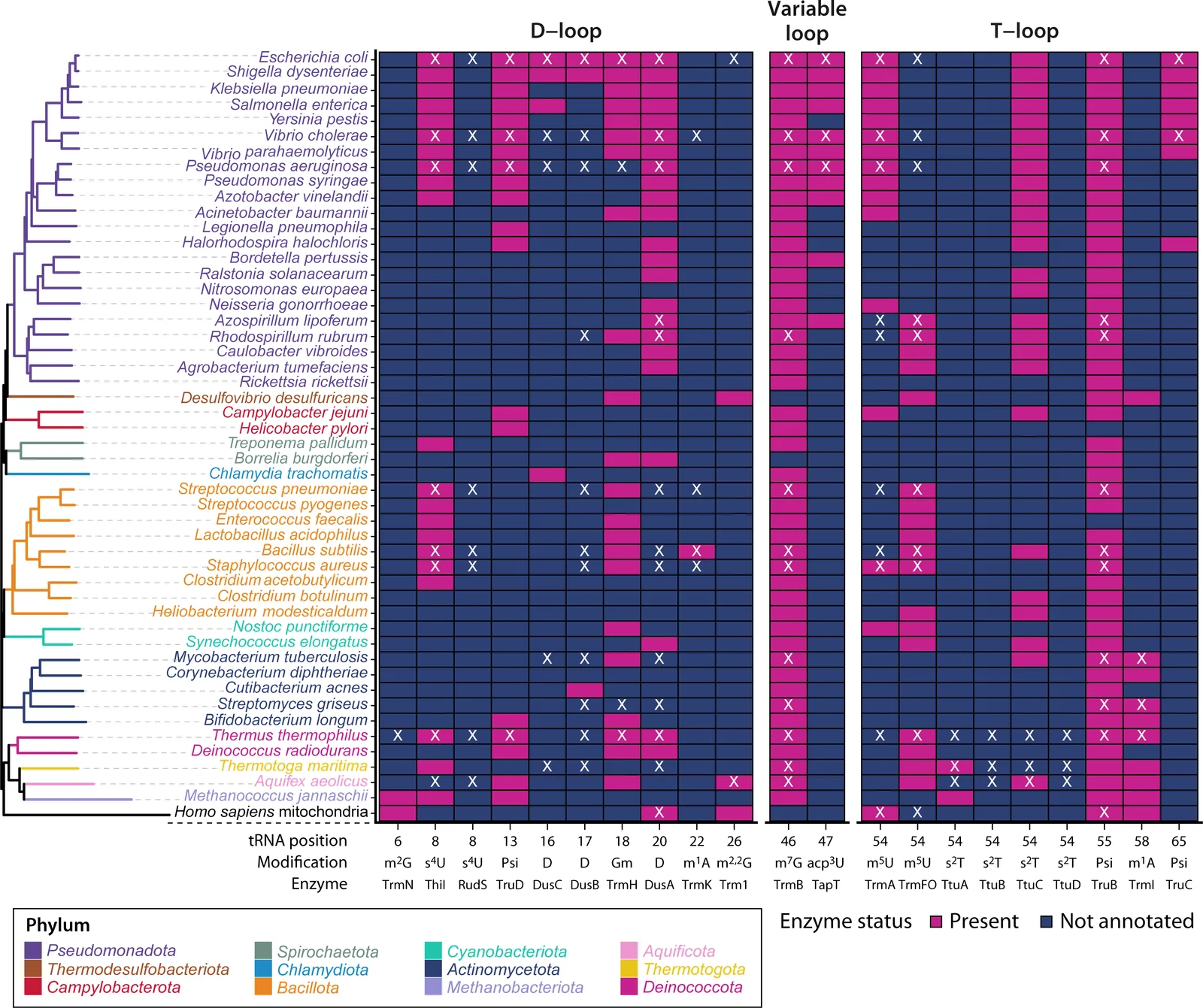
Conservation of modifications and modification enzymes in the bacterial tRNA structural loops. Enzymes catalyzing each modification type are classified as present or not annotated. X indicates that the presence of a modification at this site has been confirmed.

D is a highly conserved tRNA modification found in all domains of life (Fig. 7). In bacterial tRNAs, D is located in the D-loop at positions 16, 17, and 20/20a/20b (270). Formation of D is catalyzed by enzymes of the Dus family, which use nicotinamide adenine dinucleotide phosphate (NADPH + H^+^) and flavin mononucleotide (FMN) as cofactors for U-to-D conversion (271). D formation in the tRNA requires a pre-modified substrate; the responsible enzymes bind more efficiently to molecules that already have a D-loop/T-loop elbow (272). In gram-negative bacteria, Dus enzymes are substrate-specific (e.g., DusA is specific for position D20/20a, DusB for position 17, and DusC for position 16) (269). However, in gram-positive bacteria, Dus enzymes can generally modify multiple positions. For example, DusB modifies positions U17, U20, and U20a in *S. aureus* (273) and *S. pneumoniae* (274)*,* and DusB1 and DusB2 exhibit functional redundancy for multiple positions in *B. subtilis* (271).

In the context of the tRNA, the presence of D modifications increases backbone flexibility (275). Several groups have therefore proposed that D plays an important role in tRNA structural stability (276). This is supported by observations of low D abundance in the tRNAs of thermophiles (276), high D abundance in psychrophiles (277), and DusB downregulation in *Clostridium botulinum* under heat stress (278). Functionally, D-modifying enzymes have been linked with translational fidelity through at least three mechanisms: prevention of ribosomal −1 frameshifting; improvement of Gly, Pro, and Ala codon decoding efficiency; and deregulation of tRNA-modifying enzymes. Changes in the D-loop (namely A25G substitution in tRNA^Leu(UAA)^) reportedly increase translation speed at the expense of fidelity (43, 279).

Several studies have demonstrated the negative impacts of D loss. For example, Δ*dus* strains of *B. subtilis* show decreased growth rates at low temperatures compared to the wild type (271). However, a lack of DusC promotes *V. cholerae* growth in the presence of aminoglycosides when DusA and DusB remain active, increasing fitness (43). Relatedly, D appears to be involved in bacterial stress responses. Some *E. coli* tRNAs show decreased D levels under heat stress (30), and a lack of DusB increases H_2_O_2_ sensitivity in *V. cholerae* (280).

Outside of its post-transcriptional and -translational functions, a recent study found that DusB plays a role in redox regulation in *V. cholerae*, independent of its tRNA modification activity (280). In that study, DusB variants without D-modifying activity were still required to maintain NADPH homeostasis, allowing cells to grow under oxidative stress while maintaining pentose phosphate pathway activity (280). Thus, D-catalyzing enzymes appear to function as active regulatory nodes in the oxidative stress response. The available data therefore indicate that the D system plays a complex role in stress adaptation, tuning the translation process in response to cellular conditions.

#### 2′-*O*-methylguanosine (Gm) at position 18

Functionally, Gm18 serves to stabilize the tRNA structure and contributes to the formation of the classic tRNA “clover” shape through interactions with Ψ55 (281). It also promotes correct reading of the stop codon UAG (282), thus playing an important role in translational fidelity. Indeed, a lack of Gm18 increases the frameshifting rate by 40% at a specific UAU-UAG codon sequence downstream of the P-site (282). Gm18 also forms part of a tRNA modification circuit between the D and TΨC loops, with the presence of each modification affecting levels of the others. In *T. thermophilus*, Gm levels are unusually high when m^7^G46 is present (283), but Ψ55 is absent (284). This lack of Ψ55, combined with the resulting high Gm18 levels, leads to rigid tRNAs that are unable to function properly at low temperatures (284). Together, these data demonstrate functions of Gm in tRNA structure, translational processes, stress responses, and adaptation.

#### *N*^1^-methyladenosine (m^1^A) at position 22

m^1^A at position 22 interacts with A/G13 (285) and is produced through the activity of TrmK, a SAM-dependent enzyme (285). The functional importance of this modification varies between species. For example, *B. subtilis* Δ*trmK* knockouts have no abnormal phenotype (286), but TrmK is essential in *S. aureus* during infection and in *S. pneumoniae* (287, 288). Notably, there are no known mammalian homologs of TrmK; given its apparent importance in streptococci, it has therefore been proposed as a target for novel antibiotics (288).

### Anticodon loop

#### Position 34 (wobble position)

##### Queuosine (Q)-based modifications at position 34

Q and the derivative modification glutamyl-Q (gluQ) are found at the wobble position of tRNAs decoding Asn, Tyr, His, and Asp, which otherwise contain a G in this position (289–291). Synthesis of Q-based modifications requires multiple enzymatic reactions, which have previously been reviewed in detail by de Crécy-Lagard et al. (292). As a brief summary, GTP serves as the initial substrate in a series of reactions catalyzed by FolE, QueD, QueE, QueC, QueF, Tgt, QueA, QueG, QueH, and GluQ, several of which are dependent on metallic cofactors (292). Similar to other organisms (e.g., humans), some bacterial species (such as members of *Actinomycetes*, *Deinococcus*, *Clostridia*, *Lactobacillales*, and *Chlamydia*) do not endogenously produce Q, but rather rely on salvage mechanisms (293–295). Salvage can occur at any of several steps, with bacteria obtaining queuine (the nucleobase containing Q) or any of the precursor molecules.

Functionally, there is a significant difference in ribosomal binding efficiency between Q-modified and unmodified tRNAs. For example, tRNA^Tyr(QUA)^ has twice the binding efficiency of tRNA^Tyr(GUA)^ (296). In *S. enterica* serovar Typhimurium*,* Q deficiency can therefore induce frameshifting due to slow entry of tRNA^Tyr(GUA)^ and tRNA^His(GUG)^ to the A-site during decoding of UAU and CAU, but not during decoding of their C-ending cognate codons (297). In *E. faecalis*, Q levels increase sharply during the late lag phase and then decrease throughout the log and stationary phases. Notably, Q levels change independently of the abundance of the corresponding tRNA isoacceptors, indicating that modification status, rather than simply tRNA quantity, is a primary regulatory driver (298). Due to the requirement of Q34 for proper decoding of NAU codons, Díaz-Rullo & González-Pastor identified genes with an especially high NAU codon frequency in 20 bacterial species, classifying them as “Q-genes” (299). These genes were enriched in functional annotations including fimbrial biogenesis, quorum sensing, biofilm formation, and pathogenesis. These findings highlight the importance of Q-pathway modifications in specific traits.

Q-based modifications have been implicated in many bacterial stress responses. For example, a recent study found that acid stress increased Q abundance among the four Q-containing *E. coli* pre-tRNAs (31). Furthermore, genes previously found to have improved translational efficiency (TE) under acid stress contained a higher frequency of codons requiring Q34 for proper decoding than genes with lower or unchanged TE in response to acid stress. These genes were enriched in functional annotations associated with membrane transport and cell motility (31).

Transcriptome analysis of an *E. coli* Δ*tgt* mutant identified effects on sulfur- and iron-related genes, including genes related to other tRNA modifications, namely *N*^6^-isopentenyladenosine (i^6^A), *N*^6^-threonylcarbamoyladenosine (t^6^A), and 2-lysylcytidine (k^2^C) (300). The mutants were also more sensitive to oxidative stress than the wild type, as the intracellular ROS remained high (300). This involvement of Q in ROS responses is supported by the observation of OxyR induction in a *V. cholerae* Δ*tgt* mutant (301). Separately, *tgt* deletion in *V. cholerae* reportedly confers increased survival after UV exposure (302). Furthermore, in *A. baumannii*, imipenem treatment initiates the oxidative stress response and upregulates QueC at the translational level, suggesting that Q availability is increased to relieve oxidative stress (303). Cellular defense mechanisms are also impacted by Q loss; in *E. coli*, *queF* deletion leads to cellular aggregation and disruption of biofilm formation (299), and *B. subtilis* Δ*queF* mutants exhibit impaired sporulation and biofilm formation, similar to observations in *Shewanella glacialimarina* (304).

Several human pathogens rely on Q for proper decoding of virulence factors, leading to a proposal of the Q pathway as a target for the development of new antibiotic molecules (299). With regard to existing antibiotics, *tgt* deletion in *V. cholerae* causes a loss in fitness during growth at sub-minimum inhibitory concentrations of tobramycin, gentamycin, and triclosan (an inhibitor of the fatty acid biosynthetic pathway) (305). Nevertheless, the minimum inhibitory concentrations of these antibiotics remain the same in the wild-type and deletion strains. In *E. coli*, *tgt* mutants have increased susceptibility to streptomycin and gentamycin, pointing toward negative impacts on translation accuracy when the modification is absent (300).

Q modifications can also influence host-pathogen relationships, with bacteria in the role of either host or pathogen. For example, a defensive endonuclease produced by *E. coli* (colicin E5) specifically targets unmodified tRNAs that would typically contain Q modifications, potentially functioning as a defense mechanism against viral tRNAs (306). Enterohemorrhagic *E. coli* (EHEC) uses norepinephrine (NE) to establish sites of colonization and produce virulence factors; bacteria exposed to NE show a decrease in Q levels, an effect that is not explained by changes in tRNA abundance, but rather by the decrease in Tgt. The observed changes in modification levels appear to correlate with changes in protein translation during NE exposure (32, 33). In *Shigella flexneri*, *tgt* deletion decreases production of the virulence-related transcriptional factor VirF (307). In *Listeria monocytogenes*, the virulence factors internalin and listeriolysin O are encoded by Q-genes, as is the gene encoding the *H. pylori* toxin VacA (299).

In bacteria such as *A. baumannii* and *E. coli*, Q modifications appear to be involved in some aspects of metal metabolism (300). For example, bacterial infection of vertebrates triggers host production of the Zn^2+^ chelator calprotectin, which stymies Q production; to counteract this, *A. baumannii* upregulates a “low-zinc” QueD paralog that slows metal release and enables the use of alternate divalent cations as cofactors (308). In contrast, the presence of Q modifications is deleterious during exposure to high (millimolar concentrations) of Co^2+^ or Ni^2+^. Nickel exposure triggers upregulation of *rpoS* and oxidative-stress genes but repression of *tgt* and *queF*; relatedly, Δ*tgt* mutants show improved survival under high Ni^2+^ conditions (300).

Q is also an important regulator of cold-active growth in the marine bacterium *S. glacialimarina* (309). Under cold stress, Q levels are modulated in an isoacceptor-specific manner. Specifically, Q levels at 0°C are significantly increased in tRNA^Asp^ but decreased in tRNA^His^ and tRNA^Tyr^. Because Q functions as a protein quality control factor via regulation of translation speed and decoding fidelity, it maintains protein homeostasis at low temperatures. Consistent with this physiological role of Q, Δ*tgt* mutants exhibit a pronounced cold-sensitive phenotype, with severe growth delays under cold conditions (309). Q deficiency also impairs the function of tRNA^His^, resulting in ribosome stalling at the *hisL* leader sequence and subsequent upregulation of the entire histidine biosynthesis operon. In addition, the loss of Q triggers a phosphate starvation-like response, leading to activation and upregulation of the Pho regulon (309).

##### 5-hydroxyuridine (xo^5^U) family modifications at position 34

The modifications comprising the xo^5^U family are 5-hydroxyuridine (ho^5^U), 5-carboxymethoxyuridine (cmo^5^U), 5-methoxyuridine (mo^5^U), and 5-methoxycarbonylmethoxyuridine (mcmo^5^U). The xo^5^U pathway begins with hydroxylation of U, which is catalyzed by TrhP or TrhO (310). These two enzymes have different mechanisms: TrhP is prephenate-dependent, whereas TrhO is dependent on oxygen. The resulting ho^5^U can be methylated to mo^5^U by TrmR (which uses SAM as a methyl donor) or carboxylated to cmo^5^U by CmoA and CmoB. Specifically, CmoA forms S-carboxymethyl-S-adenosyl-L-homocysteine (SCM-SAH) using SAM and prephenate as substrates, then CmoB produces cmo^5^U from SCM-SAH and ho^5^U. In *P. aeruginosa,* mo^5^U can still be generated from SAH by CmoB in the absence of SCM-SAH (269). cmo^5^U can be further modified to mcmo^5^U by CmoM, again using SAM. Finally, TrmL can methylate the O2′ group of the ribose to form mcmo^5^Um (310).

Most xo^5^U-modified tRNAs are preferentially modified by either ThrP or ThrO. For example, the cmo^5^U34 in tRNA^Leu(UAG)^ is only generated by ThrP. Nevertheless, ThrP and ThrO are functionally redundant for a subset of tRNAs, such as tRNA^Ser(UGG)^, tRNA^Ala(UGC)^, tRNA^Val(UAC)^, and tRNA^Thr(UGU)^. Due to these unique and overlapping functions, both Δ*thrP* and Δ*thrO* deletion mutants have decreased (but not eliminated) xo^5^U content. Similarly, functional redundancy in the hydroxylation pathway means that lower levels of ho^5^U34 can still be detected in knockout strains for enzymes in the prephenate biosynthetic pathway (e.g., Δ*aroC*), despite the prephenate requirement of ThrP (310).

Unlike gram-negative species, gram-positive bacteria contain two ThrP-type enzymes (ThrP1 and ThrP2) to generate ho^5^U. The gram-positive species *B. subtilis* furthermore has an unusual pathway for xo^5^U generation. Instead of catalyzing the formation of cmo^5^U, it generates only mo^5^U from ho^5^U. This process is mediated by TrmR, which uses SAM to methylate ho^5^U (310). Other gram-negative taxa (e.g., *Bartonella* spp.) contain ho^5^U but not cmo^5^U (311).

xo^5^U modifications are found in tRNAs decoding Pro, Ala, Ser, Val, Thr, Leu, and Gly (2), which are responsible for decoding NYN codons. The presence of an xo^5^U modification expands the range of tRNA recognition to three or four synonymous codons with identical sequences in the first two nucleotides (273, 312). Crystal structures have shown that O5 of cmo^5^U34 allows the modified base to pair with a G nucleotide in Watson–Crick base geometry. Although data from a mutant lacking *cmoB* indicate that the partially modified anticodon ho^5^UGA decodes UCG codons better than the fully unmodified anticodon UGA, cmo^5^U34 is the optimal decoding base for G-ending codons (310). Thus, 5-hydroxylation and related modifications are important for expanded decoding of such codons.

Genetic knockout studies in *E. coli* have demonstrated the functional importance of xo^5^U-family modifications. For example, a Δ*cmoM* strain (which lacks xo^5^U derivative modifications beyond cmo^5^U) has no growth defects; a Δ*cmoB* strain (which has ho^5^U but no derivative modifications) has an increased doubling time; and Δ*trhO*/Δ*trhP* strains (which lack all ho^5^U modifications) have the slowest growth rate. In addition, the Δ*trhO*/Δ*trhP* strain shows temperature sensitivity at both 37°C and 42°C, with a stronger effect at the higher temperature. A lack of xo^5^U34 in tRNA^Ala(UGC)^ and tRNA^Ser(UCA)^ causes +1 frameshifting events (310). Interestingly, mcmo^5^U abundance is increased in tRNA^Ala(UGC)^ pre-tRNAs after exposure to acid stress, demonstrating a potential mechanism for improved translation fidelity under acid-stress conditions (31). In *E. faecalis*, ho^5^U and mo^5^U abundance increase during the lag phase and remain elevated throughout growth (298). ho^5^U stabilizes the anticodon loop to enable pairing with U- and C-ending codons, whereas the methylated form (mo^5^U) promotes pairing with G-ending codons (313). These increases are thought to expand the decoding capacity of tRNAs to support translational efficiency during rapid growth (298).

Additional studies outside of *E. coli* have also implicated xo^5^U modifications in bacterial resilience. For example, *Mycobacterium bovis* tRNA^Thr(UGU)^ undergoes wobble-position remodeling during hypoxia-induced non-replicating persistence, changing from mo^5^U34 to cmo^5^U (314). This change is reversed upon re-aeration. Together with remodeling of tRNA expression levels, alterations in the modification status reprogram translation of ACG- and ACC-enriched proteins (314). A lack of xo^5^U34 in *trhPO* deletion mutants has been found to reduce virulence in a *P. aeruginosa* infection model of *Galleria mellonella* larvae. Blockage of xo^5^U generation also produces metabolic imbalances due to accumulation of prephenate and its precursors, which in turn activate the MexCD-OprJ efflux pump system (315). These findings demonstrate the broad importance of xo^5^U family modifications in normal and stress-responsive cellular functions.

##### 5-methyluridine (xm^5^U) family modifications at position 34

The xm^5^U biosynthetic pathway produces members of the largest tRNA modification family, which comprises 5-methylaminomethyl (mnm^5^U), 5-carboxymethylaminomethyl-2′-O-methyluridine (cmnm^5^Um), 5-carboxymethylaminomethyl-2-thiouridine (cmnm^5^s^2^U), 5-methylaminomethyl-2-thiouridine (mnm^5^s^2^U), 5-carboxymethylaminomethyluridine (cmnm^5^U), 5-aminomethyl-2-thiouridine (nm^5^s^2^U), 5-methylaminomethyl-2-selenouridine (mnm^5^se^2^U), 5-carboxymethylaminomethyl-2-selenouridine (cmnm^5^se^2^U), and 2-thiouridine (s^2^U). The xm^5^U pathway most likely begins with MnmA (316, 317), a [4Fe-4S] cluster enzyme that catalyzes s^2^U formation at the C2 position using ATP (318). Next, MnmE (or TrmE) and MnmG (or GidA) add the cmnm^5^ moiety to U34 using glycine as a substrate. Substituting ammonium acetate for glycine leads to the formation of nm^5^U instead of cmnm^5^U (319–321), which is suggested to be the preferred route in *Aquifex aeolicus* (322). In *E. coli,* cmnm^5^U can also be converted to nm^5^U via FAD^+^-dependent oxidoreduction catalyzed by the C-terminal domain of MnmC in aerobic conditions (319, 320); under anaerobiosis, this reaction is catalyzed by YhcC, a radical SAM-dependent enzyme that is orthologous to MnmL (323). In both cases, the N-terminal domain of MnmC (also known as MnmC2) methylates nm^5^U to mnm^5^U (319, 320). Gram-positive bacteria do not encode MnmC, relying instead on MnmL (a proposed radical SAM-dependent enzyme) together with the SAM methyltransferase MnmM to generate cmnm^5^s^2^U from mnm^5^U (261, 324). In *A. aeolicus*, the preferentially produced nm^5^U is methylated by MnmC2, a homolog of both MnmM and the N-terminal domain of *E. coli* MnmC (322). cmnm^5^s^2^U can be further modified by TrmL (or the ortholog CspR), which methylates the ribose to form cmnm^5^s^2^Um (325, 326). s^2^U modifications can additionally be selenated to Se^2^U by MnmH, which employs SePO_3_^3+^ as a selenium source (327).

Functionally, xm^5^U-family modifications have several important roles. For example, mnm^5^s^2^U modifications prevent frameshifting events during Lys decoding in *E. coli*. Notably, in comparison to the wild-type frameshift rate, the single mutant Δ*mnmA* (which lacks the s^2^ component) has a comparable alteration in frameshifting as the Δ*mnmE* mutant (lacking mnm^5^) (297). However, a lack of mnm^5^U in tRNA^Lys^ can either reduce or increase −1 frameshifting, depending on the slippery sequence (328, 329). Despite the similar phenotypic effects of s^2^ and mnm^5^ loss, mnm^5^U and its C2-modified derivatives (mnm^5^s^2^U and mnm^5^Se^2^U) have been found to differ substantially in thermodynamic stability. This has important implications for translation; the mnm^5^ moiety serves to destabilize codon-anticodon interactions, reducing discrimination between A and G in the third position of NNR codons. The additional presence of s^2^ or Se^2^ in the wobble position of the anticodon fine-tunes the stability of the interaction, with s^2^ and Se^2^ promoting stable interactions with A and G, respectively (312, 330).

Highlighting the importance of xm^5^U modifications in basal cellular functioning, *mnmA* is known to be an essential gene in *C. acetobutylicum* (331), *B. subtilis* (270)*,* and *Streptococcus* species (274). Furthermore, *mnmA* is conserved even in reduced-genome organisms (332). Although *mnmA* is not essential in *E. coli*, *mnmA* deletion mutants have growth defects in rich media under standard culture conditions (318).

In addition to maintaining translation fidelity under normal growth conditions, xm^5^U-family modifications also mediate bacterial stress responses. For example, xm^5^U is implicated in the oxidative and acid stress responses (318). In *B. subtilis*, Δ*mnmEG* mutants show impaired sporulation processes*,* limiting their survival across a range of stress conditions (333). A recent study reported increases in xm^5^U modifications in pre-tRNAs during acid stress treatment (31). Deletion of either *mnmA* or *mnmE* impairs motility and the ability of *E. coli* to withstand acid stress (31, 334).

The acid-sensitive phenotype of xm^5^U-deficient mutants has additionally been reported in species beyond *E. coli*. For instance, Δ*mnmG*, Δ*mnmE*, and Δ*mnmG* Δ*mnmE* mutants of *Streptococcus mutans* are unable to adapt to acid stress and show decreased tolerance to heat, osmotic stress, and bacitracin exposure (335). In *Cronobacter sakazakii*, Δ*mnmG* mutants display acid sensitivity. Interestingly, a similar phenotype is observed in mutants lacking *iscS* or *tusB*, which are associated with sulfur transport to MnmA (336).

xm^5^U modifications also play a role in pathogenicity. For example, in a murine model of *Streptococcus pyogenes* infection, the absence of mnm^5^U derivatives due to *mnmEG* deletion reduces the expression of virulence factors (337). *Salmonella* mutants for *mnmAEG* have decreased fitness in a mouse infection model (338), and *Shigella* requires MnmE for virulence (339). A Δ*mnmG* deletion mutant of *S. enterica* serovar Typhimurium shows filamentous morphology *in vitro,* decreased virulence in a mouse model, and reduced motility (340). However, in *V. cholerae*, MnmG loss improves fitness during exposure to subinhibitory concentrations of aminoglycoside (302). Furthermore, *mnmG* deletion strains have increased resistance to ciprofloxacin and tobramycin. In *P. aeruginosa*, cmnm^5^U impacts virulence regulatory factors, in addition to ROS responses and quorum sensing (341–343).

Interestingly, the only xm^5^U-family modification reported in *M. tuberculosis* is the related modification s^2^U. This modification facilitates *M. tuberculosis* infection in macrophages but has no impact on growth *in vitro*. These findings suggest that virulence genes may be translationally impaired without s^2^U in the wobble position (344). Many other *Actinomycetes* genomes are predicted to contain only one xm^5^U biosynthesis gene, *mnmA*. Despite the near-absence of xm^5^U in *M. tuberculosis* and the predicted lack of relevant enzymes among other *Actinomycetes* species, xm^5^U derivatives have been detected via LC-MS/MS in some members of this class (e.g., *Streptomyces albidoflavus*) (345), suggesting the presence of as-yet undiscovered enzymes in the xm^5^U biosynthetic pathway. Given the available data, modifications in the xm^5^U family can be broadly classified as key components of stress responses and pathogenicity in bacteria.

##### 2-lysidine (k^2^C) and 2-aminovaleramididine (ava^2^C) at position 34

k^2^C is found at position 34 in tRNAs decoding Ile. The enzyme responsible for k^2^C formation is TilS, which binds to the anticodon loop (346). This enzyme uses ATP and L-lysine as substrates (346). Several positive determinants (defined as required structural features for enzyme recognition), particularly in the acceptor stem-loop, aid the enzyme in distinguishing its target tRNA as tRNA^Ile^ (346). One such determinant is *N*^6^-threonylcarbamoyladenosine (t^6^A)37, which can increase TilS activity up to 13-fold (346, 347).

In many species, k^2^C is responsible for correct aminoacylation by IleRS and thus for decoding of the AUA codon as Ile; the absence of k^2^C allows MetRS to instead charge tRNA^Ile(CAU)^ as Met (346, 348, 349). TilS is therefore essential in these species, which include several common model bacteria such as *E. coli*, *V. cholerae*, and *B. subtilis*. However, the gene encoding TilS is lost in *Mycoplasma mobile*, a loss that is compensated by the adaptation of the CAU anticodon to UAU. This change enables AUA decoding without the need for anticodon modification (350). A similar corrective mechanism has been described in *B. subtilis*, in which a thermosensitive *tilS* mutant additionally shows mutation of the tRNA^Ile(GAU)^ anticodon sequence to tRNA^Ile(UAU)^, allowing correct AUA decoding as Ile (351).

The k^2^C derivative modification ava^2^C has also been detected at position 34 of tRNA^Ile^ in *V. cholerae*, *Vibrio parahaemolyticus*, *Aeromonas hydrophila*, *Shewanella oneidensis*, and *Pseudomonas putida* (352). *In vitro*, TilS can generate ava^2^C from 5-aminovaleramide in the presence of high substrate levels. However, formation of ava^2^C *in vivo* is dependent on TilS only for the initial catalysis of k^2^C from C34, and the enzymatic pathway responsible for subsequent conversion of k^2^C to ava^2^C was not characterized (352). The function of this modification is similar to that of k^2^C: the presence of ava^2^C, but not unmodified C, at position 34 of tRNA^Ile^ enables both charging by IleRS and interaction with the AUA codon (352).

##### *N*^4^-acetylcytidine (ac^4^C) at position 34

ac^4^C34 is present in tRNAs decoding Met during elongation (tRNA^eMet^). The modification is proposed to clash with the six-amino group of adenosine; this inhibition of ac^4^CAU–AUA interactions prevents incorrect decoding of AUA codons as Met (353) but enables interaction of ac^4^C34 with the G base in AUG codons (354). In *E. coli*, the corresponding modification enzyme TmcA uses acetyl-CoA and ATP as substrates, whereas the equivalent *B. subtilis* enzyme TmcAL uses acetate and ATP. The target tRNA of TmcAL (tRNA^eMet^) shares a canonical anticodon sequence (CAU) with two other tRNAs, tRNA^Ile2^ and initiator tRNA^Met^ (tRNA^iMet^). TmcAL discriminates between its target and the other two tRNA species based on the identities of the nucleotides at positions 32 and 38 of the anticodon loop (353).

Strains lacking *tmcAL* have impaired growth at low temperatures (< 37°C), possibly due to protein mistranslation. Unmodified tRNA^eMet^ can be charged by IleRS, leading to incorrect translation of Met codons as Ile. Indeed, there are close relationships between k^2^C34 and ac^4^C34 modifications due to their parallel roles in proper decoding of Ile and Met codons, respectively; Δ*tmcAL* strains (which lack ac^4^C) correctly translate Ile but not Met, whereas Δ*tilS* strains (lacking k^2^C) correctly translate Met but not Ile. Cells lacking both *tilS* and *tmcAL* have a stronger growth defect than *tilS* knockouts alone and experience an Ile-to-Met mistranslation rate of up to 17.5% (353).

##### Inosine (I) at position 34

The I located in the wobble position of tRNA^Arg(ICG)^ can pair with C, U, or A (312). This modification is generated by TadA, which catalyzes adenine deamination with a Zn^2+^ cofactor (355). The enzyme is essential in some species, including *E. coli*, *Caulobacter crescentus*, and *V. cholerae*. TadA typically modifies tRNA^Arg(ACG)^, but in *S. pyogenes,* it is also responsible for the modification of tRNA^Leu(AAG)^ (356). In *V. cholerae*, I levels are increased during exposure to sub-lethal concentrations of tobramycin (43), a finding that hints at a potential role of this modification in stress adaptation or translational regulation during antibiotic exposure.

##### 2′-*O*-methylnucleosides (Nm) at position 34

Nm modifications, namely Cm, Um, and Gm, are relatively common at position 34. These modifications are catalyzed by TrmL or the orthologous enzyme CspR (270, 326). TrmL uses SAM as the methyl donor and requires the presence of a pyrimidine in the wobble position and an *N*^6^-isopentenyladenosine (i^6^A) at position 37 (as discussed below) to catalyze the modification (325). Similarly, CspR requires the presence of previous tRNA modifications, particularly i^6^A37, to add Nm modifications to the anticodon. In contrast to TrmL, CspR can also modify G34 (326).

The ribose methylation that forms an Nm modification may improve anticodon loop stability and base-pairing properties by increasing tRNA rigidity. The properties of position 34 are very important for balancing anticodon-codon GC content and can be altered via Nm addition (20, 357). Phenotypically, *trmL* deletion impacts RpoS production levels. However, mutating all UUX Leu codons to CUX (which removes the need for the modified anticodon) in *rpoS* suppresses the need for TrmL (358).

### Position 37

#### *N*^6^-isopentenyladenosine (i^6^A) at position 37

The i^6^A family of modifications at position 37 comprises i^6^A, *N*^6^-(cis-hydroxyisopentenyl) adenosine (io^6^A), 2-methylthio-*N*^6^-isopentenyladenosine (ms^2^i^6^A), and 2-methylthio-*N*^6^-(cis-hydroxyisopentenyl) adenosine (ms^2^io^6^A). Modifications of this family are present in tRNAs decoding Ser, Trp, Tyr, Phe, Cys, Gly, Ile, Leu, and also selenocysteine (Sec) (2). The i^6^A pathway begins with i^6^A37 formation by MiaA, which uses dimethylallyl diphosphate (DMAPP) as a substrate and requires an Mg^2+^ cofactor (359). Formation of ms^2^A (to synthesize, e.g., ms^2^i^6^A) is catalyzed by MiaB, which contains a [4Fe-4S] cluster, uses SAM as a cofactor (360), and requires the A31:Ψ39 pair as a positive determinant (273, 361). Finally, the non-heme diiron monooxygenase MiaE (362) hydroxylates the isopentenyl group to io^6^A, either alone or with 2-methylthiolation (the latter of which is required to form ms^2^io^6^A). Although modifications requiring MiaE have been identified in *Streptomyces* species, no homologous hydroxylation enzyme has yet been described in this genus (345).

i^6^A and its derivatives are known to have critical functions in translation via both direct and indirect means. Directly, i^6^A is required at position 37 by nearly all tRNAs corresponding to UNN codons, stabilizing codon-anticodon interactions and improving translational fidelity (361). Indirectly, i^6^A37 is required by TrmL (and the *B. subtilis* ortholog CspR) as a positive determinant for Nm modifications at position 34 (325). Specifically, the CspR residue R50 interacts with i^6^A37, enclosing the isopentenyl moiety in a hydrophobic pocket and stabilizing the enzyme-tRNA interaction (326). A lack of i^6^A37 will therefore impact other tRNA modifications in the wobble position, reinforcing translation disruption. In *S. flexneri* and *B. subtilis*, ms^2^i^6^A is associated with the translation of the virulence-related transcription factor VirF (307) and proteins related to sporulation processes, respectively (361). ms^2^i^6^A37 hypomodification in *P. aeruginosa* can lead to translational deficiencies under iron deprivation conditions for either consecutive UNN codons or a UNN codon near a rare codon, likely due to ribosome stalling or mRNA/peptidyl-tRNA dropoff (363, 364).

In general, it is difficult to attribute phenotypic effects to a single i^6^A-family modification due to their interrelated nature. Nevertheless, genetic studies using *miaA, miaB*, and/or *miaE* knockout or overexpression mutants have yielded a great deal of information about the i^6^A family of modifications. For example, MiaB overexpression in a Δ*miaA* background still leads to ms^2^A production, suggesting that the i^6^A and ms^2^ modifications may occur independently (345). More functionally, MiaA is known to be required for Hfq and RpoS synthesis (358, 365), demonstrating the importance of i^6^A modifications in global regulatory pathways. MiaA and MiaB deletion strains of *S. enterica* serovar Typhimurium exhibit increased frameshifting rates (between 6-fold and 9-fold) during tRNA^Tyr^ decoding (297). *miaE* deletion mutants also experience an increase in frameshifting, although it is more modest at +20% (297).

i^6^A-family modifications also have key roles in pathogenicity- and stress-related activity in some species. MiaA is required to maintain the virulence of extraintestinal pathogenic *E. coli;* changes in MiaA levels cause −1 and +1 frameshift events, impacting the proteome (365). *E. coli* Δ*miaA* mutants grow poorly in rich media and have diminished oxidative and osmotic stress resistance compared to the wild type (365). In *Agrobacterium fabrum*, Δ*miaA* strains have decreased *vir* expression, mitigating virulence in red potato plants (366). In *Streptomyces albus*, *miaA* deletion alters both morphological status and antimicrobial metabolism (367); specifically, the production of specialized metabolites with antibiotic activity against *Bacillus cereus* is decreased, whereas antifungal activity against *Debaryomyces hansenni* is increased (367).

The presence of an Fe-S cluster in MiaB renders the enzyme (and thus the abundance of thiolated i^6^A modifications) sensitive to the cellular redox status (269). For example, decreased MiaB activity under oxidative stress conditions leads to tRNA^Ser(UGA)^ hypomodification in *E. coli*; hypomodified tRNA^Ser(UGA)^ is less efficient in translating the upstream open reading frame (Upstream of fur [Uof]), which is a prerequisite for *E. coli* expression of Fur (364), indicating that regulation of the translation speed by i^6^A may impact Fur expression (136). The resulting Fur downregulation is an important component of the oxidative stress response due to its minimization of free-iron-related ROS damage. In a similar fashion, Δ*miaA* mutants in *V. cholerae* are characterized by the derepression of Fur-dependent genes (136).

#### *N*^6^-threonylcarbamoyladenosine (t^6^A) at position 37

The t^6^A family comprises the base modification t^6^A, cyclic t^6^A (ct^6^A), and several hypermodifications, namely 6-methyl-*N*^6^-threonylcarbamoyladenosine (m^6^t^6^A), 2-methylthio-N6-threonylcarbamoyladenosine (ms^2^t^6^A), N6-hydroxynorvalylcarbamoyladenosine (hn^6^A), and 2-methylthio-hn^6^A (ms^2^hn^6^A). These modifications are present in the ANN-codon-decoding tRNAs: Asn, Arg, Ile, Ser, Thr, Lys, and Met (2, 312). Importantly, uncyclized t^6^A is not typically found in biological systems, but rather is an artifact of typical extraction protocols; ct^6^A and ms^2^ct^6^A are only detected when RNA is extracted under acidic conditions but digested at neutral conditions (368).

The four principal enzymes required to form t^6^A are TsaC, TsaB, TsaD, and TsaE (369–371). TsaC forms the threonylcarbamoyl-adenylate intermediate species, which is transferred to *N*^6^ of A37 by the TsaBDE complex (372). t^6^A then cyclizes to ct^6^A through the action of the sulfur acceptor protein TcdA (373) in an ATP-dependent reaction, in conjunction with the sulfur-metabolism-related proteins CsdA and CsdE (374). Notably, some thermophilic organisms contain hn^6^A instead of t^6^A (375) due to differences in the specificity of TsaC and TsaD (376).

In tRNA^Thr(GGU)^, t^6^A can be further modified to m^6^t^6^A by TrmO using SAM as a methyl donor (377). TrmO discriminates its target tRNA using G34 as a positive determinant, together with additional bases in the anticodon stem-loop at positions 31–39 (377). Finally, t^6^A and ct^6^A can be transformed to ms^2^t^6^A and ms^2^ct^6^A, respectively, via catalysis by the FeS-cluster protein MtaB using SAM (361, 368).

t^6^A and its derivatives have several documented and hypothesized structural roles, as recently reviewed by Zhang & Westhof (378). Briefly, these modifications may aid in stabilization of the first codon-anticodon base pair (A1–U36); reinforcement of the tRNA-mRNA-ribosome ternary complex; and pre-organization of the anticodon stem-loop structure through prevention of intra-loop Watson-Crick base pairing between U33 and A37. Using molecular dynamics (379), one group posited that t^6^A interacts with the A in ANN codons, promoting base stacking interactions during codon-anticodon pairing. Furthermore, ms^2^t^6^A is reportedly required for efficient AAA and AAG decoding in *Bacillota* (368). However, *Streptococcus* spp. lack ms^2^t^6^A modifications because they do not carry *mtaB* or *tcdA* genes (274), consistent with the loss of Fe-S-dependent enzymes in this group. In species containing ms^2^t^6^A, this modification may function primarily as a component of a modification circuit with the wobble position (273).

As a result of their structural roles, t^6^A modifications enhance translational fidelity and translocation efficiency, aiding in correct decoding of ANN codons. For example, the presence of m^6^t^6^A in tRNA^Thr(GGU)^ improves ACC codon decoding compared to ct^6^A or t^6^A (377). t^6^A-family modification abundance appears to vary between growth phases in species such as *S. mutans* and *B. subtilis*, with multiple studies reporting relatively low levels during the stationary phase and high levels in the exponential phase (270, 368, 380). Increases in t^6^A abundance thus correspond with periods of high protein production, consistent with the expected roles of these modifications in translation.

In *S. mutans*, Δ*tsaE* and Δ*tsaC* mutants (which lack all t^6^A-family modifications) are unusually sensitive to acid and oxidative stress and have deficiencies in biofilm formation (380–382). Furthermore, t^6^A is a positive determinant for the tRNA-cleaving enzyme PrrC, which is activated in response to T4 phage infection (380). The enzyme recognizes t^6^A to indiscriminately cleave both host tRNA^Lys(UUU)^ as a community-protection abortive infection mechanism.

#### *N*^2^-methyladenosine (m^2^A) at position 37

m^2^A37 is catalyzed by RlmN, which contains an Fe-S cluster and uses SAM as a methyl donor and radical generator (242, 269). Consistent with other modifications at position 37, m^2^A37 stabilizes codon-anticodon interactions, preventing frameshift events. Mutants lacking this modification show decreased decoding efficiency for CGU and CGA (Arg) codons in *V. cholerae* and increased UAG readthrough in *E. coli*. However, the latter phenotype could be due to a lack of the rRNA (rather than tRNA) modifications catalyzed by RlmN (43, 242). Genes enriched in codons corresponding to tRNAs with m^2^A at position 37 are associated with motility, translation, and metabolism (43).

In *E. faecalis*, m^2^A levels decrease from lag to log phase and then increase again as the bacteria enter stationary phase. m^2^A is believed to maintain a relaxed tRNA conformation to facilitate decoding (383). Its decline during the lag phase of *E. faecalis* growth may be caused by exposure to ROS, which can decrease m^2^A levels due to inactivation of the Fe-S cluster required for RlmN activity (298). This is consistent with the findings of Lee et al. (242), who noted that RlmN could be a redox-responsive element in gram-positive bacteria. Relatedly, the proteome of an *E. faecalis rlmN* knockout resembles that of the proteome during ROS exposure (242). *Streptococcus*, *Listeria*, and *Enterococcus* species have lost most enzymes containing Fe-S clusters, including some of those modifying tRNAs (242, 274).

#### *N*^1^-methylguanosine (m^1^G) at position 37

A set of tRNAs containing a G36-G37 motif is methylated at position 37 (forming m^1^G) by TrmD (384), which uses SAM as a methyl donor and Mg^2+^ as a cofactor (385). A lack of m^1^G37 results in several translation errors, such as +1 frameshift events, reduced elongation, and decreased tRNA aminoacylation (384, 386, 387). The former effects are due to the inhibition of CCC and CCU decoding by tRNA^Pro(UGG)^; in the absence of m^1^G, the tRNA undergoes anticodon stem-loop structural remodeling that prevents codon-anticodon interactions. Δ*trmD* mutants thus have only tRNA^Pro(GGG)^ to decode Pro codons. This particularly impacts the translation of cold-shock proteins, which are rich in CC[C/U] codons (388). Another function regulated by m^1^G37 levels is the synthesis of the Mg^2+^ transporter MgtA. As noted above, TrmD uses Mg^2+^ as a cofactor. In response to low Mg^2+^ levels, translation of *mgtL,* which is upstream of *mgtA* and is rich in CC[C/U] codons, is stalled. This stalling leaves a Rho binding site covered, promoting *mgtA* transcription and therefore restoring the Mg^2+^ levels (389).

In general, *trmD* deletion mutants are non-viable (e.g., in *S. pneumoniae, P. aeruginosa, Mycobacterium* spp., and *C. acetobutylicum*) or have poor growth (e.g., *E. coli* and *B. subtilis*) (274, 331, 387). A lack of m^1^G means that the aminoacyltransferase ProRS cannot aminoacylate tRNA^Pro^. Nevertheless, a point mutation in *proS* restores aminoacylation of tRNA-Pro, reversing the abnormal growth phenotype (387, 388). In pathogenic bacteria, such as *E. coli* and *Salmonella,* decreased m^1^G37 levels have been found to impair membrane structure; sensitize bacteria against several antibiotic molecules; and suppress the development of resistance. The mechanisms for these effects may be related to impaired translation of Pro codons, which are particularly rich at the beginning of the coding sequence (CDS) in transcripts encoding outer membrane proteins (390). Notably, the apparent importance of m^1^G37 makes TrmD a promising target for antibiotic development.

#### *N*^6^-methyladenosine (m^6^A) at position 37

The prevalence of m^6^A37 varies greatly by species; for example, in *E. coli, V. cholerae*, and *P. aeruginosa*, it is only present in tRNA^Val^. In contrast, *Mycoplasma capricolum* bears m^6^A in 12 of the 24 tRNAs containing an A at position 37 (332). In most species containing m^6^A37, the modification is written by TrmN (also known as TrmM or TrmN6) (270, 391). This function is performed by the dual-functional enzyme RlmF in *P. aeruginosa*, which lacks a TrmM homolog (269). Both enzymes use SAM as the methyl donor (269, 391), but TrmM also requires a divalent ion as a cofactor (391). Like other modifications at position 37, m^6^A37 serves to stabilize the codon-anticodon interaction and limit frameshift events at AU-rich sequences (357).

### Other anticodon stem modifications

#### Pseudouridine (Ψ) at positions 31/32, 38, 39, and 40

Ψ is found at several positions in the anticodon stem, namely at sites 31/32, 38, 39, and 40. Formation of Ψ at site 32 is catalyzed by RluA in gram-negative bacteria (43, 269), with the non-orthologous enzyme YjbO performing the same function in gram-positive species. This difference in enzymes suggests a divergent substrate recognition mechanism (270, 273). A homolog of the human mitochondrial Ψ synthase Pus9 that is capable of modifying position U32 has also been reported in species including *M. tuberculosis*, but *in vitro* activity remains to be confirmed (344, 392). *V. cholerae* requires an extra step to form Ψ32, with TrcP first editing C32 to U32 prior to the U-to-Ψ modification by RluA (43).

In most species, Ψ formation at positions 38 through 40 is catalyzed by TruA. An exception is *M. capricolum*, in which TruA modifies only U39, leaving U38 and U40 unmodified (332). In species bearing this modification, Ψ39 promotes correct structural formation via alignment with A31. In *B. subtilis* and *S. aureus*, the A31-Ψ39 pair is also a positive determinant for methylthiolation of position 37 to form ms^2^i^6^A37 in anticodons corresponding to UNN codons (273, 361).

Generally, pseudouridylation in the anticodon stem promotes RNA stability via hydrogen bonding, improving interactions between bases in this loop (393). Consistent with 2′-*O*-methylation at position 32, Ψ modifications in the anticodon stem stabilize the anticodon loop energetically and geometrically (20, 357). In gram-negative bacteria, *truA* mutants show increased frameshifting frequency of tRNA^Leu(CUA)^ at the A-site (297, 328).

In addition to the anticodon stem, some species (including *E. coli* but not *B. subtilis*, *S. aureus*, or *V. cholerae* [43, 270, 273]) have Ψ at position 35 of tRNA^Tyr(QΨA)^, which is positioned within the anticodon itself. Pseudouridylation at position 35 is catalyzed by RluF, a dual-specificity enzyme that also modifies rRNAs (259). Strains lacking this enzyme experience disrupted translation of proteins containing multiple consecutive tyrosine codons (259) but lack an abnormal growth phenotype under standard culture conditions (259). The requirement of RluF for efficient translation of tyrosine-rich proteins suggests that it may be important for the synthesis of stress-related proteins. However, direct evidence linking Ψ35 to a specific stress response is limited.

#### 2-thiocytidine (s^2^C) at position 32

s^2^C at position 32 is catalyzed by TtcA, which contains a Cys-X1-X2-Cys motif (394) and a [4Fe-4S] cluster. Like other modifications at position 32, 2-thiolation of C32 improves anticodon-loop stability and base pairing during the decoding process (395). Several studies have found that this modification is not essential and that deletion mutant strains have comparable growth rates to wild-type strains. Nevertheless, a lack of s^2^C32 alters Arg decoding, reducing the A-site selection rate or preventing anticodon pairing in some cases (394).

In *P. aeruginosa*, *ttcA* expression is regulated by the oxidative stress response factor OxyR. Typically, oxidative stress causes derepression of *ttcA* by OxyR, leading to s^2^C formation in the anticodon. This, in turn, promotes KatA/KatG (catalase) translation and a subsequent alleviation of oxidative stress. In Δ*ttcA* knockouts, the absence of s^2^C32 causes translation errors in catalase, reducing the number of available enzymes and thus increasing sensitivity to oxidative stress (396). This reduced capacity for withstanding ROS damage leads to attenuated virulence in *Drosophila*. Furthermore, due to the presence of the Fe-S cluster, TtcA itself may play a role in ROS sensing.

#### 2′-*O*-methylnucleosides (Nm) at position 32

Many bacterial tRNAs undergo methylation at position 32 to form Am, Cm, or Um. Am is only reported in *P. aeruginosa* in tRNA^Pro^ (395). All Nm modifications in this organism are installed by TrmJ using SAM as a methyl donor (395). Nm32 modifications are not essential, and no Cm/Um32 modifications have been detected in *B. subtilis* (270). In some species, Nm functions in translation efficiency and stress response pathways. For example, a lack of TrmJ induces H_2_O_2_ susceptibility in *P. aeruginosa* by reducing catalase translation (395). In *V. cholerae*, Δ*trmJ* knockouts show increased stop-codon readthrough in the presence of tobramycin (43). Nm32 is therefore far more than a simple structural modification, with key roles in bacterial physiology and pathogenesis.

### Variable loop

#### *N*^7^-methylguanosine (m^7^G) at position 46

m^7^G46 is catalyzed by TrmB, which uses SAM as a substrate (283). The specificity of this enzyme relies on several factors: variable loop size, variable loop structure, and specific sites on other structural loops, including the T-loop stem (397). In thermophilic bacteria, TrmB possesses a C-terminal region that contributes to stability and nucleotide selection (283).

This modification is implicated in tRNA structural integrity. Its absence causes a decrease in tRNA melting temperature, particularly under extreme environmental conditions (e.g., high temperature [283]). m^7^G46 creates a positive charge under physiological conditions (398), influencing the tRNA tertiary structure. The positively charged modified base interacts with position G22 and C13 at the tRNA core (354, 398). Although position 46 is within the variable loop, not the anticodon loop, its absence reduces the decoding efficiency of Phe and Asp, which impairs translation of peroxide-detoxifying enzymes (18, 283, 399).

m^7^G belongs to a modification network that also includes Gm18, m^1^G37, and m^1^A58 (283). In modification networks such as these, the presence of each modification enables the formation or leads to increases in the abundance of other modifications in the network. Mutants lacking m^7^G therefore have increased frameshift errors due to hypomodification of other positions (such as m^1^G37) that rely on m^7^G for formation (18, 283, 399, 400).

Levels of m^7^G46 are reported to increase in *A. baumannii* during heat stress, oxidative stress, and host infection, the latter of which triggers a range of stressful host immunity mechanisms such as iron chelation, ROS production, and low pH (401). Deletion mutant phenotypes under stress conditions differ between species: *E. coli* shows no significant growth defects, whereas *T. thermophilus* exhibits severe growth restriction at temperatures above 70°C (283). In *P. aeruginosa*, a lack of m^7^G46 reduces survival during oxidative stress (399); this is also true in *A. baumannii*, which additionally experiences attenuated infection in mice and reduced replication in macrophages (401). m^7^G46 therefore appears to be an integral component of the stress response in some bacterial species.

#### 3-(3-amino-3-carboxypropyl)uridine (acp^3^U) at positions 46 and 47

acp^3^U formation at positions 46/47 is catalyzed by TapT, which uses SAM as the aminocarboxypropyl donor (402, 403). In addition to acp^3^U, the acetylated derivative acacp^3^U is present in *V. cholerae*, where it is catalyzed by AcpA (43). Structurally, acp^3^U46/47 increases the thermostability of the tRNA. Functionally, it participates in tRNA maturation, structural stabilization, and translational accuracy under stress conditions, hinders Watson-Crick pairing, and may contribute to optimal tRNA folding (403). Furthermore, acp^3^U46/47 is reportedly part of a modification network that also includes m^5^U54 and Ψ55 (404); the presence of each modification generally leads to increased levels of the other modifications in the network.

The importance of acp^3^U46/47 varies by species. For example, *V. cholerae* Δ*tapT* mutants exhibit growth defects under standard culture conditions and in response to tobramycin exposure. A molecular mechanism underlying this effect has not been reported, but acp^3^U is known to have a protective role under proteotoxic conditions (43). In *E. coli*, genomic instability was reported for Δ*tapT* strains, leading to variable colony sizes and motility patterns (403). However, no abnormal phenotypes have been reported in *E. coli* Δ*tapT* mutants in response to heat stress, paromomycin treatment, or H_2_O_2_ exposure (402). acp^3^U is therefore not critical for tRNA function in the species studied to date.

#### *N*^5^-methylcytidine (m^5^C) at position 49

Historically, m^5^C was considered absent from the variable loop of common bacterial model organisms such as *E. coli*. However, it was recently reported that the rRNA methyltransferase RsmF catalyzes m^5^C49 in tRNA^Tyr(QUA)^ after exposure of *E. coli* to strong oxidative stress and heat shock (405). This stress-responsive appearance of m^5^C was not found in *V. cholerae* (43).

### T-loop

#### *N*^5^-methyluridine (m^5^U) at position 54

m^5^U54 reportedly contributes to tRNA maturation, influencing levels of fellow tRNA modifications acp^3^U47 and ms^2^i^6^A37 (404, 406). It also regulates protein synthesis by modulating the elongation step between peptidyl transfer and subsequent amino acid-tRNA binding and translocation (406). Formation of this modification is catalyzed by TrmFO in *Bacillota* and members of the *Pseudomonadota* classes *α-* and *δ-proteobacteria*, but by TrmA in *ß*-, *γ*-, and *ε-proteobacteria* (407). Although a lack of TrmA has minimal effects on cellular stability, aminoacylation is negatively impacted (404), and there are modest reductions in translation of specific codons (e.g., CAA [Gln] and UGU [Cys]) (404). The impacts of TrmA loss on Leu translation are codon-dependent: translation is decreased for UUG but increased for UUA, indicating that TrmA modulates translational efficiency in a codon- and tRNA-specific manner (404).

In some species, such as *Azotobacter vinelandii* and *Sinorhizobium meliloti,* m^5^U is reported to be absent during growth with ammonia. Nevertheless, under standard growth conditions for these nitrogen-fixing bacteria, m^5^U is present (408). The mechanism that associates nitrogen metabolism with m^5^U regulation is currently unknown.

#### 5-methyl-2-thiouridine (m^5^s^2^U) at position 54

m^5^s^2^U54 is a derivative modification of m^5^U54, typically found in thermophilic bacteria (354). Several proteins are required for the installation of this modification, which occurs via a complex mechanism as previously reviewed (354). The enzymes involved in m^5^s^2^U54 generation are TtuA, TtuB, TtuC, TtuD, and the cysteine desulfurase IscS. Both thiolation and methylation of position U54 are known to contribute to tRNA thermostability in *T. thermophilus* by increasing the rigidity in the T-loop (409); reduced m^5^s^2^U levels due to a lack of m^1^A58 decrease the tRNA melting temperature by 2°C (410). There is evidence of temperature dependence in the 2-thiolation process; thus, thermophiles may dynamically adjust m^5^s^2^U54 levels in response to environmental temperature conditions (410).

#### Pseudouridine (Ψ) at position 55

Ψ55 formation is catalyzed by TruB, which is essential in some species (e.g., *C. acetobutylicum* [331]). In *E. coli* Δ*truB* mutants, aminoacylation is negatively impacted, and Arg and Pro decoding are disrupted (282, 404). This may explain observed fitness losses compared to the wild type in competition experiments. TruB also acts as a tRNA chaperone, which represents a confounding factor in determining the effects of the modification (411). In addition to the lack of fitness, there is also a loss of virulence in an *S. flexneri* strain with a catalytically inactive variant; Ψ55 promotes the efficient translation of proteins required for virulence gene expression (282).

As mentioned above, Ψ55 is part of a tRNA modification network in *T. thermophilus*. A lack of Ψ55 increases levels of Gm, m^5^s^2^U, and m^1^A. This causes excessive tRNA rigidity under low temperatures, preventing sustained translation and thus inhibiting production of cold-shock proteins (284). Ψ55 levels also reportedly decrease during T4 phage infection of *E. coli* (29). Overall, Ψ55 appears to have roles in both basal and stress-responsive protein production.

#### *N*^1^-methyladenosine (m^1^A) at position 58

In addition to its presence in the D-loop, m^1^A is present at position 58 in some bacteria. Its formation is catalyzed by TrmI, using SAM as a methyl donor (344). In *T. thermophilus*, m^1^A58 abundance is dependent on culture conditions and is reported to induce other T-loop modifications such as m^5^s^2^U54 (283). Deletion of *trmI* induces temperature sensitivity in *T. thermophilus*, although this is not due to biochemical impacts of m^1^A. Instead, the absence of m^1^A58 leads to decreases in m^5^s^2^U54, which in turn lowers the thermostability of the tRNA (410).

### Other modifications

#### 4-thiouridine (s^4^U) at position 8

Formation of s^4^U at position 8 is catalyzed by ThiI, which requires a sulfur source for catalysis (412). There are substantial differences in ThiI structure between species, and the sulfur source protein also varies by lineage. For example, *E. coli* and *Mycoplasma* spp. use IscS as a sulfur source, whereas the gram-positive bacteria *B. subtilis* and *S. aureus* employ NifZ (270, 273, 332).

Phenotypically, s^4^U levels have been found to decrease in *E. coli* and *E. faecalis* along with growth rate or phase (298, 413). In *V. cholerae*, s^4^U8 appears to have an important structural role in combination with m^5^U54, Ψ55, and i^6^A37. In the absence of s^4^U8 due to *thiI* deletion, the other three modifications compensate for the resulting decreased structural stability (414). Furthermore, *V. cholerae* Δ*thiI* Δ*truB* and Δ*thiI* Δ*trmA* double deletion mutants show growth defects at 37°C but not at 25°C; this suggests roles in thermodynamic stability for not only s^4^U, but also m^5^U54 and Ψ55 (414).

s^4^U8 is critical for the intracellular stability of tRNAs; structural changes arising from a lack of s^4^U can lead to rapid tRNA destruction by the degradosome, which comprises enolase, RNA helicase, and polynucleotide phosphorylase in *V. cholerae* (414). In the stationary phase of *V. cholerae* growth, the absence of s^4^U in either charged or uncharged tRNA^Tyr^ induces degradosome activity on these molecules (414). A suppressor mutation (RNase E Q664*) prevents degradosome assembly, thus restoring growth in the absence of s^4^U (414). Relatedly, in a P_ara_-*thil*/Δ*truB* strain (which lacks s^4^U and Ψ55), a U51C mutation in tRNA^Tyr^ restores growth by stabilizing the TΨC loop stem, with the sequence alteration compensating for the missing modifications (414).

RudS of *P. putida* was recently found to have desulfidase activity and to reduce s^4^U levels in the tRNA when heterologously expressed in *E. coli* (34). s^4^U8 is a photosensitive residue that crosslinks with C13 when exposed to near-UV radiation; thus, the presence of an s^4^U8 “eraser” such as RudS protects protein synthesis during near-UV radiation exposure (34). Indeed, treatment with near-UV triggers a reduction in s^4^U levels in *Enterobacter cloacae* tRNA (415). This phenomenon has not been observed in *E. coli*, which lacks RudS. Although RudS expression is beneficial in preventing crosslinking, it may lead to degradosome-mediated regulation of some tRNA levels via the mechanism described above.

#### *N*^2^-methylguanosine (m^2^G) and *N*^2^*N*^2^-dimethylguanosine (m^2,2^G) at positions 6 and 26/27

m^2^G and m^2,2^G are present in some tRNAs, with the location of the modification differing by species. For instance, it is present at position 6 in *T. thermophilus*, but position 26 in *A. aeolicus* (416, 417). m^2^G6 is catalyzed by TrmN in *T. thermophilus* (283, 418). In *A. aeolicus*, m^2^G and m^2,2^G at positions 26 and 27 are catalyzed by Trm1 (419). Information is scarce regarding the effects of these modifications in prokaryotes.

#### Pseudouridine (Ψ) at position 65

In addition to the sites noted above in the D-loop, anticodon loop, and T-loop, some tRNAs also contain Ψ at position 65. The modification at this site is catalyzed by TruC (43). Like other tRNA Ψ residues, Ψ65 increases structural rigidity (19, 268). Although TruC has not proven to be an essential protein, its absence does alter readthrough at UAG and UGA codons in *V. cholerae* (43).

### tRNA modification conservation

**Fig 8:**
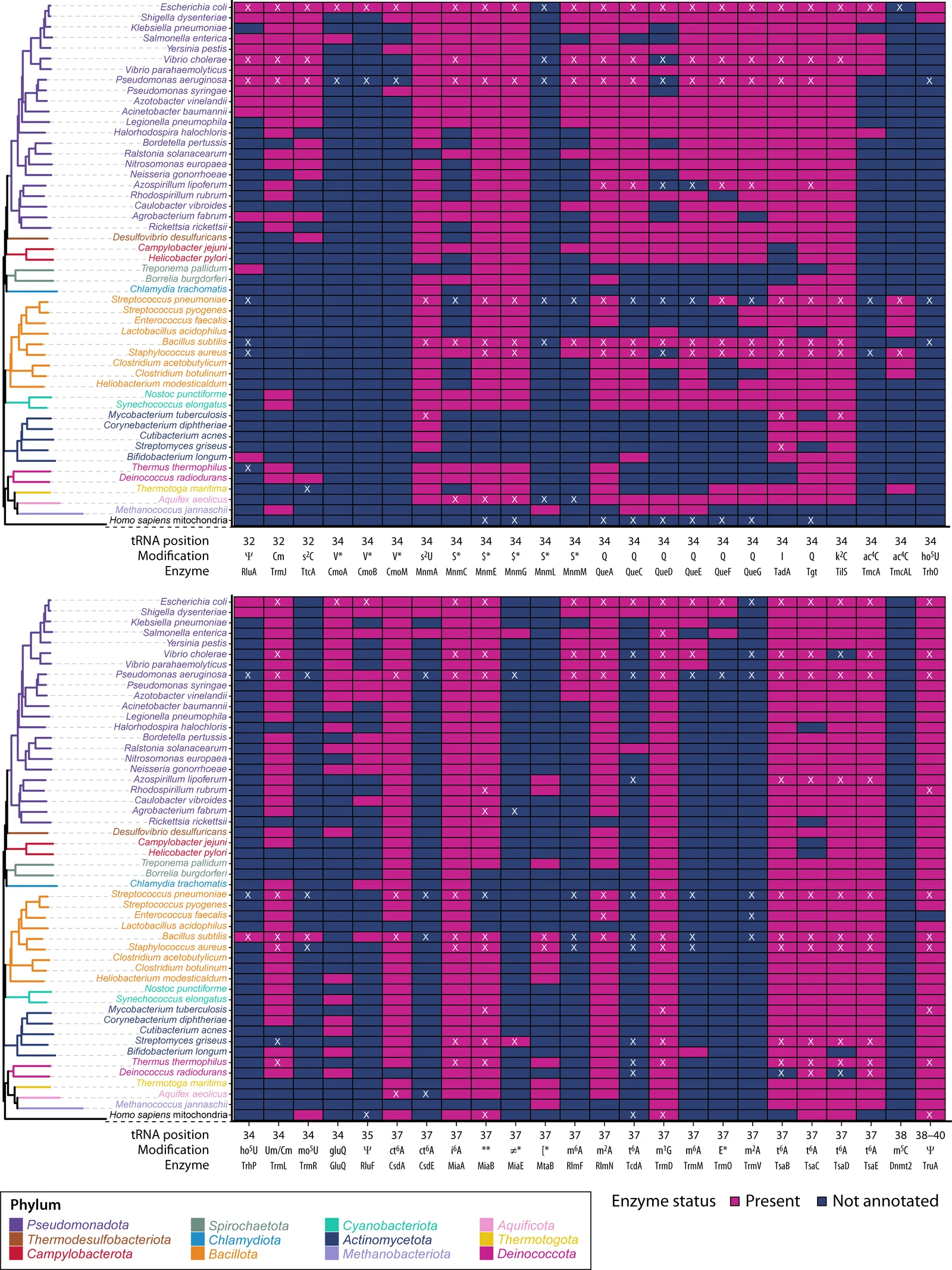
Conservation of modifications and modification enzymes in the bacterial tRNA anticodon loop. Enzymes catalyzing each modification type are classified as present or not annotated. X indicates that the presence of a modification at this site has been confirmed. *Modification name based on those used in the MODOMICS database: V, cmo^5^U; S, mnm^5^s^2^U; $, cmnm^5^U/cmnm^5^s^2^U; *, ms^2^i^6^A; ≠, ms^2^io^6^A; [, ms2t6A; E, m^6^t^6^A.

Due to their critical functions, several tRNA modifications are highly conserved across the domain *Bacteria* (Fig. 7 and 8). Examples include t^6^A37, m^1^G37, Ψ38-40, m^7^G46, and Ψ55. Ψ55 is considered ubiquitous in bacteria, having been identified in members of the phyla *Pseudomonadota*, *Bacillota*, *Actinomycetota*, *Mycoplasmatota*, *Cyanobacteria,* and *Deinococcota*, and even in human mitochondria; it is found in isoacceptors for all 21 amino acids (2). m^1^G37 is found in the same phyla within tRNAs corresponding to Arg, Leu, Pro, Glu, His, Phe, Cys, Gln, Gly, Lys, and Tyr (2). Notably, this modification is conserved across all three domains of life (384), including in reduced-genome organisms (350).

Although several tRNA modifications can be found across the domain *Bacteria*, some species have lost modifications that are otherwise considered universally essential based on their ubiquity. For example, Ψ55 is known to be absent from two *Mycoplasma* species (*M. pneumoniae* and *Mycoplasma genitalium* [332]) and Candidatus *Riesia pediculicola* (350); the thermophiles *D. radiodurans* and *T. thermophilus* have lost the essentiality of t^6^A biosynthesis genes (332, 350). Modification nonessentiality can be enabled by compensatory adaptations. For example, modifications that function as determinants for aminoacyl-tRNA synthetases may be rendered inessential by alterations in these enzymes that allow proper tRNA charging even in the absence of the modification (274). Other compensatory adaptations reflecting evolutionary strategies that support a reduced and specialized set of tRNA modifications (332, 350) may include alternate codon use or substitution of functionally comparable tRNA modifications (350).

Conversely, some taxa have evolved unique tRNA modifications based on the particular needs necessitated by their environment. Taxonomically restricted tRNA modifications are exemplified by thermophiles and psychrophiles; the thermodynamic conditions in which they thrive require biological machineries that can withstand extremely high or low temperatures compared to mesophilic species. In the case of tRNAs, this means maintaining structural stability and decoding properties. For example, m^5^C has been identified in the variable loop of psychrophilic members of the phyla *Pseudomonadota* and *Bacillota* (277), and in members of the thermophilic phylum *Deinococcota* (2, 392). Additionally, m^5^C has also been identified in the anticodon loop (position 38) of tRNA^Glu^ in the thermophile *Geobacter sulfurreducens*. m^5^C has not been consistently identified in the tRNAs of mesophilic species, indicating that this is a unique adaptation to extreme conditions.

### tRNA modifications as stress-responsive regulators

**Fig 9:**
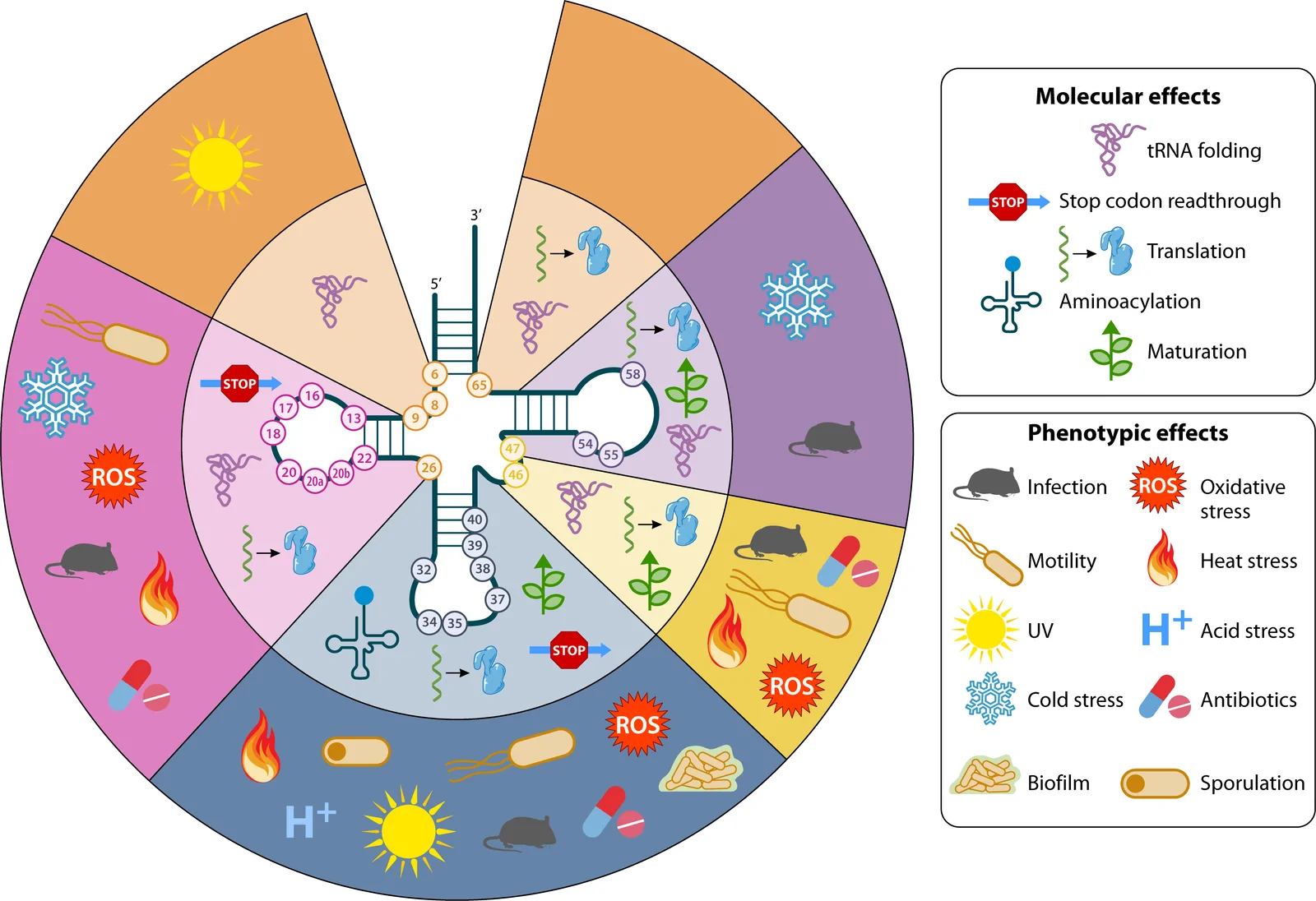
Molecular and phenotypic effects of tRNA modifications. tRNA modifications have broad biochemical impacts, corresponding to molecular processes including aminoacylation and tRNA maturation. These molecular processes in turn have direct and indirect effects on phenotypes such as virulence, motility, and tolerance of various stressors. Modifications in specific regions of the tRNA have varying impacts on molecular processes and phenotypic outcomes, here divided by structural region: D-loop (pink), anticodon loop (blue), variable loop (yellow), T-loop (purple), and other regions (orange). Effects are divided into molecular (pale inner ring) and phenotypic effects (dark outer ring).

tRNAs from across the domain *Bacteria* are highly modified, with modifications functioning in diverse processes related to tRNA structure, codon recognition, and translation fidelity. tRNA modifying enzymes can also act as regulatory nodes and sensors that allow the cell to adapt to environmental changes such as alterations in metal abundance, nutrient availability, or temperature (Fig. 9). Furthermore, they participate in stress responses, for example, by modulating the translation efficiency of stress-response proteins. This can aid cells in surviving under harsh conditions such as oxidative or acid stress. Survival and adaptation to these stresses, in combination with translational regulation of virulence factors, provide bacterial cells with biological strategies to thrive in multiple environments. Prokaryotic tRNA modifications may also serve as targets for novel antibacterial compounds.

## mRNA MODIFICATIONS

In contrast to the rRNA and tRNA, modifications in the mRNA are highly understudied. Information is therefore relatively limited regarding the identities, locations, and functions of mRNA modifications in bacteria. However, recent advances in modification detection methods (e.g., DRS) have enabled an unprecedented look at the bacterial epitranscriptome beyond the tRNA and rRNA. This is a rapidly evolving field, with current knowledge of bacterial mRNA modifications summarized here.

### *N*^6^-methyladenosine (m^6^A) in mRNA

m^6^A is the most prevalent internal mRNA modification in eukaryotes, and its roles have been extensively studied in multiple eukaryotic systems (420). In 2015, using m^6^A immunoprecipitation followed by Illumina sequencing, m^6^A levels in gram-positive bacteria were measured and were found to be similar to levels previously reported in eukaryotic cells (26). *E. coli* showed 265 m^6^A peaks in 213 transcripts*,* whereas there were 109 m^6^A peaks in 68 *P. aeruginosa* transcripts. In both organisms, the transcripts bearing m^6^A were related to metabolism, transport, gene regulation, the cell envelope, ribosomes, stress responses, and nucleic acid synthesis (26). In 2024, a new study using MeRIP-seq together with DRS revealed 75 “high-confidence” m^6^A sites in 21 *E. coli* mRNAs and a total lack of m^6^A sites in *S. aureus* (421).

Despite these claims of m^6^A in bacterial mRNAs, another report of a DRS-based *E. coli* analysis did not recapitulate the results of the earlier studies (30). Although known m^6^A sites in the rRNA were accurately detected, no m^6^A was found in the mRNA. Furthermore, the single-nucleotide PCR technique SELECT was used to experimentally test eight of the previously reported “high-confidence” m^6^A sites in eight different mRNAs, with the results indicating a lack of m^6^A at those sites (30). Using MS, m^6^A abundance was evaluated in *E. coli* mRNA (30) and was calculated as less than 10% of previously reported levels (26). The presence of m^6^A in the mRNA was confirmed to be due at least in part to the activity of RlmF and RlmJ. Because these enzymes primarily catalyze m^6^A formation in the rRNA, m^6^A in the mRNA could be explained as off-target methylation by these enzymes, as has been demonstrated *in vitro* (30).

Despite recent challenges to the idea that m^6^A is present in *E. coli* mRNAs *in vivo*, several studies have been conducted to investigate the potential roles of m^6^A in prokaryotic mRNA. Another independent study employed m^6^A-seq and eTAM-seq to study m^6^A in *E. coli* mRNA. The results showed that the presence of m^6^A has no effect on mRNA expression or stability. Furthermore, variability in the data suggests that the modification is randomly distributed, lacking a specific enzyme or biological function (45).

**Fig 10:**
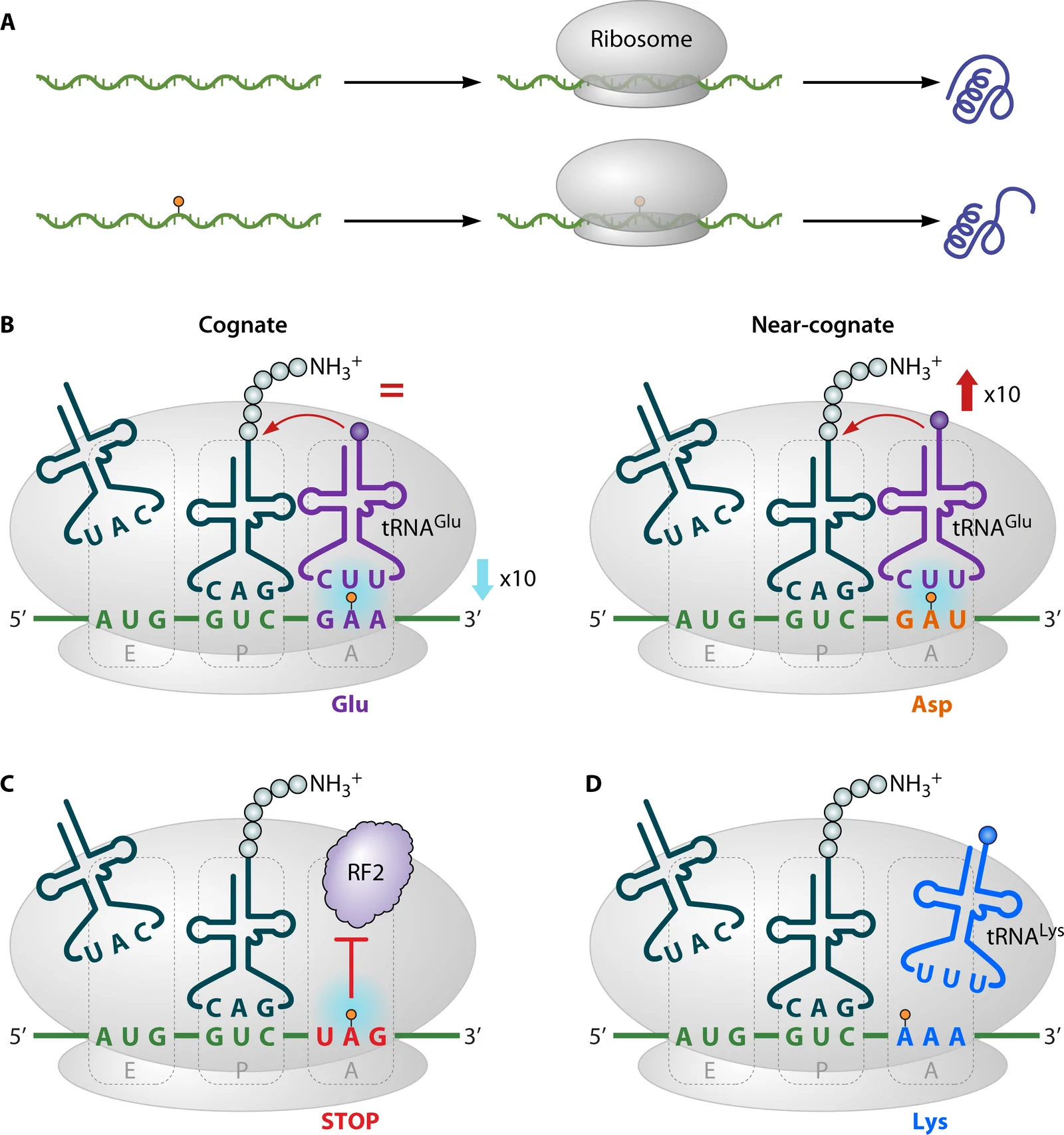
Roles of mRNA modifications in decoding. (**A**) Choi et al. (23) suggest that the presence of mRNA modifications affects the translation elongation process. Modifications could impact protein folding or interactions with chaperones, potentially influencing the functional form or localization of protein products. (**B**) The addition of m^6^A to cognate (GAA) or near-cognate (GAU) codons decreases anticodon reading by 10-fold; peptidyl transfer increases only in the near-cognate codon. This leads to a 10-fold increase in the amino acid substitution rate (23). (**C**) The presence of m^6^A decreases the interaction of release factor 2 (RF2) with the cognate (UAA) or near-cognate (UAG) stop codon by 6-fold or 3-fold, respectively (22). (**D**) The presence of m^6^A in the lysine codon enhances tRNA^Lys^ drop-off, which further delays peptide bond formation (24).

Additional *in vitro* studies have been conducted to establish the ways in which mRNA modifications could affect the decoding process (Fig. 10). The presence of m^6^A in an mRNA impedes tRNA interactions (likely due to destabilization of the m^6^A–U pairing), which affects translation elongation *in vitro* (23). Moreover, m^6^A has been associated with an increase in amino acid substitution errors and a decrease in reading accuracy at stop codons (22). Finally, the presence of m^6^A in mRNA inhibits completion of the decoding process by the ribosome, which is attributed to decreased stability of ribosome complexes (24). Thus, *in vitro* studies have provided compelling evidence that mRNA modifications significantly impact the translation machinery in *E. coli*.

### Inosine (I) in mRNA

I is an adenosine derivative that has been widely described in eukaryotic cells but was only recently identified in bacteria (422). In the anticodon of *E. coli* tRNA-Arg2, I is formed by the adenosine deaminase TadA. The same enzyme is also responsible for modifying the *E. coli* mRNA in at least 15 positions, 12 of which are in the coding region. Notably, many of these I positions are present in *hok* (host-killing toxin) genes. This I formation in *hok* transcripts leads to a Tyr-to-Cys substitution at position 29 of HokB and at position 46 of HokC, HokD, and HokE; the substitution at position 46 can also be found in *K. pneumoniae* and *Yersinia enterocolitica* (422). The presence of Cys at the HokB position 29 is associated with higher toxicity and bacterial death, potentially due to a disulfide bond generated by A-to-I editing. The latter phenomenon has also been observed in other pathogenic bacteria (423).

I formation is widespread across *γ-proteobacteria*, as evidenced by the identification of A-to-G mismatches in RNA-seq data from 72 bacterial species (424). As with the *E. coli hok* genes, these modifications were found to be present in coding regions, indicating that they may affect the corresponding proteins by altering amino acid sequences. Indeed, MS analysis of *Acinetobacter baylyi* proteins revealed the presence of two versions of several proteins encoded by I-containing mRNAs: one with the canonical amino acid sequence and the other containing a substitution caused by the presence of I in the transcript (424).

In some species, levels of I appear to be related to growth and metabolism. For example, the mRNA encoding a starch-degrading enzyme (*amyA*) shows varying abundance of I depending on the growth medium; I abundance is higher during growth with sucrose or glucose compared to fructose, maltose, or lactose (356). Similarly, analysis of *E. coli* grown in LB and minimal media (M9) and across different growth phases revealed 27 I sites (12 of which had not previously been reported) and found increased I levels during growth in LB compared to M9 and at the stationary phase compared to other growth stages (425).

In addition to metabolism, there is evidence that I abundance is associated with stress responses. *S. pyogenes* contains at least 27 I positions in the mRNA, and formation of the modification is temperature-dependent. Specifically, there are decreased and increased I levels observed at 42°C and 12°C, respectively, compared to 30°C or 37°C (356). Notably, a strain of *A. baylyi* carrying a less active version of TadA, TadA^(D54E)^, demonstrates impaired growth at 42°C compared to standard growth conditions (424). Together, these findings highlight the role of I formation in temperature-stress adaptation. In *S. pyogenes*, mRNAs corresponding to the zinc transporter gene *adcC* and the redox-defense gene *SPy_1219* have higher I levels after exposure to ZnSO_4_ or H_2_O_2_ (356). This demonstrates a dynamic, stress-responsive I formation system in some bacteria. Clinically, I has been found in *E. coli* and *P. aeruginosa* isolates from urinary tract infections (UTIs) and ear infections, respectively (426). Although this suggests a potential role of I in virulence, no association has been found between I abundance and antibiotic susceptibility or the clinical features of patients (426).

Despite the evidence that mRNA I abundance is stress-responsive in some species, the regulation of I in bacterial mRNA in general remains largely unclear. One study found that highly stable RNAs are more likely to contain I than transcripts with a more fleeting lifespan (356), suggesting a somewhat stochastic rather than targeted addition of I to mRNAs overall. Another group demonstrated that overexpression of tRNA-Arg2, which requires I in the anticodon loop, decreases I levels in the mRNA (425). This indicates that, at least in some species, TadA has lower affinity for mRNA than tRNA. Future studies are expected to shed additional light on the functions and regulatory mechanisms underpinning I formation in the mRNA.

### Pseudouridine (Ψ) in mRNA

The Ψ profile in *E. coli* mRNA was first comprehensively established using Pseudo-seq (180), which combines CMC treatment, size selection for truncated reverse-transcribed cDNA, and high-throughput sequencing. Using a collection of 11 known Ψ synthase mutants, researchers identified RluA as the enzyme responsible for modification of at least 31 different mRNA positions (180). This was particularly notable because RluA was previously characterized as a tRNA- and rRNA-specific Ψ synthase. Other rRNA Ψ synthases, such as RluC and RluD, were also found to modify mRNAs (180). Importantly, *fimA* transcripts (which encode the major pilus subunit) are less abundant in a ∆*rluA* mutant (180), suggesting that the presence of the modification is associated with mRNA stability.

A broader study using BID-seq evaluated several gram-negative species, including *B. cereus, E. coli*, *K. pneumoniae*, *P. aeruginosa*, and *Pseudomonas syringae,* under several growth conditions. In general, Ψ was found to be located in mRNAs related to central carbon metabolism, and the sequence motifs surrounding Ψ were conserved across all bacteria tested. A deeper analysis of *P. aeruginosa* in the exponential phase compared to the stationary phase showed enrichment of Ψ in mRNAs related to energy metabolism. In contrast, a set of genes involved in type IV swarming motility was enriched in Ψ during the stationary phase. Furthermore, mRNAs bearing Ψ were expressed at higher levels than unmodified mRNAs; these changes were ameliorated during growth in minimal medium (427). Ψ may therefore have a role in mRNA stability.

A new study using bisulfite treatment of *E. coli* mRNA identified Ψ in around 30% of the transcriptome (428), which is more abundant than previously reported (180). Ψ was found in transcripts related to the biosynthesis of specialized metabolites or adaptation to diverse environments, which suggests a potential role in stress responses. Pseudouridylated transcripts were less abundant in single deletion mutants for the Ψ synthetases *truB*, *truC*, and *truD* compared to the wild type, but not in the single *truA* deletion mutant. Furthermore, rifampicin treatment followed by RNA sequencing (RIF-seq) showed that the half-life of pseudouridylated mRNAs was shorter in the *∆truB*, *∆truC*, and *∆truD* mutants compared to the wild type, suggesting a role of Ψ in transcript stability. Finally, the bisulfite protocol was used to map Ψ in human oral microbiome samples, broadening the scope of the study (428).

### *N*^5^-methylcytidine (m^5^C) in mRNA

The presence of m^5^C in *E. coli* and *B. subtilis* was evaluated using bisulfite treatment coupled with RT and DNA sequencing (58). Although no m^5^C sites were found in mRNAs, the experiment had limited sequencing coverage of mRNAs, meaning that the presence of m^5^C could not be conclusively ruled out (58). Subsequently, DRS was successfully used to detect known m^5^C sites within the 16S and 23S rRNAs of *E. coli* (30). However, this study also did not detect m^5^C in any mRNA. The available data therefore do not support the presence of m^5^C in *E. coli* or *B. subtilis*, although future studies may demonstrate m^5^C in other bacterial species.

### Unspecified modifications in mRNA

The advent of DRS enabled the detection of many types of RNA modifications, although the modification identity cannot always be assigned based solely on nanopore data. Several research groups employing slightly different experimental approaches have studied mRNA modifications in *E. coli* MG1655 (30, 31, 75); together with a study focused on Ψ (180), these data provide the most detailed description to date of mRNA modifications in any bacterial species. The currently available data are summarized here as a comprehensive list of mRNA modification sites that have been reported in at least two studies (Table 4). Future studies employing methodologies such as DRS, single-nucleotide PCR, immunological assays, knockout mutants, and phenotypic characterizations are expected to greatly expand our understanding of the presence, identities, and functions of modifications in bacterial mRNAs.

**TABLE 4:** mRNA modifications observed in *E. coli* in multiple studies^*a*^

Gene name	Description	Detection method				Stress condition		
		Pseudo-Seq (Ψ) (180)	DRS nanoSundial (75)	DRS ELIGOS (30)	DRS nanoSundial + BC error (31)	Heat (45 °C)	Acid (pH 4.4)	Oxidative (2 mM H_2_O_2_)
*accC*	Biotin carboxylase		x		x			x
*acnB*	Aconitate hydratase B/2-methylisocitrate dehydratase	x	x	x	x	x		x
*acrB*	Multidrug efflux pump RND permease		x	x				
*aldA*	Aldehyde dehydrogenase A			x	x			
*aphA*	Acid phosphatase/phosphotransferase		x	x	x			
*bamD*	Outer membrane protein assembly factor		x		x			
*btsT*	Pyruvate:H + symporter		x	x				
*epd*	D-erythrose-4-phosphate dehydrogenase		x	x				
*fkpA*	Peptidyl-prolyl *cis-trans* isomerase/OMP chaperone		x	x		x		
*fur*	DNA-binding transcriptional dual regulator	x	x					
*hemN*	Coproporphyrinogen III dehydrogenase		x	x	x			
*hpf*	Ribosome hibernation-promoting factor	x			x			
*lpd*	Lipoamide dehydrogenase		x		x			
*narK*	Nitrate:nitrite antiporter		x		x			
*pgi*	Glucose-6-phosphate isomerase		x		x			
*rnpA*	RNase P protein component			x	x	x	x	x
*rpsA*	30S ribosomal subunit protein S1	x			x			
*rpsQ*	30S ribosomal subunit protein S17	x	x	x		x		
*secD*	Sec translocon accessory complex		x	x	x			
*sucB*	2-oxoglutarate dehydrogenase E2 subunit	x			x			x
*thrS*	Threonine–tRNA ligase		x	x		x		
*tufA*	Translation elongation factor Tu 1	x	x	x		x		
*tufB*	Translation elongation factor Tu 2	x			x			
*upp*	Uracil phosphoribosyltransferase		x	x				
*valS*	Valine–tRNA ligase		x	x		x		
*ybhQ*	Inner membrane protein			x	x			
*ygdR*	DUF903 domain-containing lipoprotein		x		x			
*yggX*	Putative Fe2+-trafficking protein		x		x			
*ygiC*	Spermidine ligase YgiC	x			x			

^*a*^ Summary of mRNA positions identified as modification sites in at least two of four studies. The Pseudo-Seq study used CMC and Illumina sequencing, identifying 38 pseudouridine sites (180). The first DRS study used FLO-MIN004RA data analyzed with nanoSundial, which produced 71 modification sites (75). The second DRS study (produced with an R9.4.1 flow cell) was analyzed with ELIGOS, revealing 283 putative novel modification sites/regions in the CDS (30). The last DRS study employed the FLO-MIN004RA platform, and the resulting data were analyzed with a combination of nanoSundial and BCError. This approach yielded 482 putative novel modification sites/regions in the coding sequence (31). Sites found in each stress condition were considered only if they were also present in the corresponding no-stress condition.

## CONCLUSIONS AND OUTLOOK

RNA modifications represent a recently recognized regulatory layer that affects numerous bacterial processes, from basal cellular functioning to complex stress responses. The impacts of modifications vary by identity and RNA subtype; although modifications in the tRNA, rRNA, and mRNA can all affect translation, the mechanisms of action differ. Furthermore, historical limitations in modification detection and a lack of experimental validation mean that our understanding of the scope and functions of RNA modifications remains in its infancy. Promising new developments in modification detection methods, including PCR- and DRS-based techniques, will enable rapid identification and validation of RNA modifications in all biotypes. Comparing modifications across cultures and stress conditions can provide further insight into the stress-related functions of specific modifications, highlighting a previously hidden regulatory layer.
